# Limited efficacy of APRIL CAR in patients with multiple myeloma indicate challenges in the use of natural ligands for CAR T-cell therapy

**DOI:** 10.1136/jitc-2023-006699

**Published:** 2023-06-30

**Authors:** Lydia Lee, Wen Chean Lim, Daria Galas-Filipowicz, Kent Fung, Julia Taylor, Dominic Patel, Zulaikha Akbar, Elena Alvarez Mediavilla, Patrycja Wawrzyniecka, Debarati Shome, Rogier M Reijmers, Trillian Gregg, Leigh Wood, William Day, Virginie Cerec, Mathieu Ferrari, Simon Thomas, Shaun Cordoba, Shimobi Onuoha, Nushmia Khokhar, Vijay Peddareddigari, Muhammad Al-Hajj, Jim Cavet, Sonja Zweegman, Manuel Rodriguez-Justo, Kwee Yong, Martin Pule, Rakesh Popat

**Affiliations:** 1 Research Department of Haematology, UCL Cancer Institute, London, UK; 2 Autolus Ltd, London, UK; 3 Department of Pathology, UCL Cancer Institute, London, UK; 4 Lumicks, Amsterdam, The Netherlands; 5 Department of Haematology, University College London Hospitals NHS Foundation Trust, London, UK; 6 The Christie NHS Foundation Trust, Manchester, UK; 7 Vrije Univ Amsterdam, Amsterdam, The Netherlands

**Keywords:** clinical trials as topic, immunotherapy, immunotherapy, adoptive, T-lymphocytes

## Abstract

**Background:**

We used a proliferating ligand (APRIL) to construct a ligand-based third generation chimeric antigen receptor (CAR) able to target two myeloma antigens, B-cell maturation antigen (BCMA) and transmembrane activator and CAML interactor.

**Methods:**

The APRIL CAR was evaluated in a Phase 1 clinical trial (NCT03287804, AUTO2) in patients with relapsed, refractory multiple myeloma. Eleven patients received 13 doses, the first 15×10^6^ CARs, and subsequent patients received 75,225,600 and 900×10^6^ CARs in a 3+3 escalation design.

**Results:**

The APRIL CAR was well tolerated. Five (45.5%) patients developed Grade 1 cytokine release syndrome and there was no neurotoxicity. However, responses were only observed in 45.5% patients (1×very good partial response, 3×partial response, 1×minimal response). Exploring the mechanistic basis for poor responses, we then compared the APRIL CAR to two other BCMA CARs in a series of in vitro assays, observing reduced interleukin-2 secretion and lack of sustained tumor control by APRIL CAR regardless of transduction method or co-stimulatory domain. There was also impaired interferon signaling of APRIL CAR and no evidence of autoactivation. Thus focusing on APRIL itself, we confirmed similar affinity to BCMA and protein stability in comparison to BCMA CAR binders but reduced binding by cell-expressed APRIL to soluble BCMA and reduced avidity to tumor cells. This indicated either suboptimal folding or stability of membrane-bound APRIL attenuating CAR activation.

**Conclusions:**

The APRIL CAR was well tolerated, but the clinical responses observed in AUTO2 were disappointing. Subsequently, when comparing the APRIL CAR to other BCMA CARs, we observed in vitro functional deficiencies due to reduced target binding by cell-expressed ligand.

**WHAT IS ALREADY KNOWN ON THIS TOPIC**

- Dual targeting chimeric antigen receptor (CAR) constructs may address the challenges of low target tumor expression and the possibility of antigen negative escape in multiple myeloma. The APRIL CAR is a ligand-based, dual targeting CAR able to target two myeloma cell antigens, B-cell maturation antigen (BCMA) and transmembrane activator and CAML interactor.

**WHAT THIS STUDY ADDS**

- Clinical responses from this Phase 1 trial in relapsed refractory multiple myeloma were disappointing prompting a series of reverse translation experiments of the APRIL CAR in direct comparison to other BCMA CAR constructs. In this unique exploration for the reason underpinning suboptimal clinical responses, we find many similarities in the in vitro activity of the APRIL CAR in direct comparison to two other BCMA CARs except for reduced interleukin-2 secretion and lack of sustained tumor control. We ultimately attribute poor efficacy to the APRIL binder itself which binds target poorly when expressed on the surface of T-cells.

**HOW THIS STUDY MIGHT AFFECT RESEARCH, PRACTICE OR POLICY**

- On a practical level, we establish simple in vitro experimentation which may be informative as regards clinical performance. Further the investigation of the APRIL CAR continues preclinically and in clinical trials and there will only be greater exploration in the field of dual targeting CAR constructs. This manuscript focuses exclusively on the APRIL CAR and throws caution to the pursuit of APRIL, and possibly other ligand-based CAR constructs in the future development of effective, dual-targeting CARs that will benefit patients.

## Introduction

Despite advances, multiple myeloma (MM) remains an incurable and common cancer characterized by sequential relapses requiring retreatment. Patients inevitably develop resistance to multiple therapies at which point their prognosis is poor. Notably, CD19 chimeric antigen receptor (CAR) T-cell therapies can achieve durable complete responses (CR) in a proportion of patients with relapsed, refractory B-cell malignancies.^1 2^ Although development of CAR T-cell therapy in MM is still early compared with that in B-cell malignancies, several studies show high remission rates and durable responses.^3^

CAR T-cell therapy in MM have mainly targeted the B-cell maturation antigen (BCMA), a member of the tumor necrosis factor receptor superfamily (TNFRS), which is selectively expressed on mature B cells and plasma cells (PC) as well as tumor cells from the majority of patients with MM.^4^ When CAR T-cells against BCMA were first considered a decade ago, some limitations were anticipated: first, BCMA expression on the surface of myeloma cells is significantly less than the number of CD19 molecules expressed on the surface of acute lymphoblastic leukemia blasts.^1 5^ This might lead to incomplete signaling and limited expansion and persistence. Further, target downregulation is frequently observed in patients treated with CD19 and CD22 targeting therapies^6 7^; the possibility of BCMA modulation was also anticipated and reported in the earliest use of a BCMA CAR in patients.^8^

Hence to increase the level of targetable tumor antigen and address the potential for antigen negative tumor escape, we developed the APRIL CAR for the treatment of MM. In comparison to conventional CAR constructs which typically employ antibody-based binders specific for tumor antigens, the APRIL CAR was based on a proliferating ligand (APRIL)—the natural ligand for BCMA. APRIL also recognizes transmembrane activator and CAML interactor (TACI), also a member of the TNFRS and expressed on B cells and PC.^5^ Given that both BCMA and TACI were recognized, the total targetable antigen density detected by APRIL CAR was increased and targeting two antigens should also reduce antigen escape. In a preclinical study, we observed the cytotoxicity of APRIL CAR T-cells against cell lines expressing physiological levels of BCMA and TACI as well as primary tumor cells, maintained target kill in the presence of soluble BCMA, TACI or APRIL, and finally, rapid clearance of tumor in an in vivo myeloma model.^5^

On the basis of these data, we designed a Phase 1 clinical trial of APRIL CAR T-cells in patients with refractory MM (NCT03287804, AUTO2). We observed low toxicity, low engraftment but response rates were low. In contrast, contemporaneous studies targeting BCMA with standard CAR designs showed high response rates, although with modest CAR persistence. Notably, BCMA loss has been shown to be an infrequent occurrence.^9 10^ Subsequently, we went from the bedside back to the bench and compared the in vitro characteristics of the APRIL CAR with those of other BCMA CARs with established clinical efficacy to identify key differences which might explain poor performance in patients. In this paper, we describe results of the AUTO2 clinical study and this subsequent exploration.

## Materials and methods

### Participants and study design

This open-label, dose escalation Phase 1 study was conducted in University College London Hospital, London, UK; The Christie NHS Foundation Trust, Manchester, UK; Amsterdam UMC, Cancer Center Amsterdam, Amsterdam, The Netherlands and The Freeman Hospital, Newcastle Hospitals NHS Foundation Trust, UK. Eligibility criteria included an age of 18 years or older; an Eastern Cooperative Oncology Group performance-status score of 0 or 1; measurable disease, defined by a concentration of monoclonal protein in serum of at least 5 g/L or in urine of at least 200 mg/24 hours, serum-free light chains (involved free light chain concentration of ≥100 mg/L with abnormal ratio); at least three previous lines of therapy, including a proteasome inhibitor (PI), an immunomodulatory drug (IMID), an alkylator or CD38 monoclonal antibody (MoAB) or disease refractory to both PIs and IMID; peripheral lymphocyte count of >0.5×10^9^/L, creatinine clearance (Cr/Cl) >30 mL/min as well as adequate hepatic and cardiac function. Patients with central nervous system (CNS) disease, prior allogeneic stem cell transplant, were excluded. Tumor expression of BCMA and TACI was not an exclusion factor.

Patients were administered lymphodepletion with fludarabine (30 mg/m^2^/day) and cyclophosphamide (300 mg/m^2^/day) on days –6, –5, and –4, followed by an infusion of APRIL CAR on day 0. Dose escalation initially followed an accelerated dose titration design, in which a single patient was dosed at 15×10^6^ CAR T-cells, followed by further CAR doses (75,225,600 and 900×10^6^ CAR T-cells) in a 3+3 escalation design. After completion of the 24-month follow-up period or following AUTO2 treatment and early withdrawal, all patients are followed until death or for up to 15 years from treatment administration. The study was conducted in accordance with the Declaration of Helsinki and International Conference on Harmonization guidelines for Good Clinical Practice and all applicable national and local laws and regulations for clinical research at each center. Written informed consent was obtained from each patient.

### End points and assessments

Primary outcome measures were incidence of adverse events graded according to the National Cancer Institute Common Terminology Criteria for Adverse Events V.4.03, abnormal laboratory test results, and dose-limiting toxicities as defined below. Secondary outcome measures include disease-specific response criteria (according to the International Myeloma Working Group Uniform Response Criteria for Multiple Myeloma from the day of CAR infusion) and measurement of tumor BCMA expression by immunohistochemistry (IHC) performed as previously described.^1^ The exploratory end point progression-free survival (PFS) was defined as the time from CAR infusion to the date of either the first observation of progressive disease or death of any cause.

#### Retroviral vector and CAR T-cell manufacture

APRIL CAR has been previously described.^5^ Briefly, APRIL CAR was constructed by fusing APRIL, with a deleted proteoglycan binding domain, to the human IgG1 hinge and then to the CD28 transmembrane domain and the endodomains of CD28, OX40 and CD3-ζ. APRIL CAR was coexpressed in the γ-retroviral vector SFG^5^ with the sort-suicide gene RQR8^11^ using a foot-and-mouth like 2A peptide sequence from *thosea asigna* virus.^12^ RQR8/APRIL CAR encoding γ-retroviral vector was generated by transfecting 293 T-cells with the SFG plasmid, and plasmids encoding the RD114 envelope and MoMLV gagpol. Supernatant was purified by anion exchange chromatography and filtration. CAR T-cells were generated from autologous peripheral blood mononuclear cells harvested by leukapheresis. Leukapheresate was stimulated with transact (Miltenyi), transduced with the γ-retroviral vector on retronectin (Takara), then transferred to the Miltenyi prodigy and expanded in interleukin (IL)-7 and IL-15 for 7–10 days following which cell product was cryopreserved in dimethyl sulfoxide(DMSO). Transduction efficiency was determined by fluorescence activated cell sorting (FACS) staining of T-cells for RQR8 marker expression.

### FACS analysis

Bone marrow mononuclear cells (BM MNCs) were isolated by Ficoll Paque. Vials of stored BM MNCs or manufactured CAR products were defrosted and stained with antibodies as specified in supplementary data before analysis with a Fortessa (BD) and FlowJo (V.10.6).

### Statistical analyses

Unless otherwise stated, data are expressed as mean±SE, and analyses were performed in GraphPad Prism, V.9 as specified in the body of this manuscript. P value<0.05 was deemed statistically significant. Data supporting the findings of this study are available on request from the corresponding author.

Further trial details and methods available in online supplemental data.

10.1136/jitc-2023-006699.supp1 Supplementary data

## Results

### Study participant and disease characteristics

We tested autologous APRIL CAR T-cells in a Phase 1 dose-escalation study of relapsed refractory MM. Twelve subjects were enrolled (table 1, online supplemental table 1). Successful harvest and manufacture of target dose was achieved for all patients but one patient withdrew prior to treatment due to disease progression.

10.1136/jitc-2023-006699.supp2 Supplementary data

**Table 1:** Summary of AUTO2 patient demographics

Total treated	11
Sex	
Male	8 (72.7%)
Female	3 (27.4%)
Age (median/range)	61(45–69)
Isotype	
IgG	9 (81.8%)
LC	2 (18.2%)
ISS at presentation	
I	6 (54.5%)
II	1 (9.1%)
III	3 (27.3%)
Unknown	1 (9.1%)
Cytogenetics	
High risk	4 (36.4%)
Standard risk	2 (18.2%)
Unknown	5 (45.5%)
Years since diagnosis (median/range)	6 (1–11)
Extramedullary disease	3 (27.3%)
Previous therapy	
Lines (medium/range)	5 (3–6)
Previous ASCT	6 (54.5%)
Anti CD38 exposed	6 (54.5%)
Progressed on last line	5 (45.5%)
Refractory to PI or IMID	11 (100%)
Refractory to anti CD38	5 (45.5%)
Double refractory (PI and IMID)	9 (81.8%)
Triple refractory (PI, IMID, CD38)	3 (27.3%)

Refractory, progressed on or within 60 days of receiving these agents. High risk cytogenetics defined as t(4;14), t(14;16), t(14;20), del(17p), 1q gain, 1p loss.

ASCT, autologous stem cell transplant; IMID, immunomodulatory imide; ISS, International Staging System; LC, Light Chain; PI, proteasome inhibitor.

Of the 11 patients treated, the median age was 61 (range 41–69). Three (27.3%) of patients had International Staging System (ISS) stage III disease at diagnosis and one (11.1%) at screening, four (36.4%) had high risk cytogenetics (defined as t(4;14), t(4;16), t(4;20), del(17p) (≥50% of total nucleated cells), 1q gain, 1p loss) and three (27.3%) had extramedullary (EM) disease. Patients had received a median of 5 prior therapy lines (range, 3–6). All patients had received a PI and IMID to which nine (81.8%) were double refractory. Over half (54.5%) had received daratumumab, three patients (27.3%) were refractory to PI, IMID and daratumumab and two (18.2%) were penta-refractory (bortezomib, carfilzomib, lenalidomide, pomalidomide and a CD38 MoAB). None of the patients in the AUTO2 cohort had received a BCMA targeting therapeutic agent prior to enrollment. Six (54.5%) of the patients had received a previous autologous stem cell transplant (table 1, online supplemental table 1). Four patients received bridging therapy between leukapheresis and CAR T-cell infusion (online supplemental table 2) all bridged patients had stable or progressive disease between initial screening and start of lymphodepletion and still had measurable disease. Baseline renal function ranged from a Cr/Cl of 55–133 mL/min (median 85).

**Table 2:** Summary of adverse events

	Any grade	Grade 3	Grade 4
Any adverse event	11 (100%)	11 (100%)	11 (100%)
Hematology			
Neutropenia	11 (100%)	0	11 (100%)
Anemia	8 (72.7%)	8 (72.7%)	0
Thrombocytopenia	4 (36.4%)	0	3 (27.3%)
Lymphopenia	1 (9.1%)	0	1 (9.1%)
Gastrointestinal			
Dysgeusia	2 (18.2%)	0	0
Mucositis	3 (27.3%)	0	0
Nausea	7 (63.6%)	0	0
Vomiting	2 (18.2%)	1 (9.1%)	0
Diarrhea	5 (45.5%)	0	0
Constipation	5 (45.5%)	0	0
Abnormal liver function tests	2 (18.2%)	0	0
Respiratory			
Dyspnea	5 (45.5%)	1 (9.1%)	0
Cough	3 (27.3%)	0	0
Cardiovascular			
Hypotension	1 (9.1%)	0	0
Peripheral edema	4 (36.4%)	0	0
MI	1 (9.1%)	1 (9.1%)	0
Skin			
Rash	2 (18.2%)	0	0
Pruritus	1 (9.1%)	0	0
Neurology			
Dizziness	1 (9.1%)	0	0
Parasthesia	2 (18.2%)	0	0
Headache	5 (45.5%)	1 (9.1%)	0
Infections (any)	9 (81.8%)	5 (45.5%)	0
Other			
Fatigue	9 (81.8%)	0	0
Fevers	6 (54.5%)	2 (18.2%)	0
Chills	4 (36.4%)	0	0
Body or joint pain	7 (63.6%)	2 (18.2%)	0
Low calcium	1 (9.1%)	1 (9.1%)	0
Low phosphate	1 (9.1%)	1 (9.1%)	0
CRS	5 (45.5%)	0	0
Macrophage activation syndrome	1 (9.1%)	0	0
ICANS	0	0	0

CRS, cytokine release syndrome; ICANS, immune effector cell associated neurotoxicity syndrome.

Baseline tumor expression of BCMA and TACI were assessed by FACS^3^ and IHC (online supplemental table 3). Treated patients had a median surface expression level of BCMA and TACI of 596 (430–780) and 381 (0–1819) antigens bounds per cell (ABC), respectively, from BM tumor cells at study entry and antigen expression was maintained following CAR T-cell infusion as measured from 1 month and at disease progression (online supplemental table 3).

### Toxicity and serum cytokines

Shown are adverse events not designated as symptoms of cytokine release syndrome (CRS) that occurred in the first 60 days of a dose of APRIL CAR T-cells. CRS was graded according to the criteria in Lee *et al.* 11

Five (45.5%) patients developed CRS, all of which were mild (Grade 1^11^). Three patients developed CRS within the first 2 weeks (Patients 003, 011 and 012 on days 11, 9 and 0, respectively) and one patient developed CRS late (Patient 001, day 26). A further patient developed CRS early with fevers on day of infusion and subsequently macrophage activation syndrome at day 29 which responded to tociluzimab. Three patients received a single dose of tociluzimab and steroids were not administered post CAR T-cells. There were no instances of immune effector cell associated neurotoxicity syndrome (ICANS) (table 2). A rise in interferon-γ (IFN-γ) was only detected in the four patients receiving the highest doses of CAR T-cells (Patients 009, 010, 011, 012) to a maximum of 2984 pg/mL (online supplemental figure 1). IL-6 rose to 413 pg/mL (median 64 pg/mL).

All patients experienced Grade 4 neutropenia and 72% Grade 3 anemia. Duration of cytopenias could be prolonged. Grade 3 or 4 events were observed beyond 30 days in the following numbers of patients: anemia 5; thrombocytopenia 3; neutropenia 7. And beyond 90 days in the following: thrombocytopenia 2; neutropenia 3. Patients were supported with transfusions and granulocyte colony stimulating factor at their physicians discretion. However, excluding hematological toxicity, eight patients experienced Grade 3 or higher toxicity of any cause of which five patients experienced Grade 3 infections and there were no AUTO2 related deaths (online supplemental table 4). One patient receiving the highest dose of CAR T-cells experienced a myocardial infarction on day of CAR infusion that was thought possibly attributed to APRIL CAR infusion due to the temporal nature of the adverse event in relation to treatment. Thus, we did observe cytopenias and infections associated with lymphodepletion and, overall, APRIL CAR was well tolerated with a low incidence of CRS and no reported cases of ICANs.

### Product characterization and CAR T-cell persistence

The median transduction efficiency (TE) was 23% (range 5.81–40.2%, online supplemental table 2) and the CD4:CD8 ratio in the T-cell product varied (median 2.8, range 0.3–12.9, online supplemental figure 2A) with a predominance of CD4. The majority of CD4 cells were effector memory (EM) (median 74.8%, range 41.3–90.0%, online supplemental figure 2B) while the CD8 CAR T-cells had a smaller proportion of EM cells (median 50.1, range 16.4–76%) and more terminally differentiated EM cells re-expressing CD45RA (TEMRA) cells (median 27.1%, range 3.4–61%). There was a low proportion of central memory (CM) CD4 and CD8 APRIL CAR T-cells (median 11.9 and 9.4%, respectively).

**Figure 1:**
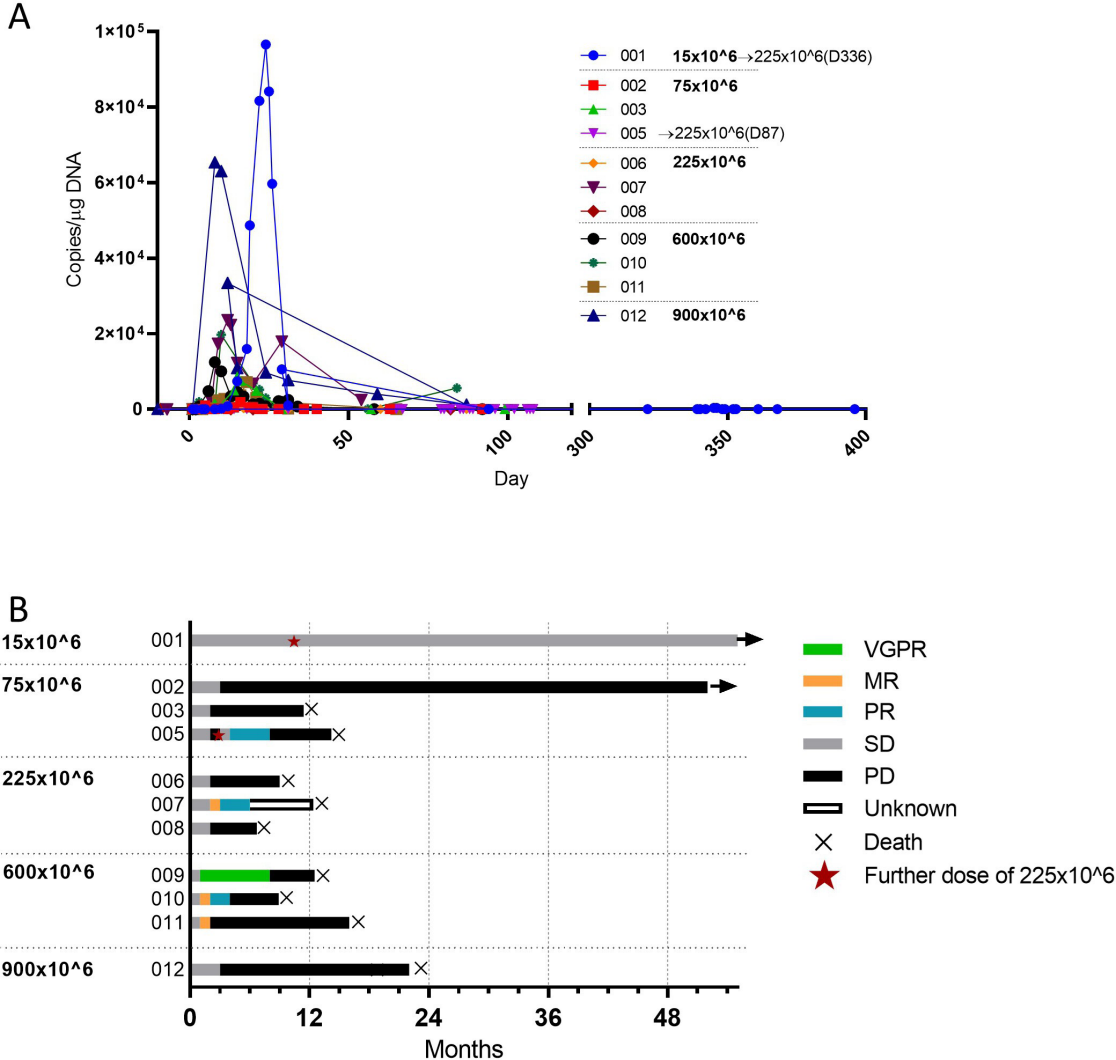
CAR expansion and best response. (A) CAR T-cell expansion as assessed by PCR of peripheral blood. (B) Best response to APRIL CAR infusion (as of February 2022) according to dose (15×10^6^ to 900×10^6^) of chimeric antigen receptor–positive (CAR+) T-cells. Two patients were retreated with a higher dose of 225×10^6^ cells at time points indicated with a red star. All responses were confirmed and assessed according to the International Myeloma Working Group Uniform Response Criteria for Multiple Myeloma. MR, minimal response; PR, partial response; SD, stable disease; PD, progressive disease; VGPR, very good partial response.

Circulating APRIL CAR was detectable following 12/13 doses administered (figure 1A) within the first 10 days (median 5, range 1–10) and peaked early (median 12 days, range 8–24 days) with a median Tmax of 8673.7 copies/µg DNA (range 26–96,598 copies/µg DNA). APRIL CAR T-cell expansion or tracking to tumor niches was not associated with dose (figure 1A, online supplemental figure 3A–C). APRIL CAR was typically undetectable by 30 days, had a median persistence of 21 days and detectable up to 3 months in one patient at the highest dose. In this small cohort, APRIL CAR expansion did not correlate with disease response with no clinical responses observed with APRIL CAR expansion (Patients 001, 011 and 012) and vice versa (Patient 005).

At 1-month post treatment, APRIL CAR T-cells were detected by flow cytometry in BM in five patients (online supplemental figure 3B–D). Compared with therapeutic product, there was a preferential expansion of CD8 CAR T-cells, increase in mature memory phenotypes as well as increased expression of immunomodulatory proteins (online supplemental figure 3F–G). There was an increased expression of Programmed cell death protein 1 (PD-1) (p<0.05 for CD4 and CD8 CAR T-cells by paired t-test) and a trend for increased T-cell immunoglobulin and mucin-domain containing-3 (TIM3) expressing CD4 and CD8 T-cells (p=0.06, p=0.07, respectively, by paired t-test). Further, APRIL CAR T-cells expressed more TIM3 and PD-1 and less Ki67 compared with non-CAR T-cells from patient BM samples (online supplemental figure 3H).

### Disease response

The objective response rate was low and short lived (figure 1B). Five (45.5%) patients responded. One patient achieved a very good partial response (VGPR) which included regression of EM disease, 3 a partial response (PR) and 1 a minimal response. Of these patients, median PFS was 5 months (range 2–8). As of February 1, 2022, median time to progression was 3 months following APRIL CAR, 2 patients were still alive and the median overall survival for the 11 patients was 375 days following the first APRIL CAR T-cell dose. Patient 001 has remained in stable disease 55 months after receiving CAR T-cells.

### Retreatment with APRIL CAR

Expecting a higher therapeutic dose, two patients who received the lowest doses of CAR T-cells were retreated with a higher dose of APRIL CAR (225×10^6^). Patient 1 had a significant CAR T-cell expansion following a dose of 15×10^6^ cells (nearly 1×10^5^ copies/µg genomic DNA (gDNA)) but stable disease. Following the second infusion 11 months later, there was significantly reduced CAR T-cell expansion (to 4×10^2^ copies/µg gDNA) without disease response. Patient 5 had progressive disease at 1 month following the initial dose of 75×10^6^ cells and received four doses of weekly daratumumab from month 2. There was a PR following a second CAR T-cell dose at month 3 and low level CAR expansion (10–100 copies/µg) following both CAR doses.

We previously described activity of APRIL CAR T-cells against TACI and BCMA expressing targets in vitro and in vivo including in response to low-density target cells. Despite this, the APRIL CAR demonstrated inferior performance to other BCMA CARs.^13 14^ We undertook a series of reverse translation experiments to understand the reasons for this.

### APRIL CAR T-cells are deficient in IL-2 secretion and activity on repeated challenge

**Figure 2:**
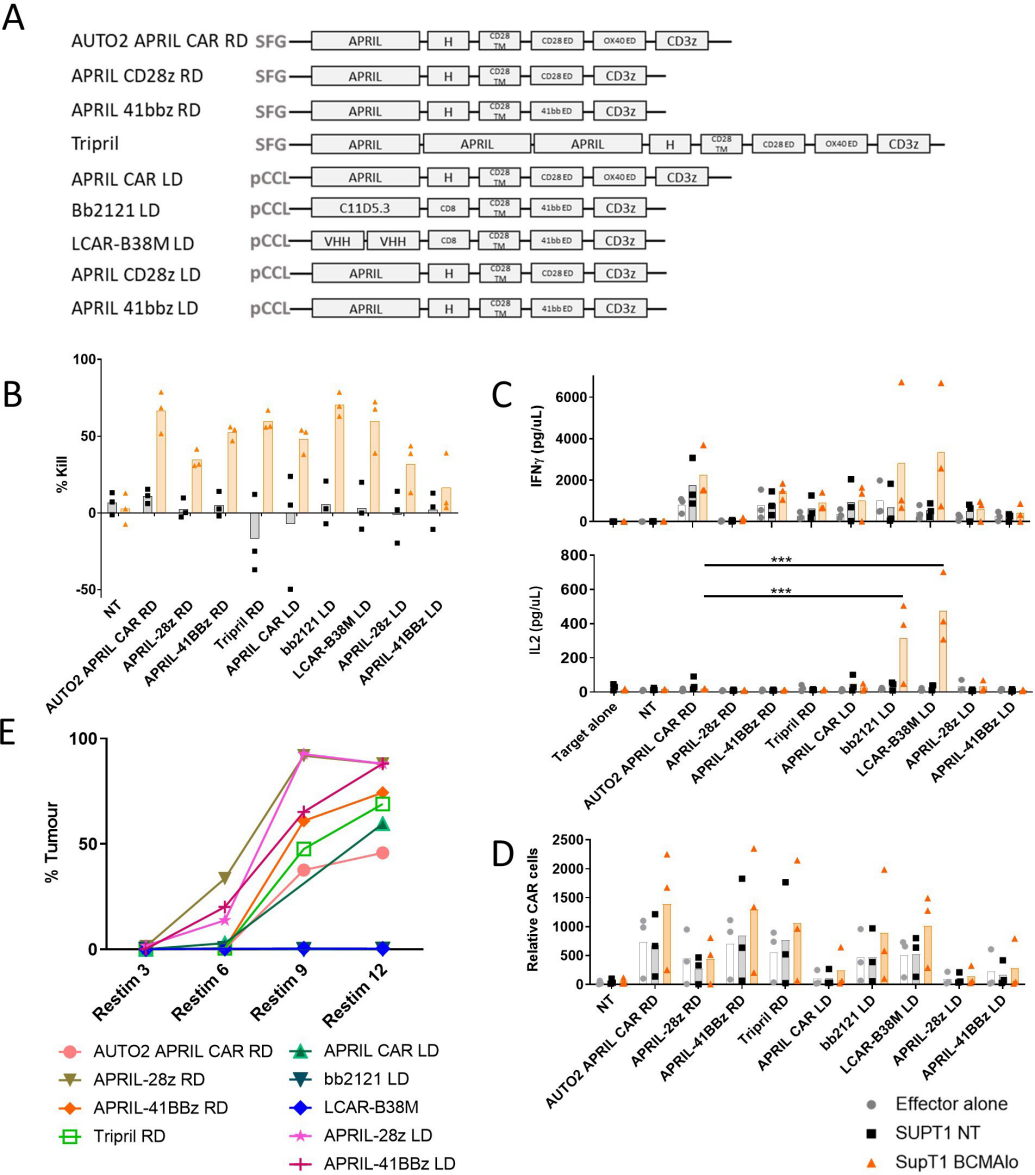
Functional assessment of APRIL CAR variants, bb2121 and LCAR-B38M CAR in vitro. CAR transduced peripheral blood mononuclear cells from normal donors (n=3) were co-cultured with non-transduced SUPT1 targets (SUPT1 NT) or targets engineered to express low levels of BCMA (estimated 636 molecules per cell SUPT1 BCMA) at an effector to target ratio (E:T) of 1:4. (A) Diagram summarizing various CAR constructs assessed functionally. (B) Target kill as a percentage of targets in media alone. (C) IFN-γ and IL-2 release as assessed by ELISA of culture supernatant at 24 hours. (D) After 4 days of co-culture, CAR T-cells co-cultured alone, with SUPT1 NT or SUPT1 BCMAlo targets were enumerated. (E) Next CARs were also co-cultured with MM1s cells at an E:T ratio of 1:4 and further live MM1s cells added to wells of culture plate twice a week from 4 days after initial co-culture set-up (=ReStim 1). Assessment of viable cells in culture plate occurred 3 days after number of restimulations indicated and percentage tumor of total live cells after sequential stimulations displayed on graph. Data shown is from single experiment, with each data point representing mean of duplicate or triplicate. In B–D, effector alone, SUPT1 NT and SUPT1 BCMA are represented by gray circles, black squares and orange triangles, respectively. Statistical tests by one-way analysis of variance with focus on performance of AUTO2 and bb2121 or LCAR-B38M. ***p<0.001. BMCA, B-cell maturation antigen; CAR, chimeric antigen receptor; IFN, interferon; IL, interleukin; LD, lentivirus; NT, non-transduced; RD, retrovirus.

We compared the function of APRIL CAR to what has become standard BCMA targeting CARs. The sequences for the standard single chain variable fragment (scFv) used in bb2121^15–17^ and two VHH domains in LCAR-B38M^18 19^ were derived from patents, and the latter joined by a (G_4_S)_3_ linker. Both BCMA binders were then cloned into second generation (41bbζ) backbones and henceforth referred to as bb2121 and LCAR-B38M CARs, respectively (figure 2A).

The APRIL CAR varies from most other BCMA CARs not only by its ligand-based binder, but also by having a third generation (CD28-OX40-CD3ζ) endodomain, and use of γ-retroviral vector for manufacture. Hence, to also understand if these differences impacted on relative performance, along with AUTO2 (the therapeutic: γ-retroviral transduction, third generation CD28-OX40-CD3ζ endodomain), we also compared APRIL CAR in second generation formats (CD28ζ, 41BBζ endodomains) and manufactured in lentiviral vectors. CAR T-cells were cultured alone, with unmodified SUPT1 or SUPT1 targets expressing low levels of BCMA (636 ABC).

We observed equivalent target kill, IFN-γ and cell proliferation at 5 days on co-culture with antigen expressing target of the γ-retroviral, third generation APRIL CAR compared with BCMA CARs. These parameters were not improved by APRIL CAR transduction method or co-stimulatory endodomain (figure 2B–D). However, one notable difference of the APRIL CAR was the low levels of IL-2 release of all APRIL CAR formats compared with bb2121 and LCAR-B38M on co-culture with BCMA expressing targets (figure 2C). IL-2 secretion by APRIL constructs remained comparatively deficient on co-culture with targets expressing increased levels of BCMA (online supplemental figure 4) or on altering the extracellular linker of the APRIL CAR construct (online supplemental figure 5). IL-2 secretion on co-culture with BCMA expressing targets could be increased by increasing TE and therefore increasing CAR expression on T-cells (online supplemental figure 6). Second, despite equivalent target specific kill on co-culture, and in contrast to bb2121 and LCAR-B38M, notably, all APRIL CARs failed to control tumor growth on repeated in vitro stimulation with MM1s cells (figure 2E).

**Figure 5:**
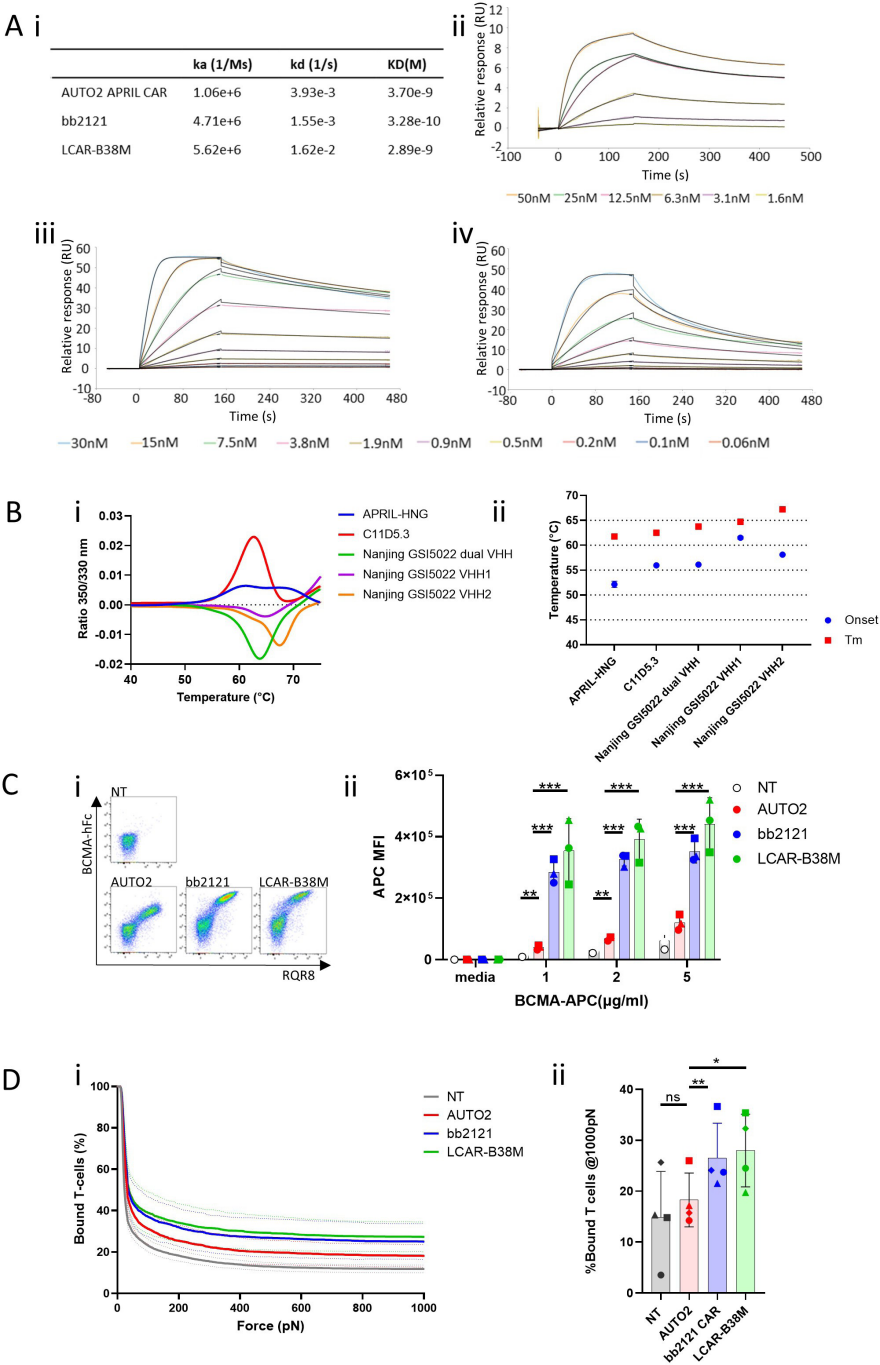
Characteristics of AUTO2, bb2121 and LCAR-B38M BCMA binders. (A) Binding kinetics of BCMA binders as assessed by surface plasmon resonance. IgG2a Fc conjugated binders were immobilized on a CM5 biacore chip before binding assessment with soluble BCMA. (i) Summary table. Binding for (ii) WT APRIL, (iii) C11D5.3, (iv) VHH binders used in LCAR-B38M (Nanjing_GSI5022_Dual_VHH). (B) Protein stability of BCMA binders as determined by nano differential scanning fluorimetry (nanoDSF) (i) Thermal unfolding curves, (ii) graph plotting onset of unfolding as well as melting temperatures of binder used in AUTO2 (APRIL-HNG), bb2121(C11D5.3) and LCAR-B38M (two VHH in series/Nanjing GSI5022 dual, and each VHH in isolation/VHH1 or VHH2). (C) Binding of CAR expressing cells to soluble ligand were assessed by transducing peripheral blood mononuclear cells from three healthy donors with bicistronic constructs coexpressing RQR8 marker gene (in format RQR8_2A_CAR) before incubation with (i) BCMA Fc and secondarily stained with APC conjugated anti Fc antibody (eg, FACS plots from a single donor shown). (ii) Alternatively, CARs were incubated with different concentration with APC conjugated BCMA. Controlling for set MFI of RQR8 expression, graph showing APC MFI of transduced T-cells. (D) Cell avidity between CAR transduced T-cells from four healthy donors (mean of minimum of two replicates shown) and myeloma cell line H929 assayed by acoustic force microfluidic microscopy. (i) Avidity curves represent mean±SEM from separate donors. (ii) Cell binding avidity from h at 1000 pN. Individual donors represented with different shapes. Multiple paired t-tests and Holm-Sidak correction. *p<0.05, **p<0.01, ***p<0.001. APC, Allophycocyanin; BMCA, B-cell maturation antigen; CAR, chimeric antigen receptor; FACS, fluorescence activated cell corting; Fc, fragment crystallizable; MFI, mean fluorescence intensity; NT, non-transduced.

These observations of reduced IL-2 secretion on co-culture and failure of prolonged tumor control were seen in all APRIL CAR formats regardless of co-stimulation domains or γ-retroviral versus lentiviral transduction.

### Reduced cytokine signaling on stimulation of APRIL CAR by RNA sequencing

We then looked for differences in T-cell activation by interrogating the transcriptomic profiles obtained by bulk RNA sequencing of non-transduced (NT) T-cells from three donors, and T-cells transduced with APRIL CAR, bb2121 or LCAR-B38M following in vitro activation by plate bound BCMAFc for 24 hours.

**Figure 3:**
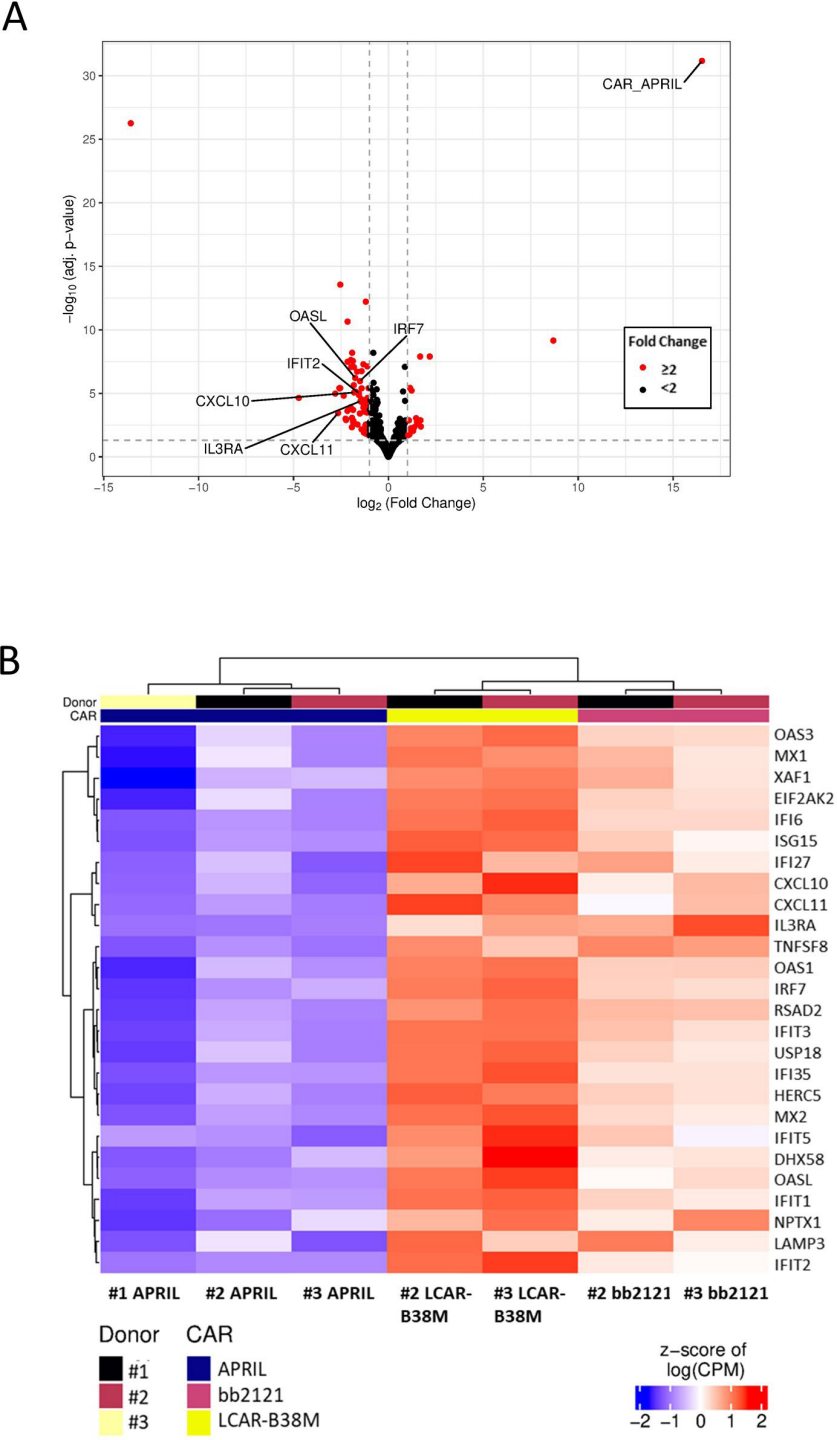
RNA sequencing of ligand activated CAR T cells from different donors. (A) Volcano plot of differentially expressed genes in APRIL versus bb2121 and LCAR-B38M (to the left, genes upregulated in bb2121/LCAR-B38M). Genes highlighted have greater than twofold difference in expression and adjusted p<0.05 by DESeq2. (B) Heatmap of selected differentially expressed genes (adjusted p<0.05 by DESeq2). CAR, chimeric antigen receptor.

There was evidence of antigen-dependent T-cell activation in all three BCMA targeting CARs compared with NT T-cell with increased T-cell receptor (TCR) signaling, cytokine signaling and cell cycling (online supplemental figure 7). Comparing the three BCMA targeting CAR constructs, we did observe a small number of transcripts significantly expressed at a lower frequency in APRIL CAR compared with bb2121 and LCAR-B38M (n=86 with >log2 fold change in expression and adjusted p<0.05, figure 3A, B). Interestingly, at 24 hours there was no difference in IRF4, which is thought to correlate to strength of TCR activation,^20^ or the cytokines IFN-γ or IL-2. However, there was evidence of reduced cytokine signaling. Many of the genes significantly upregulated in bb2121/LCAR-B38M are involved in type 1 (eg, IRF7, MX1, OAS genes) and type 2 IFN (eg, CXCL10) signaling. It is not possible to assess if cytokine signaling was isolated to CARs but this data presumably indicates greater response to cytokines by all T-cells in response to cytokine release by antigen-activated CARs.

### APRIL CAR does not cause autoactivation

**Figure 4:**
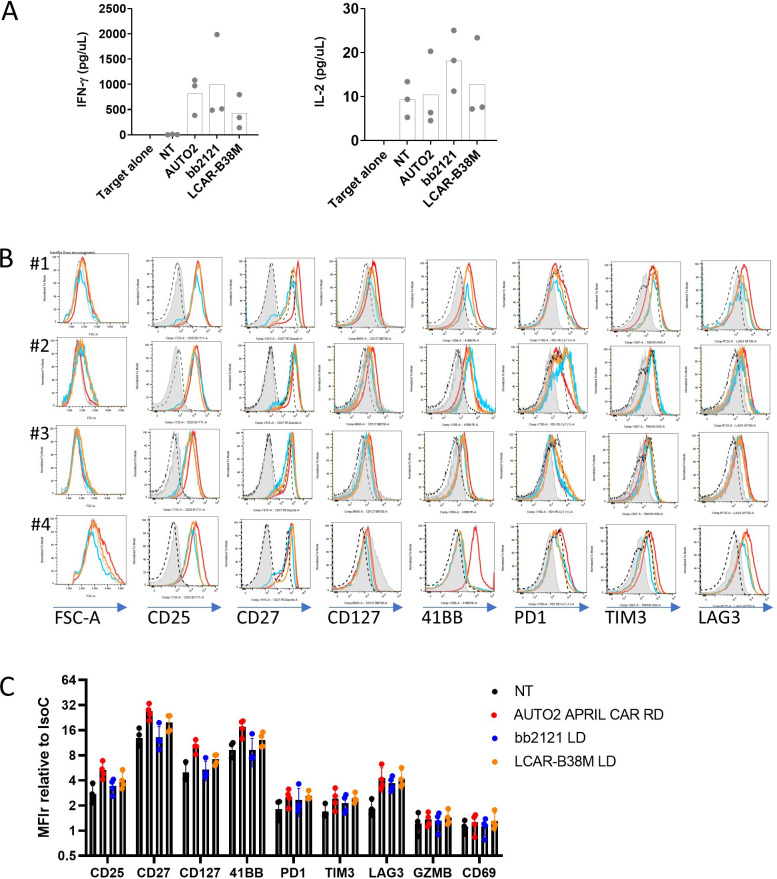
Assessment of autoactivation of AUTO2, bb2121 and LCAR-B38M. (A) Cytokine release from non-transduced (NT) PBMNCs or PBMNCs transduced with CAR constructs (retrovirus/RD transduced APRIL CAR, or LD/lentivirus transduced bb2121 or LCAR-B38M, four donors) and then co-cultured alone. IFN-γ and IL-2 from supernatant was quantified by ELISA. (B) Cells were then phenotyped by FACS at baseline (gray filled histograms) and after 7 days following initial activation and transduction with CAR constructs (AUTO2 in red, bb2121 in blue and LCAR-B38M in orange). Isotype control depicted in hatched black line. (C) Graph showing MFI of labeled proteins relative to isotype control. For each marker there was no significant difference between protein expression between AUTO2 and bb2121 or LCAR-B38M by multiple paired t-tests and Holm-Sidak correction. CAR, chimeric antigen receptor; FACS, Fluorescence activated cell sorting; GZMB, Granzyme B; IFN, interferon; IL, interleukin; LAG3: Lymphocyte Activation Gene 3; MFI: Mean Fluorescence Intensity; PBMNCs, peripheral blood mononuclear cells; PD-1, Programmed cell death protein 1; TIM3, T-cell immunoglobulin and mucin-domain containing-3.

APRIL naturally trimerizes^21^ and it has been shown previously that membrane-bound APRIL are present in oligomeric forms.^22^ We sought to look for evidence of autoactivation of the APRIL CAR but found no evidence for target independent CAR activation compared with bb2121 and LCAR-B38M. There was no significant increase in cytokine release or phenotypic markers of activation (CD35, CD27, CD127, 41BB, PD-1, TIM3, Lymphocyte Activation Gene 3 or LAG3) in CAR expressing T-cells cultured in the absence of target antigen for 7 days (figure 4). Furthermore, autoactivation was also not seen in the APRIL CAR in varying formats (summarized in figure 2A) varying in co-stimulation domains or γ-retroviral versus lentiviral transduction (online supplemental figure 8).

### Binding characteristics of APRIL is similar to other BCMA binders but there is deficient target binding by CAR expressing T-cells

We then looked for differences in tumor binding by APRIL. By surface plasmon resonance, we noted similar binding affinities of APRIL compared with BCMA binders used in bb2121 and LCAR-B38M (figure 5A). We then assessed protein stability of BCMA binders used in the APRIL, bb2121 (C11D5.3), LCAR-B38M (VHH binders in series or alone) CARs and found thermal unfolding consistently above 50°C indicative of stable proteins (figure 5B).

We hypothesized that despite the stability of recombinant APRIL, APRIL CAR may be less stable or suboptimally presented when expressed on the membrane surface. T-cells were transduced with constructs coexpressing RQR8 with bb2121, LCAR-B38M and APRIL CAR. T-cells were then stained with increasing concentrations of labeled soluble recombinant BCMA and analyzed by flow cytometry without removing unbound BCMA so that cell fluorescence would assess relative availability of surface CAR, unconfounded by difference in binding kinetics. APRIL CAR consistently bound less soluble ligand when incubated with various concentrations of fluorophore labeled BCMA compared with bb2121 and LCAR-B38M (figure 5C, online supplemental figure 9).

Appreciating the limitations of these platforms to assess the complex interaction between cells, we next sought to quantify the collective interactions of multiple receptor/ligand complexes and co-receptors which make up the immunological synapse.^23^ Using a platform of acoustic force technology to quantify avidity between effector and target cells (z-Movi), by demonstrated reduced avidity between APRIL CAR expressing cells and the human myeloma cell line H929 (figure 5D) compared with bb2121 and LCAR-B38M. Related to this, there was a trend for reduced phosphorylation of ZAP70 and LAT by phosphoflow supporting reduced TCR activation of the APRIL CAR in comparison to LCAR-B38M and bb2121 (online supplemental figure 10).

Thus despite the similar affinity of the APRIL protein to BCMA and the stability of the protein, we demonstrate deficient target binding by cell expressed APRIL as the cause of deficient CAR activation.

## Discussion

In 2017, we described a CAR based on the ligand APRIL for treatment of MM. Our motivation was to overcome what we anticipated would be limitations of scFv-based BCMA CARs: namely low antigen density of BCMA resulting in suboptimal signaling, and tumor escape through loss of BCMA. Rather than using an antibody-based binder, we used the natural ligand APRIL. Since APRIL CAR could recognize both BCMA and the related PC lineage antigen TACI, the total targetable antigen density was increased, and co-targeting two antigens should prevent single antigen negative escape. Initial functional tests of the APRIL CAR demonstrated antigen directed activation in vitro and in vivo, kill of low antigen expressing targets and efficacy against primary myeloma cells.^5^

We evaluated autologous APRIL CAR T-cells in patients with relapsed or refractory MM in a Phase 1, dose-escalation study (AUTO2). Eleven patients were treated, with median 5 prior lines therapy, 27% were of ISS III and 36% had high-risk cytogenetics. While the APRIL CAR was well tolerated, responses were only observed in 45.5% of patients and to a best response of a VGPR. In contrast to these results, two Phase 2 studies have become the reference points for BCMA CARs testing ide-cel and cilta-cel.^13 14^ A total of over 200 patients were treated in these studies and were more heavily pretreated with a median of 6 prior lines. While the frequency of severe CRS and ICANS was also low, the overall response rate, rate of CR and PFS was 73%, 33% and 8.8 months with ide-cel^14^ and 98%, 67% and not reached at 27.7 months with cilta-cel.^13 24^

Suboptimal APRIL CAR activity was also evidenced by low cytokine release and poor CAR expansion in patients. In AUTO2, increases in IFN-γ were only observed in four patients (range in trial of 0–2984 pg/mL) and peak IL-6 reached a median of 63 pg/mL (0–413) in the AUTO2 cohort. In comparison, in an early study with cilta-cel, increases in IL-6 were seen in most patients (13/17), reaching nearly 1×10^5^ pg/mL.^25^ An early study by Brudno *et al* describing a BCMA CAR with a CD28ζ endodomain described median fold change of >100 baseline of both IFN-γ and IL-6.^26^ Further, circulating APRIL CAR reached a median Tmax of 8673.7 copies/µg DNA (range 26–96,598) and was detectable for a median of 21 days (max 3 months). In comparison, CARs peaked and were detectable for a median of over 1×10^5^ copies/µg of DNA (max 5×10^6^) and a median of 6 months with ide-cel^27^ and a mean of over 1×10^4^ copies/µg (max 2×10^6^) for a median 4 months with cilta-cel,^13 25^ respectively.

APRIL CAR T-cells were manufactured using unmanipulated autologous cryopreserved leukapheresis as starting material, transduction with γ-retroviral vector and expansion in IL-7/IL-15 for 7–10 days. In comparison, manufacture of ide-cel^27^ and cilta-cel^25^ use lentiviral transduction and do not include IL-7/IL-15 which is thought to optimize memory phenotype in the manufactured CAR product.^28^ Looking for a cause for our poor clinical responses, we observed a median TE of 23% (range 5.81–40.2%) and a low proportion of naive and CM phenotypes (median combined percentage 18% of CARs). However, low TEs were also described with an early study of cilta-cel (median 22%),^25^ without impacting efficacy. From CD19 CAR T-cell studies, the proportion of naïve memory phenotypes has been shown to correlate with longer CAR persistence and improved patient outcomes.^29^ In their trial of BCMA-targeting CD28ζ CAR, Cohen *et al* describe between 20% and 30% of naive and stem cell memory T-cell populations^9^ and this figure was under 10% with ide-cel.^30^ Looking for a further reason for the low clinical responses, we also noted that BCMA expression was not a criteria for trial entry in the AUTO2 trial in common to other BCMA CAR trials.^13 24^ Thus the high proportion of mature memory phenotypes, low TEs observed and no requirement for tumor BCMA expression for trial entry were not unique to AUTO2 and could not sufficiently explain the low response rates in the AUTO2 trial.

We previously described antigen specific cytotoxicity in vitro by the APRIL CAR of targets expressing physiological levels of antigen and primary cells as well as antigen dependent IFN-γ release. We also demonstrated efficient in vivo clearance of tumor in a xenograft model. Given the effectiveness of the APRIL CAR in this murine model, we sought to explain the low rate of clinical responses observed in AUTO2 using a series of in vitro experiments, both making direct comparison to other BCMA CARs, bb2121 and LCAR-B38M, and also seeking more experimental readouts than attempted before. In this study, we demonstrate similar kill and IFN-γ secretion compared with bb2121 and LCAR-B38M. However, we also observed little or no IL-2 release by the APRIL CAR in response to low-density antigen expressing targets and reduced capacity for serial killing on repeated stimulation. These assays are likely to reflect the efficiency of CAR activation by target. Deficiencies in IL-2 secretion have been described following suboptimal T-cell activation by reduced recruitment of downstream proteins involved in TCR signaling such as LAT^31^ and Lck.^32^ Suboptimal T-cell signaling is associated with T-cell anergy^33^ which may in part explain reduced capacity for persistent tumor control. Additionally, deficiencies in cytokine signaling was a prominent finding on transcriptomic analysis of APRIL CAR compared with bb2121 and LCAR-B38M where we observed significantly reduced transcripts of genes involved in type 1 (eg, IRF7, MX1, OAS genes) and type 2 IFN (eg, CXCL10) signaling in stimulated APRIL CAR transduced T-cells. It is also noteworthy that APRIL CAR IL-2 secretion increased with TE. Thus, the low TE achieved in the AUTO2 trial may have exacerbated deficiencies in cytokine production as well as T-cell signaling.

These described functional deficiencies remained despite controlling for vector and endodomain, therefore, these deficiencies were likely due to use of APRIL as an antigen recognition domain. APRIL naturally trimerizes and may form higher-order concatemers.^22^ However functional testing revealed no increase in tonic signaling compared with bb2121 or LCAR-B38M. Next, we compared binding kinetics and protein stability of antigen recognition domains and found APRIL had similar binding affinity to BCMA compared with the antibody binders used in bb2121 and LCAR-B38M. Protein stability of the binders for the three CAR constructs was also observed at physiological temperatures. However, APRIL CAR expressing T-cells consistently bound less soluble BCMA than bb2121 or LCAR-B38M expressing T-cells.

A significant limitation of commonly used techniques for affinity readouts is that these do not accurately ascertain the complex interaction which exist between cells. In contrast, assessment of avidity accounts for the collective interactions of multiple receptor/ligand complexes and co-receptors which make up the immunological synapse between cells and can thus lead to more accurate predictions of T-cell functionality.^23^ We confirmed deficient target binding by APRIL CAR and reduced avidity between APRIL CAR expressing cells and the human myeloma cell line H929 compared with bb2121 and LCAR-B38M. Collectively, this data suggests that despite equivalent affinity of APRIL and BCMA at a protein level by Biacore, there is suboptimal target binding by cell expressed APRIL. This, in turn, indicates suboptimal or unstable presentation of APRIL in a CAR format, thereby resulting in poor APRIL CAR function and limited clinical responses observed in the AUTO2 trial.

APRIL also binds TACI, a tumor necrosis factor receptor that has a role in the maturation of B cells, with increased expression on maturing B-cell stages compared with BCMA.^34 35^ TACI was initially thought to be a T-cell antigen^36^ but now accepted to be expressed primarily on B cells^21^ with a recent report suggesting expression in suppressive T-cells.^37^ Importantly, TACI is expressed on MM cells.^5^ We found BCMA and TACI to be coexpressed on tumor for the majority (78%) of patients, but at generally lower levels than BCMA (median BCMA 1061 ABC, median TACI 333 ABC). AUTO2 is the first study which directly targets TACI; with the caveat of poor expansion and persistence, we did not find any toxicity attributable to TACI targeting.

Several CARs based on natural ligands have been described including some which have been tested in human subjects.^38 39^ Our data focuses exclusively on the APRIL CAR, however, our experience of the AUTO2 trial and subsequent comparisons to two other antibody-based BCMA CAR constructs do suggest that the physiological requirements of a natural ligand interaction may well differ from those optimal for CAR activation. Engineering strategies can be employed to influence the specificity or binding dynamics of natural ligands. For example, a single amino acid mutation has enhanced selective binding of IL-13 CARs to IL-13Rα2,^38 40^ we truncated the terminal amino acid of APRIL to minimize proteoglycan binding^5^ and more recently the enforced trimerization of APRIL has been demonstrated to improve target binding and CAR activity.^22^ Our data may indicate that improving the stability of the APRIL binder in a CAR format may improve function of the APRIL CAR.

It is noteworthy that with the increased use of BCMA CARs in patients, BCMA loss has been shown to be less frequent than initially anticipated^9 10^ indicating that potential benefit from dual targeting CARs will be primarily dependent on improved tumor targeting rather than preventing antigen negative escape. Thus, despite elegant engineering solutions available to optimize ligand based CARs, ligands are frequently limited to a single and possibly suboptimal presentation on a T-cell and may not meet the complex requirements for dual antigen binders. In comparison, human scFv or single-domain antibodies are now readily available and provide a choice of multiple potential binders that are amenable to manipulation for iterative optimization to meet specific clinical requirements. In the future, CAR T discovery may thus be better served using antibody derived antigen binding domains.

In summary, clinical testing of an APRIL based CAR proved disappointing compared with other studies testing antibody-based CARs. Our experience suggests that in comparison to the versatility and well understood stability of antibody derived binders, natural ligands may not be ideal antigen binding domains for CARs. Simple in vitro experimentation, may be informative as regards clinical performance. Namely, direct comparison to CAR constructs with proven clinical efficacy if available, use of targets expressing physiological levels of antigen, assessment of IL-2 release and prolonged tumor control on repeated stimulation. Further, beyond an assessment of affinity, we also indicate the value to ascertaining interaction of cellular interactions including avidity assessment. Finally, this work represents the first attempts at co-targeting of TACI, although future development may be better achieved with BCMA/TACI bicistronic CAR cassettes.^41–43^

10.1136/jitc-2023-006699.supp3 Supplementary data

10.1136/jitc-2023-006699.supp4 Supplementary data

10.1136/jitc-2023-006699.supp5 Supplementary data

## Data Availability

Data are available upon reasonable request. All data relevant to the study are included in the article or uploaded as supplementary information.
