# Correction: Limited efficacy of APRIL CAR in patients with multiple myeloma indicate challenges in the use of natural ligands for CAR T-cell therapy

**DOI:** 10.1136/jitc-2023-006699corr1

**Published:** 2023-07-11

Lee L, Lim WC, Galas-Filipowicz D, *et al*. Limited efficacy of APRIL CAR in patients with multiple myeloma indicate challenges in the use of natural ligands for CAR T-cell therapy. *J ImmunoTher Cancer* 2023; **11:** e006699. doi: 10.1136/jitc-2023-006699

This article has been corrected since it was first published. Author name Kwee Yong has been corrected.

