# Supplementary material for: Limited efficacy of APRIL CAR in patients with multiple myeloma indicate challenges in the use of natural ligands for CAR T-cell therapy
Source: J Immunother Cancer. 2023 Jun 30;11(6):e006699. doi: 10.1136/jitc-2023-006699 (PMC10314698; doi:10.1136/jitc-2023-006699)
Supplement: Supplementary data [file jitc-2023-006699supp001.pdf]

Limited efficacy of APRIL CAR in patients with multiple myeloma indicate challenges in the use of natural ligands for CAR T cell therapy

Lee et al

Supplementary Methods

Dose escalation in the AUTO2 study initially followed an accelerated dose titration design, in which a single patient was dosed at 15x10^6 CAR T cells, followed by further CAR doses (75, 225, 600 and 900x10^6 CAR T cells) in a 3+3 escalation design. Patients were administered lymphodepletion with fludarabine (30 mg per square meter of body-surface area per day) and cyclophosphamide (300 mg per square meter per day) on days –6, –5, and –4, followed by an infusion of APRIL CAR on day 0). The full protocol is available with the full text of this article

Dose-Limiting Toxicities

The DLT evaluation period was 28 days after the AUTO2 dose

Dose limiting toxicity was defined as:

1. Any new non-haematological AE of Grade 3 or higher toxicity using the NCI CTCAE (Version 4.03), which is probably or definitely related to AUTO2 therapy, which occurs within the DLT evaluation period, and which fails to resolve to Grade 2 or better within 14 days, despite appropriate supportive measures.
2. A Grade 4 CRS. (Lee et al. 2014)
3. Any other reason for activation of the safety switch after receiving AUTO2.
4. Any other fatal event (Grade 5) or life-threatening event (Grade 4) that cannot be managed with conventional supportive measures or which in the opinion of the SEC necessitates dose reduction or other modification to trial treatment to avoid a similar hazard in future patients. Effort should be made to perform an autopsy in case of fatal event where the aetiology is unclear.
5. Any event that in the opinion of treating investigators and/or Medical Monitor puts the patient at undue risk may also be considered a DLT.

Cellular Kinetics Assay

Copies of vector transgene per micrograms genomic DNA was determined by quantitative PCR (qPCR)

Characterization of the CAR Product (Facs panels)

Vials of manufactured CAR were defrosted and stained with the following antibodies before analysis with a Fortessa(BD) and Flowjo (V10.6). BUV395 HLA-DR (G46-6,BD), BUV496 CD8 (RPA-T8, BD), BUV737 CD27 (MT271, BD), BUV805 CD4 (SK3, BD), BV421 CXCR3 (1C6, BD), BV480 CD45RA (HI100, BD), BV605 CD3 (SK7, BD), BV650 CD62L (DREG-56, BD), BV711 TIM3 (7D3, BD), BV786 CD25 (M-A251, BD), FITC LAG3 (11C3C65, BD), PerCP efluor710 Tigit (MBSA43 Ebio), PE CD34 (RQR8, R&D), PE-Dazzle CCR7 (GO43H7, Biolegend), PE-Cy7 PD1 (EH12.1, BD), APC CD2 (RPA-2.10, Biolegend), AF700 GranzymeB (GB11, BD), efluor780 fixable viability dye (eBioscience).

Analysis of bone marrow trephines

Formalin-fixed paraffin-embedded tissue was stained for BCMA and TACI and RQR8 as previously described[1–3].

Analysis of bone marrow aspirates

BM mononuclear cells (MNC) were isolated by Ficoll Paque (GE Healthcare) centrifugation and cryopreserved in FBS (Gibco) containing 10% DMSO (Sigma-Aldrich). Aliquots were subsequently thawed for antibody staining and flow cytometry using two separate panels. Cells were run by BD symphony and data analysed by Flowjo (V10.6).

Lymphoid panel. BMMNCs were stained for the following extracellular markers BUV395 CD3 (SK7, BD), BUV496 CD4 (SK3, BD), BUV563 CD45RA (HI100, BD), BUV661 CD38 (HIT2, BD), BUV737 ICOS (DX29, BD), BV500 CD8 (RPA-T8, BD), BV605 CD56 (NCAM16.2, BD), BV711 TIM3 (7D3, BD), BV786 CCR7 (G04 3H7, Biolegend), BB515 CD25 (2A3, BD), PE CD34 (QBend10, R&D), PE/Dazzle LAG3 (11C3C65, Biolegend), PE-Cy7 PD1 (EH12.1, BD), efluor780 fixable viability dye (eBioscience). Cells were then permeabilized with Invitrogen Fixation/Permeabilization kit before intracellular staining with BV421 CTLA4 (BNI3, BD), BV650 Ki67 (B56, BD), AF647 FoxP3 (259D, Biolegend), AF700 GranzymeB (GB11, BD).

Myeloid panel. BMMNCs were stained for the following extracellular markers BUV395 HLA-DR (G46-6, BD), BUV496 CD38 (HIT2, BD), BUV563 CD19 (SJ25C1, BD), BUV737 CD14 (M5E2, BD), BUV805 CD45 (HI30, BD), BV421 CD163 (GHI/61, BD), BV510 CD11b (ICRF44, BD), BV605 CD3 (SK7, BD), BV605 CD56 (NCAM16.2, BD), BV650 CD15 (HI98, BD), BV711 CD33 (WM53, BD), BV786 CD303 (201A, Biolegend), BB515 CD1c (F10/21A3, BD), BB515 CD141 (1A4, BD), PerCP-Cy5.5 CD206 (15-2, Biolegend), PE BCMA (19F2, Biolegend), PE-CF594 PDL-1 (MIH1, BD), APC-R700 CD138 (MI15, BD), efluor780 fixable viability dye (eBioscience). Cells were then permeabilized with BD cytofix/cytoperm before intracellular staining with PE-Cy7 CD68 (Y1/82A, BD).

In vitro Coculture methods

CAR T cells for in vitro functional assessment were generated by transduction of healthy donor peripheral blood mononuclear cells (PBMC) obtained by density gradient centrifugation (Ficoll Paque, GE Lifesciences) with retroviral or lentiviral vectors. Retroviral supernatants were generated by co-transfection of 293T with RD114, PeqPam-env as well as an SFG plasmid encoding the APRIL CAR downstream of an RQR8 marker and 2A autocatalytic site. Calls were stimulated with CD3 and CD28 antibodies (0.5 µg/mL; Miltenyl) and interleukin-2 (100 IU/mL; Genescript) then transduced as before[32] to obtain CAR T cells. Lentiviral supernatants were generated by co-transfection of 293T packaging cells with third-generation lentiviral packaging plasmids pMD2.G, pMDLg/RRE, pRSV as well as the pCCL.APRILCAR transfer vectors using GeneJuice transfection reagent. Ficoll-isolated healthy donor PBMCs were transduced following overnight activation with Transact, IL15 and IL7 (Miltenyi). Transduction efficiency was determined by staining of cells with BCMA-hFc protein (R&D).

MM1s and 293T cell lines were obtained from the American Type Culture Collection. SupT1 cells were purchased from the European Collection of Authenticated Cell Cultures. Cytokines were assayed using Biolegend ELISA kits.

Surface plasmon resonance

LCAR-B38M VHH antibody sequences were obtained from patent US20210128618A1, linked with a $(G_4S)_3$ linker, and expressed as murine IgG2a Fc fusion via transient transfection on ExpiCHO cell line (Gibco). C11D5.3 scFv sequence was obtained from WO2016094304A2 and expressed as murine IgG2a Fc fusion via transient transfection on ExpiCHO cell line (Gibco). Antibodies were purified via Protein A affinity chromatography. Recombinant APRIL (Q6U6I7 aa 12-146) was expressed as triple N-term FLAG tagged fusion protein with C-Terminal human IgG1 hinge domain and 8xHis. Protein was expressed via transient transfection on Expi293 cell line (Gibco) and purified via TALON (Takara) metal affinity chromatography.

For kinetic measurements, antibodies were captured on a Series S protein A sensor chip (Cytiva) between 230-290 response units (RU) using a Biacore T200 instrument (Cytiva). Recombinant BCMA (Acro Biosystems), dialysed in HBSP+ buffer (Cytiva), at known concentrations was flown over the flow cells at 30 µl/min for 150s with 300s of dissociation. Recombinant APRIL was directly immobilised on the surface of a Series S CM5 sensor chip (Cytiva) at 800-1000 RU on a Biacore 8k instrument (Cytiva). Recombinant BCMA (Acro Biosystems), dialysed in HBSP+ buffer (Cytiva), at known concentrations was flown over the flow cells at 30 µl/min for 150s with 300s of dissociation, using a parallel kinetic setup. HBSP+ buffer was used as running buffer in all experimental conditions. An empty flow cell was used as reference subtraction and a double buffer injection was used as blank. Sensograms were fitted with a 1:1 Langmuir binding model using local Rmax calculations.

Differential Scanning Fluorometry (NanoDSF) assay for protein stability

Differential scanning fluorimetry was performed on a Prometheus NT.48 instrument (Nanotemper) using glass capillaries. Approximately 10 µl of antibody solution in PBS was subjected to a temperature ramp of 1 °C/min from 20 to 95 °C. Melting temperature (Tm) was measured as first derivative of 350/330nm absorbance ratio.

CAR activation and RNA seq

1ml of Fc conjugated BCMA (Peprotech) at a concentration of 400ug/ml was incubated per well in a 24 well plate. Half a million RD APRIL CAR , LD bb2121 and LD LCAR-B38M transduced T cells from three healthy donors were incubated in a well for 24 hours prior to harvest. RNA was then extracted (Qiagen RNAeasy kit) prior to bulk RNAseq (Novogene).

The output reads were quantified using salmon against a customised transcriptome by combining the three CAR gene cDNA sequences and transcriptome from GENCODE (Release 41) 1,2. Differential gene expression analysis was performed using R package

DESeq2 (R version 4.2.1, DESeq2 version 1.36.0)3,4. Volcano plot was made using ggplot2 in R (ggplot2 version 3.3.6)5.

GO Seq enrichment analysis was performed using R package goseq (R version 4.2.1, goseq version 1.48.0)3,6. Differentially expressed genes were defined as genes with adjusted p value ≤ 0.05 and an absolute fold change ≥ 1. Gene sets representing Reactome pathway were built using the annotation on BioMart (Human genes (GRCh38.p13)) using R (R version 4.2.1, biomaRt version 2.52.0)7-9. Dot plots were made using ggplot2 in R (ggplot2 version 3.3.6)5.

Avidity Measurements

H929 cells were seeded on poly-L-lysine coated z-Movi® microfluidic chips at a density of 100x10^6 cells/mL. Cells were then incubated for 0.5 hour in serum free media followed by 2-hour incubation in complete media. Effector cells were stained with Cell Trace far-red (ThermoFisher Scientific, C34564) at a dilution of 1:1000. Labelled effector cells were introduced on to the chip at a density of 1x10^6 and incubated for 10 minutes prior to acoustic force application using z-Movi ® cell avidity analyzer. Detachment of effector cells were analyzed using Oceon 1.4 software. Experiments were performed with 4 donors CAR T cells and untransduced control on each chip.

Phosphoflow analyses

1x10^5 H929 cells were added to 1x10^5 untransduced or CAR-transduced T cell, both preincubated for 20 minutes with fixable viability dye (ebioscience), before brief centrifugation to pellet the cells, incubated for 5 mins before fixation with 1.5% paraformaldehyde and PhosSTOP (Roche) for 15 minutes. Cells were the permeabalised with cold 90% Methanol for 15 minutes before staining with anti-ZAP70Phos tyr319 AF 488, anti- LATphospho Tyr 171 AF647, anti CD138-AF700, anti CD34-FITC or APC(RnD), (all antibodies Biolegend unless stated otherwise) and analysis on the NovoCyte Quanteon Flow Cytometer.

References

1. Patro, R., Duggal, G., Love, M. I., Irizarry, R. A., & Kingsford, C. (2017). Salmon provides fast and bias-aware quantification of transcript expression. Nature Methods, 14, 417–419. DOI: 10.1038/nmeth.4197

2. Frankish, A., Diekhans, M., Jungreis, I., Lagarde, J., Loveland, J.E., Mudge, J.M., Sisu, C., Wright, J.C., Armstrong, J., Barnes, I., Berry, A., Bignell, A., Boix, C., Carbonell Sala, S., Cunningham, F., Di Domenico, T., Donaldson, S., Fiddes, I.T., García Girón, C., Gonzalez, J.M., Grego, T., Hardy, M., Hourlier, T., Howe, K.L., Hunt, T., Izuogu, O.G., Johnson, R., Martin, F.J., Martínez, L., Mohanan, S., Muir, P., Navarro, F.C.P., Parker, A., Pei, B., Pozo, F., Riera, F.C., Ruffier, M., Schmitt, B.M., Stapleton, E., Suner, M.M., Sycheva, I., Uszczynska-Ratajczak, B., Wolf, M.Y., Xu, J., Yang, Y.T., Yates, A., Zerbino, D., Zhang, Y., Choudhary, J.S., Gerstein, M., Guigó, R., Hubbard, T.J.P., Kellis, M., Paten, B., Tress, M.L., & Flicek, P. (2021). GENCODE 2021. Nucleic Acids Res, 49(D1), D916-D923. DOI: 10.1093/nar/gkaa1087

3. R Core Team (2022). R: A language and environment for statistical computing. R Foundation for Statistical Computing, Vienna, Austria. Retrieved from https://www.R-project.org/

4. Love, M.I., Huber, W., Anders, S. (2014). Moderated estimation of fold change and dispersion for RNA-seq data with DESeq2. Genome Biology, 15, 550. DOI: 10.1186/s13059-014-0550-8.

5. Wickham, H. (2016) ggplot2: Elegant Graphics for Data Analysis. Springer-Verlag New York.

6. Young, M.D., Wakefield, M.J., Smyth, G.K., Oshlack, A. (2010). Gene ontology analysis for RNA-seq: accounting for selection bias. Genome Biology, 11, R14. DOI: 10.1186/gb-2010-11-2-r14

7. Gillespie, M., Jassal, B., Stephan, R., Milacic, M., Rothfels, K., Senff-Ribeiro, A., Griss, J., Sevilla, C., Matthews, L., Gong, C., Deng, C., Varusai, T., Ragueneau, E., Haider, Y., May, B., Shamovsky, V., Weiser, J., Brunson, T., Sanati, N., . . . D'Eustachio, P. (2021, November 12). The reactome pathway knowledgebase 2022. Nucleic Acids Research, 50(D1), D687–D692. DOI: 10.1093/nar/gkab1028

8. Durinck, S., Moreau, Y., Kasprzyk, A., Davis, S., De Moor, B., Brazma, A., & Huber, W. (2005, August 4). BioMart and Bioconductor: a powerful link between biological databases and microarray data analysis. Bioinformatics, 21(16), 3439–3440. DOI: 10.1093/bioinformatics/bti525

9. Durinck, S., Spellman, P. T., Birney, E., & Huber, W. (2009, July 23). Mapping identifiers for the integration of genomic datasets with the R/Bioconductor package biomaRt. Nature Protocols, 4(8), 1184–1191. DOI: 10.1038/nprot.2009.
