# Supplementary material for: Limited efficacy of APRIL CAR in patients with multiple myeloma indicate challenges in the use of natural ligands for CAR T-cell therapy
Source: J Immunother Cancer. 2023 Jun 30;11(6):e006699. doi: 10.1136/jitc-2023-006699 (PMC10314698; doi:10.1136/jitc-2023-006699)
Supplement: Supplementary data [file jitc-2023-006699supp002.pdf]

Patient ID	ISS stage at presentation	Cytogenetics markers	Paraprotein or light chain type in serum	Presence of Extra-medullary disease	% Tumour by FACS (as a proportion of BM MNCs)	# of prior lines of therapy	Refractory to IMiD	Refractory to Proteasome Inhibitor	Refractory to IMiD and PI	Refractory to anti-CD38	Prior lines of therapy - Best response
0201GB01001	III	1q gain, t(11;14)	IgG	No	5	4	Yes	Yes	Yes	No	1) Dex/Thalidomide/Bortzomib - MR<br>2) Cisplatin/Cytarabine/Etoposide/Methylprednisolone - SD<br>3) Dex/Ixamib/Len - SD<br>4) Cisplatin/Cy/Dex/Etoposide/Thalidomide/Dox - SD
0201GB01002	I	t(11;14)	IgG	No	53	3	Yes	Yes	Yes	No	1) Dox/Bortezomib/Dex/Mephalan/Transplant - VGPR<br>2) Bortezomib/Dex/Thalidomide/Panobinostat - PD<br>3) Dex/Len/Ixazomib - PR
0201GB01003	II	normal	IgG	Yes	3.2	6	Yes	Yes	Yes	No	1) Cy/Dex/Thalidomide - UKN<br>2) Dex/Bortezomib - MR<br>3) Dex/Len - PD<br>4) Cy/Dex/Bortezomib - PR<br>5) Bendamustine/Dex/Thalidomide - PR<br>6) Clarithromycin/Dex/Len - SD
0201GB01005	I	UNK	IgG	No	0	6	Yes	Yes	Yes	No	1) CyDex/Dox/Vincristine - PR<br>2) Bortezomib/Dex/Dox - PD<br>3) Thalidomide - PR<br>4) Mephalan/transplant - UKN<br>5) Dex/Len - PR<br>6) Investigative drug (CC-220) - PD
0201GB01006	I	UNK	Light chain only	No	5	5	Yes	Yes	Yes	No	1) Cy/Dex/Thalidomide - PR<br>2) Dex/Bortezomib/Thalidomide - SD<br>3) Cisplatin/Cy/Dex/Dox/Etoposide/Thalidomide/Bortezomib - PR<br>4) Cy/Dex/Len - PR<br>5) Dex - SD
0201GB05007	UNK	UNK	IgG	No	2	5	Yes	No	No	Yes	1) Cy/Dex/Thalidomide - VGPR<br>2) Bortezomib/Dex - PR<br>3) Dex/Len/ - VGPR<br>4) Dex/Len - PR<br>5) Daratumumab - PD
0201GB05008	I	UNK	Light chain only	Yes	26	5	Yes	Yes	Yes	No	1) Cy/Dex/Thalidomide/Melphalan/Transplant - PR<br>2) Cy/Dex/Bortezomib/Mephalan/Transplant - PR<br>3) Cy/Dex/Len - SD<br>4) Dex/Pomalidomide - PD<br>5) Dex/Bortezomib/Panobinostat - PD
0201GB01009	III	UNK	IgG	Yes	0	6	Yes	Yes	Yes	Yes	1) Bortezomib/Dex/Mephalan/Transplant - CR<br>2) Dex/Len - UNK<br>3) Carfilzomib/Dex - UNK<br>4) Daratumumab - UNK<br>5) Dex/Ixamib/Pomalidomide - UNK<br>6) Daratumumab/Dex/Bortozomib - UNK
0201NL01010	I	1q+, Hyperdiploidy	IgG	No	0	4	Yes	Yes	Yes	Yes	1) Bortezomib/Cy/Dex/Mephalan/Transplant - VGPR<br>2) Carfilzomib/Dex/Len - MR<br>3) Daratumumab/Tretinoin - SD<br>4) Cy/Dex/Pomalidomide - PR
0201GB01011	I	1q+, Del17p13, t(11;14)	IgG	No	5	6	Yes	Yes	Yes	Yes	1) Cy/Dex/Len - VGPR<br>2) Carfilzomib/Cy/Dex - CR<br>3) Dex/Thalidomide/Bortezomib - VGPR<br>4) Dex/Panobinostat/Bortezomib - SD<br>5) Dex/Ixazomib/Len - PD<br>6) Dex/Pomalidomide/Daratumumab - PR
0201GB01012	III	1q+, Del17p13	IgG	No	11	4	Yes	No	No	Yes	1) Cy/Dex/Bortezomib/Melphalan/Transplant - PR<br>2) Dex/Len - PR<br>3) Investigational drug (CC-220)/Dex - PR<br>4) Daratumumab - SD

Supp Table 1. AUTO2 patient details. Unknown (UNK), lenalidomide (len), dexamethasone (dex), Doxorubicin (Dox), Cyclophosphamide (cy), Stable Disease (SD), Minor Response (MR), Partial Response (PR), Very Good Partial Response (VGPR), Complete Response (CR), Autologous Stem Cell Transplant (Transplant)

* Patient 005 had a history of pelvic radiotherapy thus bone marrow biopsies challenging

Patient ID	Bridging therapy and response	Dose received (10^6 APRIL CAR T positive T cells)	Transduction efficiencies (%)	Best response according to IMWG (after 1st treatment)	Retreatment Bridging therapy and response	Retreatment dose received (10^6 APRIL CAR T positive T cells)	Retreatent best response according to IMWG	Time of disease progression (from 1st treatment)	Survival Status (Study Day from 1st treatment)
0201GB01001	No bridging	15	32.4	SD	No bridging	225	SD	Not applicable	Alive (912)
0201GB01002	No bridging	75	5.81	SD	Not applicable	Not applicable	Not applicable	M3	Alive (890)
0201GB01003	No bridging	75	12.74	SD	Not applicable	Not applicable	Not applicable	M2	Dead (342)
0201GB01005	No bridging	75	8.9	PD	Daratumumab (SD)	250	PR	M1	Dead (425)
0201GB01006	No bridging	225	23	SD	Not applicable	Not applicable	Not applicable	M2	Dead (269)
0201GB05007	Dexamethasone/ Pomalodomide (PD)	200	8.7	PR	Not applicable	Not applicable	Not applicable	Discontinued at M6 while still in PR	Dead (373)
0201GB05008	Daratumumab (PD)	225	32	SD	Not applicable	Not applicable	Not applicable	M2	Dead (201)
0201GB01009	No bridging	600	17	VGPR	Not applicable	Not applicable	Not applicable	M8	Dead (375)
0201NL01010	Cyclophosphamide/ Dexamethasone/ Pomalodomide (SD)	600	40.2	PR	Not applicable	Not applicable	Not applicable	M4	Dead (266)
0201GB01011	No bridging	600	41	MR	Not applicable	Not applicable	Not applicable	M2	Dead (483)
0201GB01012	No bridging	900	24.2	SD	Not applicable	Not applicable	Not applicable	M3	Alive (333)

Supp Table 2. AUTO2 bridging and disease response

			FACS			IHC				
Patient	Dose	Timepoint	% CD138 /BMMNCs	BCMA (ABC)	TACI (ABC)	%CD138/ BMMNCs	% T cells/ BMMNCs	% CAR/ CD3+	BCMA (intensity, % of PCs)	TACI (intensity, % of PCs)
AUTO2-001	15x10^6	Baseline	5	548	1819	40-50	10	0	Moderate/strong, 50%	Weak/mod 40%
		1M	1	139	577	60-70	20	15	Weak, 50%	Weak/mod, 30-40%
		3M	1.6	293	629	30	10	1	Weak, 10%	Moderate, 40%
		6M	1.2	287	653	5	1 -2 %	0	Weak, 10%	Weak, 30%
	225x10^6	2nd Baseline	1.2	412	1147	0.4	0.01	0	Weak, 20%	Weak/mod, >50%
		1M	0.18	214	569	0.8	0.05	1%	Weak, >50%	Moderate, >50%
		6M	0.7	574	1824	supoptimal				
		12M	not avialable			50%	5%	<1%	Moderate, >50%	Moderate, >50%
AUTO2-002	75x10^6	Baseline	52.9	640	381	70	1-2%	0	Weak, 20	Moderate, 30-40%
		1M	22	771	563	60-70	10	1-2%	Moderate, 30-40%	Weak, 30-40%
		3M	43	403	173	80	5	2-3%	Moderate 40%	Moderate, 40%
AUTO2-003	75x10^6	Baseline	3.2	653	376	40-50	1-2%	0	Weak, 50%	Weak, 10-20%
		1M	1.8	683	271	50-60%	10%	5%	Weak 5%	Moderate 40%
AUTO2-005	75x10^6	Baseline	0	N/A	N/A	suboptimal	n/a	n/a	n/a	n/a
		1M	0	N/A	N/A	suboptimal	n/a	n/a	n/a	n/a
	225x10^6	Baseline	0.3	1084	246	20%	0	0	Strong, 100% pc	negative
		3M	1.3	2623	0	0	0	n/a	n/a	n/a
AUTO2-006	225x10^6	Baseline	4.6	596	579	80%	1%	0	Strong, >50%	moderate, 40%
		1M	21.7	235	258	25%	2%	1%	Strong, >50%	Weak, 10%
		3M	0.3	441	460	70-80%	0	0	Moderate, 30-40%	Weak, <5%
AUTO2-007	225x10^6	Baseline	2.4	441	267	10%	1-2%	0	Moderate, >50%	Weak, 10%
		1M	0.02	154	0	0	0	0	n/a	n/a
		3M	0.3	172	137	<1%	2%	1%	n/a	n/a
		6M	0.7	87	540	<1%	1%	0	n/a	n/a
AUTO2-008	225x10^6	Baseline	26	431	622	not available				
		1M	29	1409	1384	30-40%	5%	1%	Moderate, 30-40%	Weak, 50%
AUTO2-009	600x10^6	Baseline	0	N/A	N/A	0	<1%	0	n/a	n/a
		1M	0	N/A	N/A	0	<1%	0	n/a	n/a
		3M	0	N/A	N/A	0	5-10%	1%	n/a	n/a
		6M	0	N/A	N/A	0	2%	occasional	n/a	n/a
AUTO2-010		Baseline	0	N/A	N/A	0	2%	0	n/a	n/a
		1M	0	N/A	N/A	not available				
		3M	0.1	-	602					
AUTO2-011	600x10^6	Baseline	7.5/5.4	780	0	60-70%	2%	0	Weak, 50%	Negative
		1M	0.2	400	203	80%	1-2%	0	Weak, 50%	Negative
AUTO2-012	900x10^6	Baseline	11	516	549	90%	1%	0	Weak, >50%	Negative
		1M	0.54	782	486	90%	10%	2%	Moderate, >50%	Negative
		3M	12.5	322	309	90%	5%	1%	Moderate/strong, 50%	Negative

Supp Table 3. BCMA and TACI quantification. Months (M), Antigens Bound per Cell (ABC), Bone Marrow Mononuclear Cells (BMMNCs), Plasma Cells (PC)

Patient ID	CRS	CRS Grade	Neurotox	Received Tociluzimab	Other toxicity
0201GB01001	Yes	1	No		Anaemia G3<br>Neutropenia G4<br>Thrombocytopenia G4
0201GB01002	No	-	No		Anaemia G3<br>Neutropenia G4<br>Bone Pain G3<br>Arthralgia G3<br>Pneumonia G3<br>Sinusitis G3<br>Vomitting G3
0201GB01003	Yes	1	No	yes	Neutropenia G4<br>metapneumovirus G3<br>Breast Ca-G5
0201GB01005	No	-	No		Anaemia G3<br>Neutropenia G4<br>Enterovirus G3<br>Pneumonia G3<br>Rhinovirus G3<br>Headache G3
0201GB01006	No	-	No		Neutropenia G4<br>Bone pain G3
0201GB05007	No	-	No		Anaemia G3<br>Neutropenia G4<br>Pseudomonas bacteraemia G3
0201GB05008	No	-	No		Anaemia G3<br>Neutropenia G4
0201GB01009	Yes	1	No	Yes	Anaemia G3<br>Neutropenia G4<br>hypocalcaemia G3<br>hypophasphataemia G3
0201NL01010	Yes	1	No		Anaemia G3<br>Neutropenia G4<br>Thrombocytopenia G4
0201GB01011	No	-	No		Neutropenia G4<br>entercoccus faecalis UTI G3
0201GB01012	Yes	1	No	Yes	Anaemia G3<br>Neutropenia G4<br>Myocardial infarction G3

Supp Table 4. Grade 3 or higher toxicity observed in AUTO2

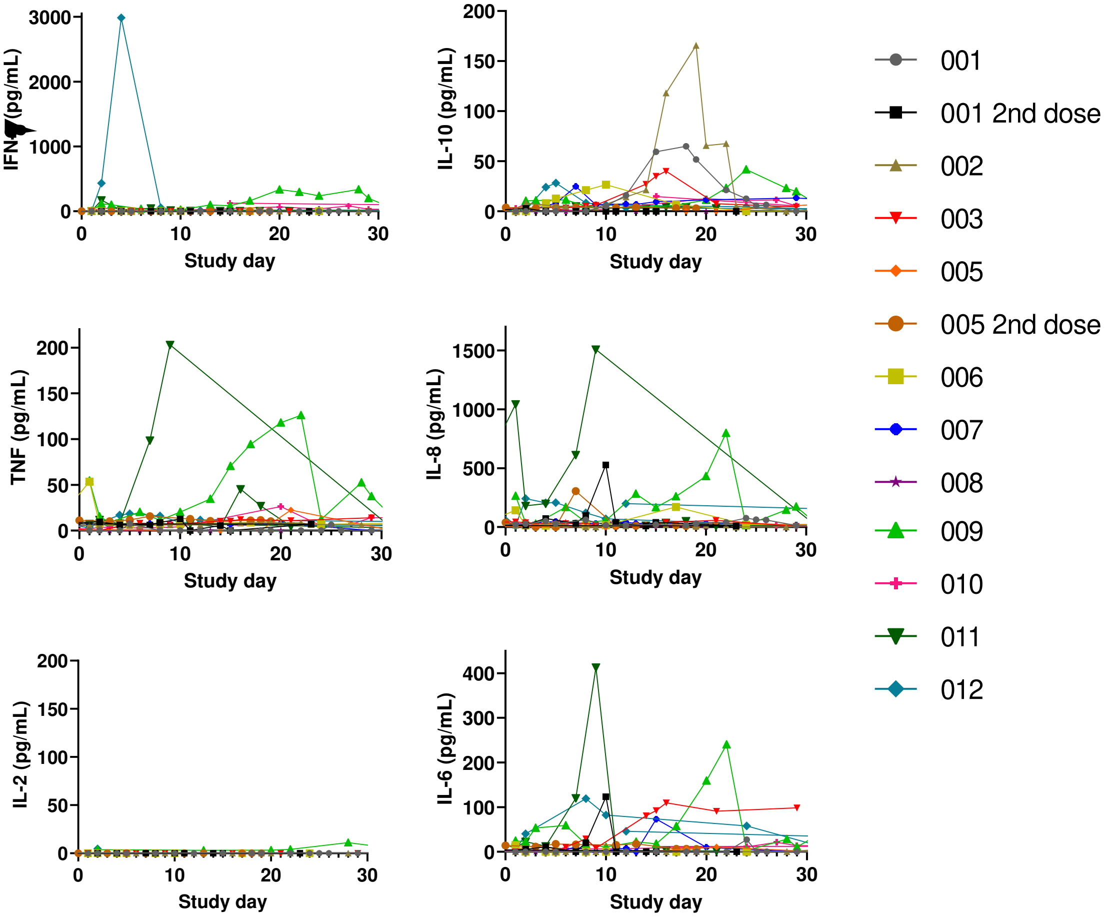

Supp Figure 1 Serum cytokines levels in the first month post CAR T-cell infusion.

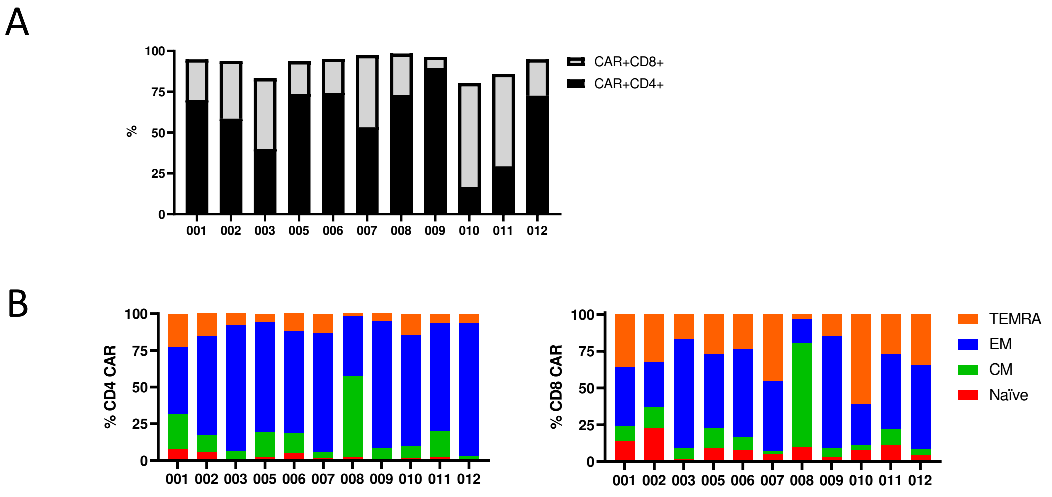

Supp Figure 2. (A) CD4:CD8 ratio and (B) Memory subsets of manufactured APRIL CAR product. Terminally differentiated effector memory cells re-expressing CD45RA (TEMRA), effector memory(EM), central memory(CM).

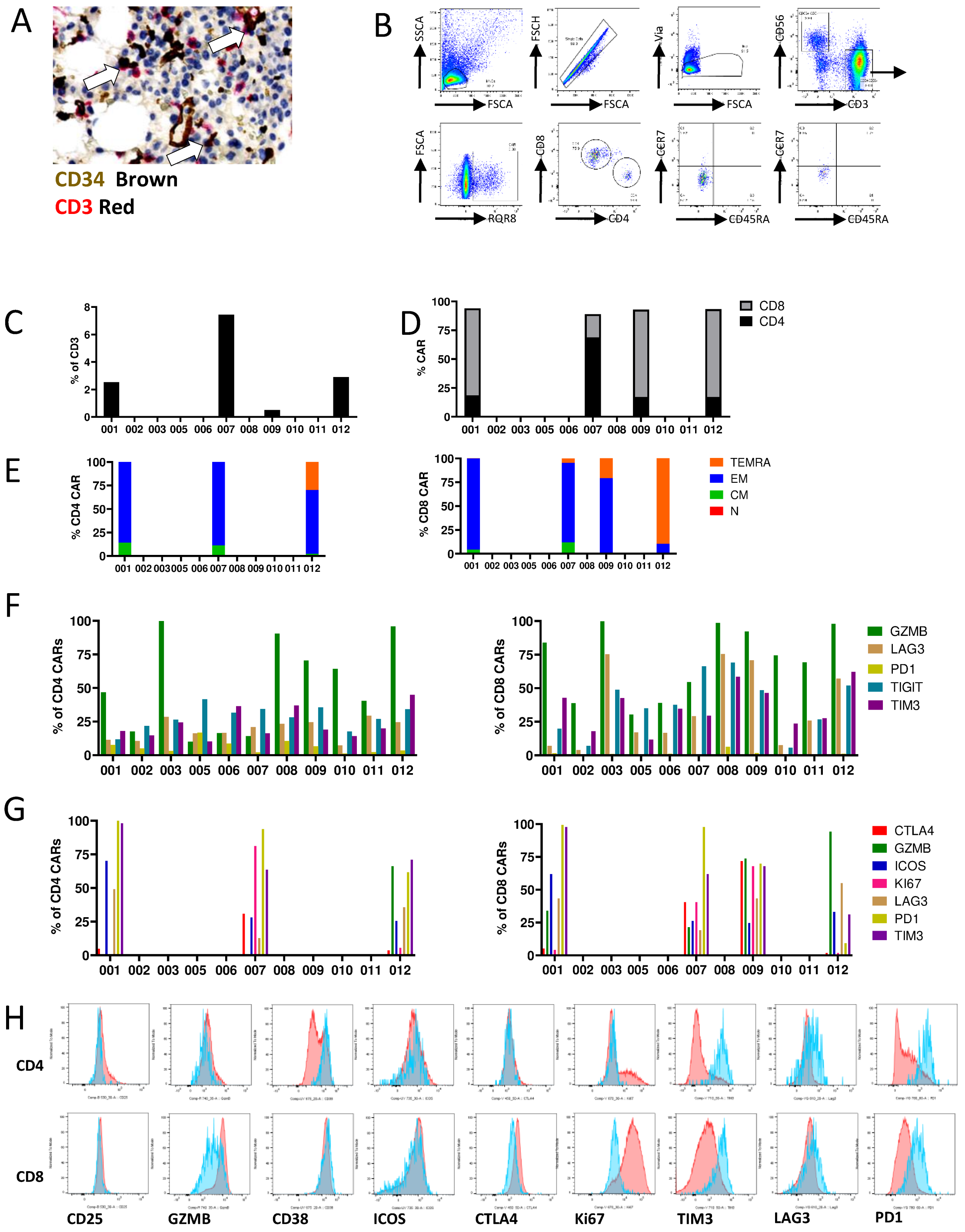

Supp Figure 3. (A)CAR T cells (arrowed) in the tumour niche were also observed by multiplex immunohistochemistry. (B) An example gating strategy for patient 001 shown. Results displayed if there were >50 CD4 or CD8 CAR T cells analysed. CAR T cells also expressed RQR8 marker containing the CD34 epitope recognised by QBend10 antibodies. BM aspirate from treated patients were analysed by FACS 1 month post infusion for (C) presence of CAR T cells (D) CD4:CD8 ratio and in samples with a minimum of 50 CAR T cells, memory phenotype (E) was analysed. CAR expression of immunomodulatory proteins on APRIL CAR T cell product (F) and at month 1 post infusion from bone marrow aspirate (G). (H)Comparison of expression of activation, proliferation or immune modulation markers on APRIL CAR T cells (blue) and non CAR T cells (red) from the BM of patient 001 1 month post infusion of first dose.

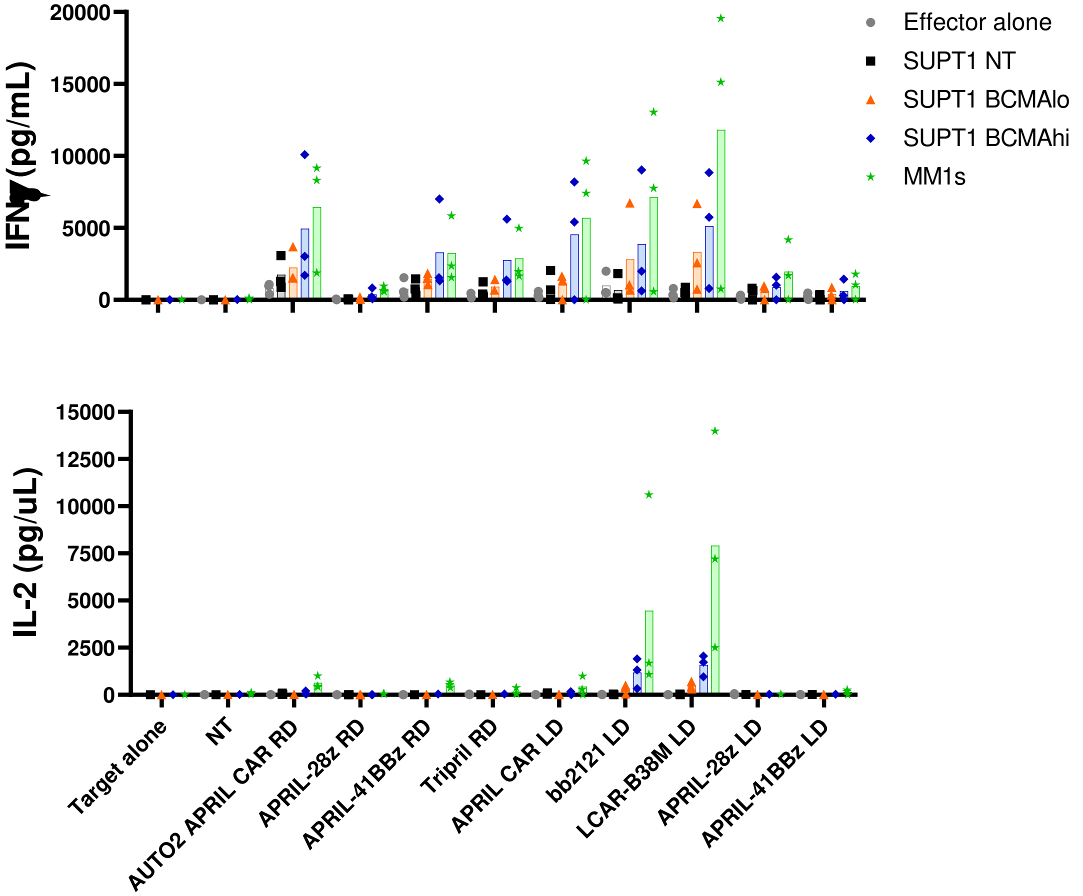

Supp Figure 4. Cytokine release by APRIL CAR variants, bb2121 and LCAR-B38M CAR in vitro. CAR transduced PBMNCS from normal donors (n=3) were cocultured with non transduced SUPT1 targets (SUPT1 NT) or targets engineered to express low levels of BCMA (SUPT1 BCMAlo, 636 molecules per cell), high levels of BCMA (SUPT1 BCMAhi, 2x10^5 molecules per cell) and MM1.s cells at an effector to target ratio (E:T) of 1:4. IFNγ and IL2 release as assessed by ELISA of culture supernatant at 24 hours.***=p<0.001. RD=retrovirus and LD= lentivirus

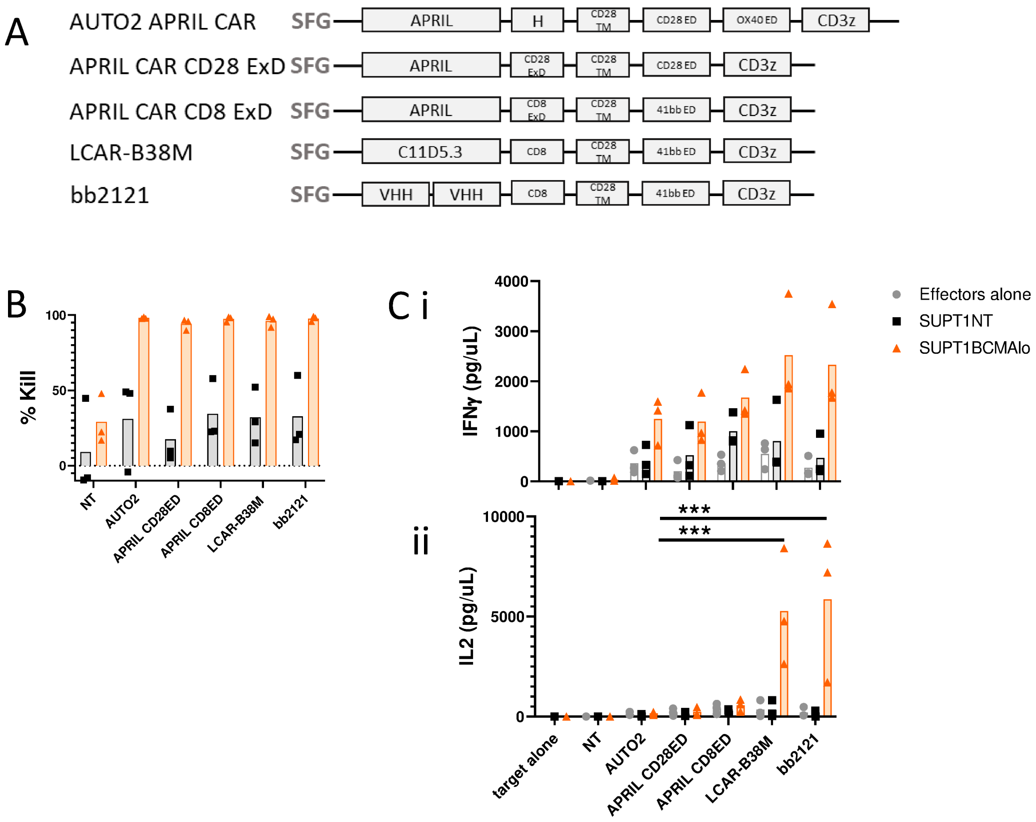

Supp Figure 5. Target kill and cytokine release by APRIL CAR with varying exodomains in vitro. (A) Diagram summarising various CAR constructs assessed functionally. CAR transduced PBMNCS from normal donors (n=3) were cocultured with non transduced SUPT1 targets (SUPT1 NT) or targets engineered to express low levels of BCMA (SUPT1 BCMAlo, 636 molecules per cell), at an effector to target ratio (E:T) of 1:4. **(B)** Target kill as a percentage of targets in media alone **(C)** IFNγ and IL2 release as assessed by ELISA of culture supernatant at 24 hours. ***= $p<0.001$ by paired T test.

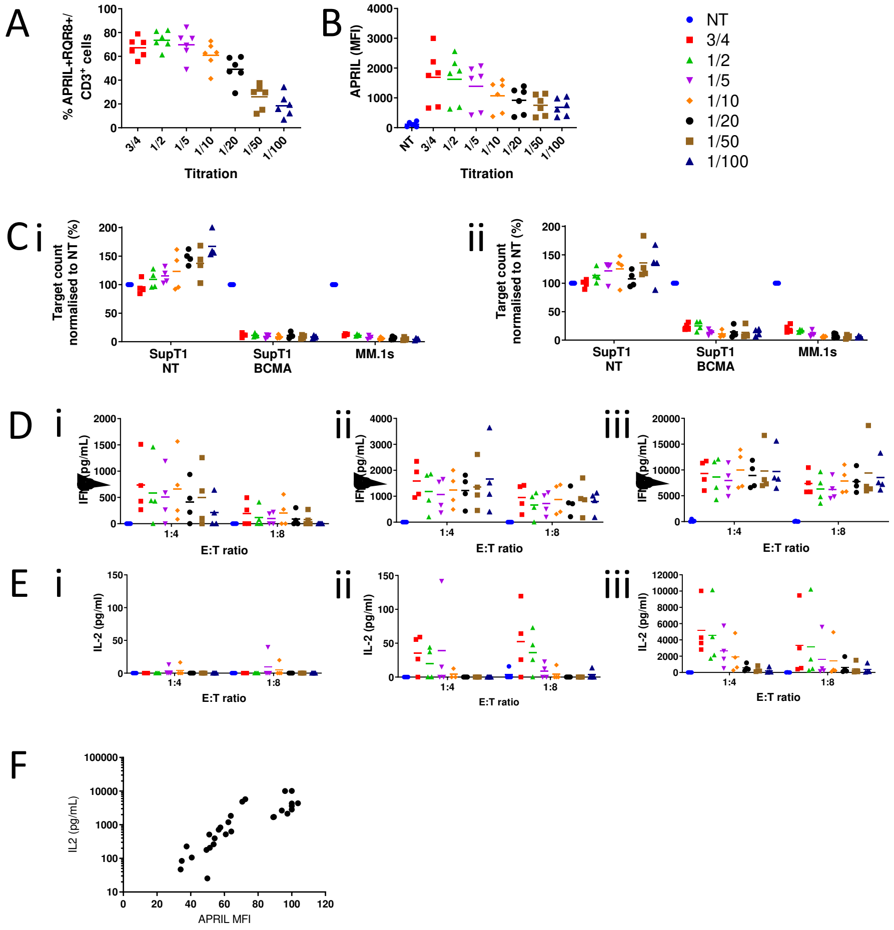

Supp Figure 6. Effect of transduction efficiency on APRIL CAR function. PBMCs from normal donors (n=4) were transduced with diluted viral supernatant which reduced transduction efficiency of transduced T cells (A) and reduced the expression of APRIL on the cell surface (B). Target kill at 24 hour by non transduced (NT) or APRIL CAR transduced T cells of unmanipulated SUPT1 cells or SUPT1 engineered to express low levels of BCMA (SUPT1 BCMA) or MM1s cells at 1:4 (Ci) or 1:8(Cii). IFNγ release at 24 hours cocultured at an E:T ratio of 1:4 with SUPT1NT(Di) SUPT1BCMA (Dii) or MM1s (Diii). IL2 secretion by T cells transduced with reducing titrations of viral supernatant on coculture (E:T ratio of 1:4) with SUPT1 NT (Ei), SUPT1 BCMA(Eii) and MM1s cells(Eiii). (F) APRIL MFI of APRIL CAR transduced PBMCs normalised to NT T cells, correlated to IL2 release at 1:4 coculture with MM1s cells at 24 hours (r=0.9055, $p<0.0001$ by Spearmans correlation).

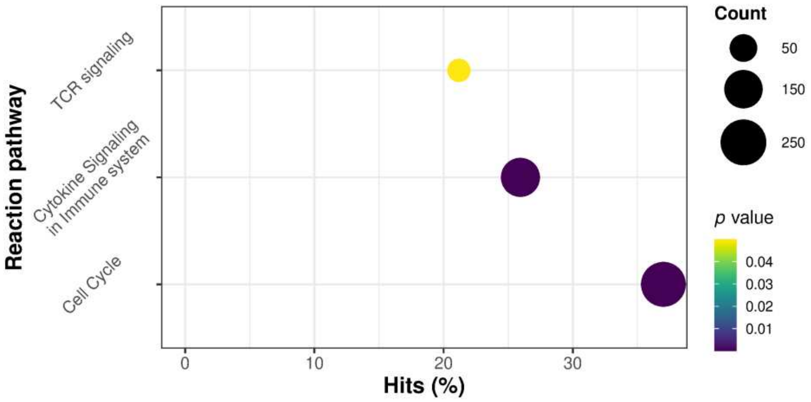

Supp Figure 7. GO Seq enrichment analysis of Reactome pathways using bulk RNA seq of non transduced PBMCS compared to those transduced with all CARs (APRIL CAR, bb2121 and LCAR-B38M) showed reduced expression of genes contained in TCR signalling (R-HSA-202424), cytokine signalling(R-HSA-1280215) and cell cycle(R-HSA-1640170). The dot plot shows the number (count) and proportion (hits) of differentially expressed genes in each pathway

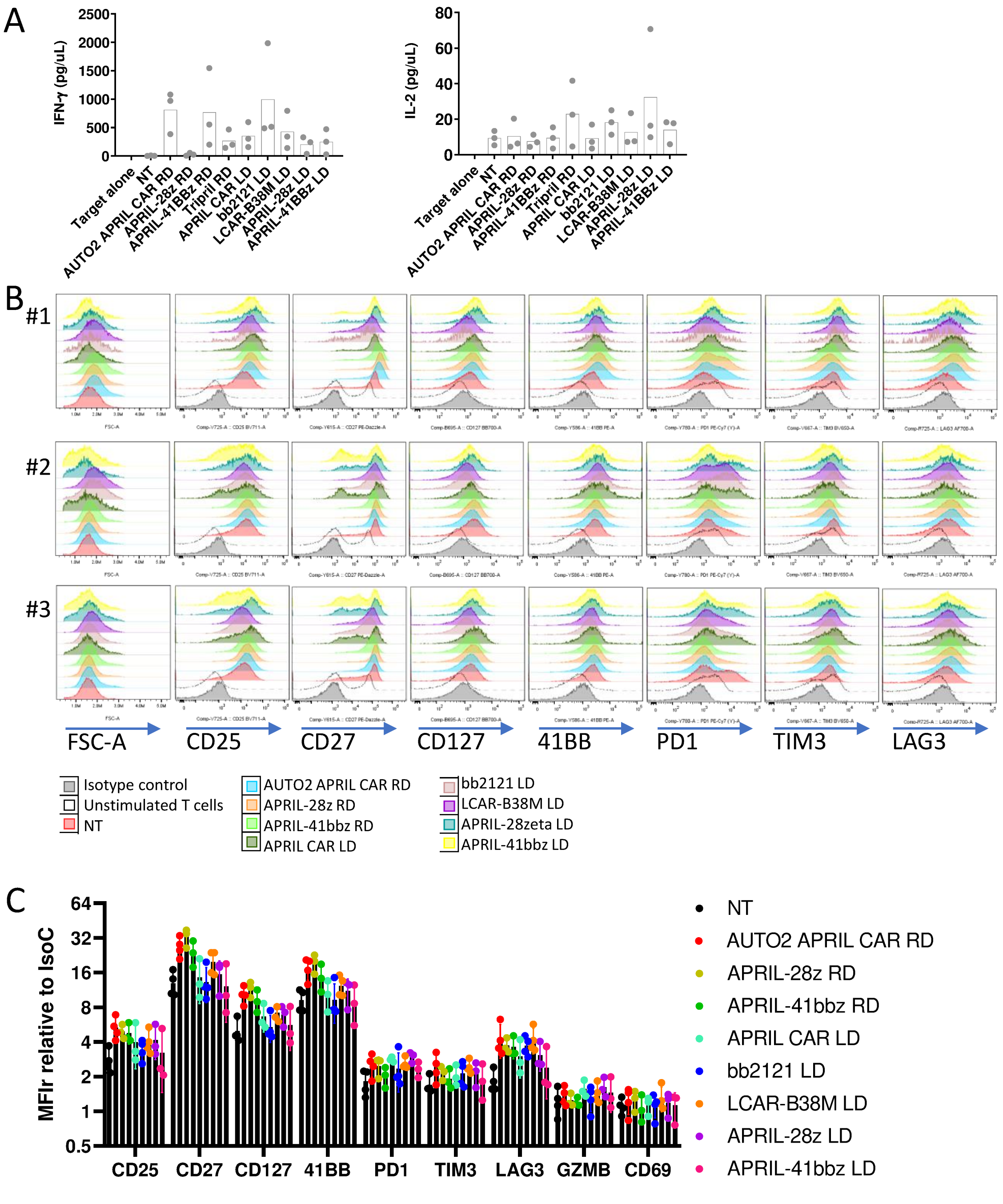

Supp Figure 8 Assessment of autoactivation of APRIL CAR variants, bb2121 and LCAR-B38M depicted in Figure 3a. (A)Cytokine release from PBMCs transduced with CAR constructs (retrovirus/RD transduced APRIL CAR variants or LD/lentivirus transduced bb2121, LCAR-B38M or APRIL variants) and then cocultured alone. IFNγ and IL2 from supernatant was quantified by ELISA. (B) Cells were then phenotyped by FACS at baseline (outlined in black) and after 7 days following initial activation and transduction with CAR constructs (C) Graph showing MFI of labelled proteins relative to isotype control. There was no significant difference in protein expression of the 3rd generation, RD transduced CAR used in the AUTO2 trial (labelled RD APRIL) and bb2121, LCAR-B38M or other variants of APRIL CAR construct (by multiple paired T tests and two stage step up method for multiple comparisons)

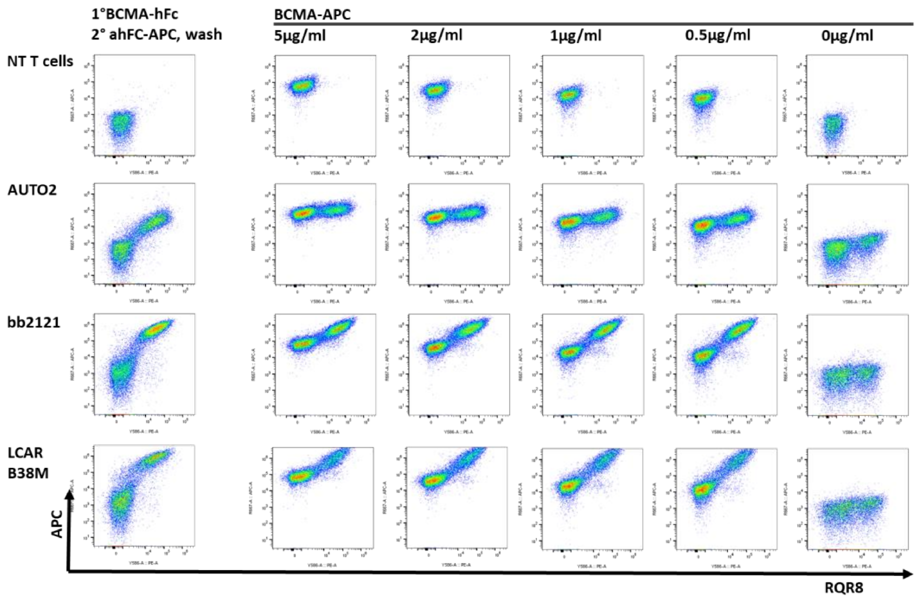

Supp Figure 9. Soluble BCMA binding by T cells expressing AUTO2, bb2121 and LCAR-B38M CARs was assessed by transducing PBMCs from three healthy donors with bicistronic constructs coexpressing RQR8 marker gene (in format RQR8_2A_CAR) before incubation with different concentration with APC conjugated BCMA. FACS plots shown of CAR transduced PBMCs stained with BCMA-Fc followed by secondary staining with anti hFc-APC and a wash step or APC conjugated BCMA (without wash step).

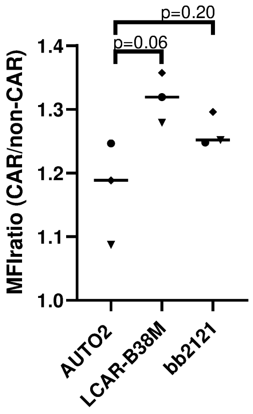

Supp Figure 10. Phosphoflow of activated CARs. T cells expressing AUTO2, bb2121 and LCAR-B38M CARs were incubated with H929 cells for 5 mins before fixation and FACs staining for phosphorylated ZAP70 (tyr319). Individual donors indicated by different symbols and MFI ratio of CARs vs non CAR T cells shown. Comparison of AUTO2 to other constructs made by paired T tests.
