# Supplementary material for: Limited efficacy of APRIL CAR in patients with multiple myeloma indicate challenges in the use of natural ligands for CAR T-cell therapy
Source: J Immunother Cancer. 2023 Jun 30;11(6):e006699. doi: 10.1136/jitc-2023-006699 (PMC10314698; doi:10.1136/jitc-2023-006699)
Supplement: Supplementary data [file jitc-2023-006699supp003.pdf]

Supplemental material BMJ Publishing Group Limited (BMJ) disclaims all liability and responsibility arising from any reliance placed on this supplemental material which has been supplied by the author(s) J Immunother Cancer

CLINICAL STUDY PROTOCOL

A SINGLE-ARM, OPEN-LABEL, MULTI-CENTRE, PHASE I/II STUDY EVALUATING THE SAFETY AND CLINICAL ACTIVITY OF AUTO2, A CAR T CELL TREATMENT TARGETING BCMA AND TACI, IN PATIENTS WITH RELAPSED OR REFRACTORY MULTIPLE MYELOMA

**Study Number:**	AUTO2-MM1
**Study Product:**	AUTO2 for IV infusion
**Development Phase:**	I/II
**Sponsor:**	Autolus Ltd<br>Forest House<br>58 Wood Lane<br>White City<br>London, W12 7RZ<br>United Kingdom
**Protocol Version:**	Version 5.0
**EudraCT Number:**	2016-003893-42
**Protocol Date:**	30 January 2018
**Compliance:**	This study will be conducted in accordance with standards of Good Clinical Practice (as defined by the International Conference on Harmonisation), ethical principles that have their origin in the Declaration of Helsinki and all applicable national and local regulations.

This protocol includes information and data that contain trade secrets and privileged or confidential information that is the property of the Sponsor (Autolus Ltd). This information must not be made public without written permission from Autolus Ltd. These restrictions on disclosure will apply equally to all future information supplied to you. This material may be disclosed to and used by your staff and associates as may be necessary to conduct the clinical study.

Lee L, *et al. J Immunother Cancer* 2023; 11:e006699. doi: 10.1136/jitc-2023-006699

ADMINISTRATIVE AND CONTACT INFORMATION

Sponsor: Autolus Ltd
Forest House
58 Wood Lane
White City
London
W12 7RZ
Tel: +44 (0)20 3829 6230

Contact List

The List of Service Providers for the study will be maintained separately by Autolus Ltd and kept in the Trial Master File/Sponsor Oversight File.

Primary Medical Monitor: Nushmia Khokhar, MD
Autolus Ltd
Telephone: +44 (0) 20 3829 6230
Mobile: +44 (0) 7736 895831
Email: n.khokhar@autolus.com

Secondary Medical Monitor: Vijay Peddareddigari, MD
Autolus Ltd
Telephone: +44 (0) 20 3829 6230
Mobile: +44 (0) 7736 895815
Email: v.reddy@autolus.com

Sponsor's Clinical Project Manager: Virginie Cerec, PhD
Autolus Ltd
Telephone: +44 (0) 20 3829 6230
Email: v.cerec@autolus.com

Notification of SAEs: SAE Fax number: +44 (0) 1252 842277

Email: Autolus@tmcpharma.com

24 Hour Safety Hotline: TMC Pharma
Address: Hartley, Witney, Hampshire, RG27 8AS, UK
Telephone: +44 (0)1252 842255

SPONSOR SIGNATURE PAGE

Study Title: A Single-Arm, Open-Label, Multi-Centre, Phase I/II Study Evaluating the Safety and Clinical Activity of AUTO2, a CAR T Cell Treatment Targeting BCMA and TACI, in Patients with Relapsed or Refractory Multiple Myeloma

Protocol Number: AUTO2-MM1

Version Number: Version 5.0

Version Date: 30 January 2018

I have read the protocol AUTO2-MM1 titled "A Single-Arm, Open-Label, Multi-Centre, Phase I/II Study Evaluating the Safety and Clinical Activity of AUTO2, a CAR T Cell Treatment Targeting BCMA and TACI, in Patients with Relapsed or Refractory Multiple Myeloma" and confirm that, to the best of my knowledge, the protocol accurately describes the design and conduct of the study.

Vijay Peddareddigari

Signature

Vijay Peddareddigari, MD
Chief Medical Officer

06 FEB 2018

Date
(DD MMM YYYY)

INVESTIGATOR SIGNATURE PAGE

Study Title: A Single-Arm, Open-Label, Multi-Centre, Phase I/II Study Evaluating the Safety and Clinical Activity of AUTO2, a CAR T Cell Treatment Targeting BCMA and TACI, in Patients with Relapsed or Refractory Multiple Myeloma

Protocol Number: AUTO2-MM1

Version Number: Version 5.0

Version Date: 30 January 2018

I have read the protocol AUTO2-MM1 titled “A Single-Arm, Open-Label, Multi-Centre, Phase I/II Study Evaluating the Safety and Clinical Activity of AUTO2, a CAR T Cell Treatment Targeting BCMA and TACI, in Patients with Relapsed or Refractory Multiple Myeloma”. By signing this protocol, I agree to conduct the clinical study, after approval by an Institutional Review Board or Independent Ethics Committee (as appropriate), in accordance with the protocol, the principles of the Declaration of Helsinki (2008), the standards of Good Clinical Practice (as defined by the International Conference on Harmonisation) and applicable regulatory requirements.

Changes to the protocol will only be implemented after written approval is received from Autolus Ltd and the Institutional Review Board or Independent Ethics Committee (as appropriate), with the exception of medical emergencies. I will ensure that study staff fully understand and follow the protocol.

Signature<br><br><Name of Investigator><br><Affiliation of Investigator>	Date<br>(DD MMM YYYY)

PROTOCOL SYNOPSIS

**Title**	A Single-Arm, Open-Label, Multi-Centre, Phase I/II Study Evaluating the Safety and Clinical Activity of AUTO2, a CAR T Cell Treatment Targeting BCMA and TACI, in Patients with Relapsed or Refractory Multiple Myeloma.
**Study Number**	AUTO2-MM1
**Sponsor**	Autolus Ltd
**Development Phase**	I/II
**Study Product**	AUTO2, an advanced therapy investigational medicinal product, comprising of autologous enriched T Cells retrovirally transduced to express a proliferation inducing ligand (APRIL) Chimeric Antigen Receptor (CAR) and the RQR8 safety switch.
**Study Population**	Patients with confirmed diagnosis of multiple myeloma (MM) as per the International Myeloma Working Group (IMWG) who have relapsed or become refractory after exposure to alkylator therapy or monoclonal antibody, immunomodulatory drug and proteasome inhibitor.
**Study Duration**	The study will take approximately 4.5 years from recruitment to the last patient's last 24-month follow-up visit. The end of the trial will be 24 months after the last patient has received an AUTO2 infusion or the last patient last visit if this occurs earlier due to patient death or withdrawal.
**Overview**	A single-arm, open-label, multi-centre, Phase I/II dose-escalation and expansion study to determine the safety and clinical activity of APRIL CAR T cells administered intravenously (IV) in adults with MM. A proliferation inducing ligand is a ligand for B cell maturation antigen (BCMA), and for the transmembrane activator and calcium modulator and cyclophilin ligand interactor (TACI), present on plasmablasts and plasma cells. A proliferation inducing ligand CAR is a third generation CAR construct with endodomains of CD28, OX40 and CD3 zeta. The receptor-binding domain of APRIL targets both BCMA and TACI. AUTO2 is autologous enriched T cell product retrovirally transduced to express a proliferation inducing ligand (APRIL) CAR and the RQR8 safety switch. This first-in-human study will assess the safety of AUTO2, determine the appropriate dose for Phase II and evaluate the preliminary efficacy of the AUTO2 in relapsed or refractory MM patients.

<table>
<tr><td>Primary Objective(s) and Endpoints</td><td>The primary objectives of the study are as follows:
<table>
<tr><th>Objectives</th><th>Endpoints</th></tr>
<tr><td colspan="2">Phase I</td></tr>
<tr><td>To assess the safety and tolerability of AUTO2 administration.</td><td>Incidence of Grade 3 to 5 toxicity occurring within the dose-limiting toxicity (DLT) period (28 days post AUTO2 infusion).</td></tr>
<tr><td>To identify the recommended Phase II dose and maximum tolerated dose (MTD), if an MTD exists, of AUTO2.</td><td>Frequency of DLT and the persistence of AUTO2.</td></tr>
<tr><td colspan="2">Phase II</td></tr>
<tr><td>To evaluate the anti-tumour effect of AUTO2.</td><td>Best Overall Response post-AUTO2 infusion.</td></tr>
</table></td></tr>
<tr><td>Study Design</td><td>This is a Phase I/II, open-label, multi-centre study to characterise the safety and clinical activity of APRIL CAR T cells when administered to patients with relapsed or refractory MM. The study will consist of 2 parts, a Phase I/dose escalation followed by a Phase II/expansion. Both parts of the study will involve patients going through the following 5 sequential stages: screening, leukapheresis, pre-conditioning, treatment and follow-up.

Phase I (Dose Escalation): To identify the optimal dose (based on safety, tolerability and antitumour activity) of AUTO2 using an accelerated titration design (Simon et al. 1997). Up to 4 cohorts and a maximum of 24 patients with MM will be enrolled. Doses from 15×10^6 to 450×10^6 RQR8/APRIL CAR positive T cells will be evaluated.

Phase II (Dose Expansion): To further characterise the safety and assess the efficacy of AUTO2 at the recommended dose identified in Phase I, 30 patients will be treated in the dose expansion phase.

Biomarkers relating to the CAR T cells and tumours will be evaluated in all patients. All patients enrolled in Phase I and II will attend clinic visits for up to 24 months (or less in the event of early discontinuation) post AUTO2 infusion for study-specific assessments including adverse event (AE) assessments, physical examination, and laboratory and immunology tests.

After completion of the 24-month follow-up period or following AUTO2 treatment and early withdrawal from this study, all patients will be followed until death or for up to 15 years from treatment administration under a separate long-term follow-up study protocol.</td></tr>
</table>

Lee L, et al. J Immunother Cancer 2023; 11:e006699. doi: 10.1136/jitc-2023-006699

**Number of Patients**	Up to 80 patients in total are expected to be enrolled into both the escalation and expansion phases of the study, and up to 54 patients in total are anticipated to receive the AUTO2 therapy.<br>• **Phase I (Escalation):** An accelerated titration design (in which a single patient will be dosed at the lowest dose level, before reverting to a standard 3+3 dose escalation design upon observation of Grade 2 or higher toxicity that is probably or definitely related to AUTO 2 during the DLT period or at dose level 2 whichever is earlier); up to 24 patients in total (3 to 6 per dose cohort).<br>• **Phase II (Expansion):** Dose expansion: 30 patients in total.<br>Phase II of the study will follow Simon's 2-stage optimal design.
**Key Criteria for Eligibility**	Only patients whose leukapheresis sample has been successful in generating AUTO2 in adequate quantity will be treated. Some key criteria for patient eligibility are summarised as follows:<br>• Confirmed diagnosis of MM as per IMWG.<br>• Male or female patients aged 18 or over and willing and able to give written informed consent to the current study.<br>• Relapse or refractory disease and have had at least 3 different prior lines of therapy including proteasome inhibitor (e.g. bortezomib or carfilzomib) and immunomodulatory therapy (IMiD; e.g. thalidomide, lenalidomide or pomalidomide) and alkylator or monoclonal antibody, or have "double refractory" disease to a proteasome inhibitor and IMiD, defined as progression on or within 60 days of receiving these agents.<br>• Eastern Cooperative Oncology Group (ECOG) Performance Status 0 to 1.<br>• Patients should have adequate bone marrow function without the requirement for ongoing blood product or granulocyte-colony stimulating factor support.<br>• Women who are pregnant or lactating will be excluded.<br>• Prior treatment with investigational or approved gene therapy or cell therapy products or allogenic stem cell transplant will be excluded.<br>• Patients with clinically significant, uncontrolled heart disease, history or evidence of deep vein thrombosis or pulmonary embolism requiring ongoing therapeutic anticoagulation at the time of pre-conditioning and chronic renal impairment requiring dialysis, or creatinine clearance <30 mL/min will be excluded.<br>• Patients with significant liver disease: alanine aminotransferase or aspartate aminotransferase ≥3 × upper limit of normal (ULN), or total bilirubin ≥25 µmol/L (1.5 mg/dL), except in patients with Gilbert's syndrome, or evidence of end-stage liver disease (e.g. ascites, hepatic encephalopathy) will be excluded.<br>• Patients with active infectious bacterial or viral disease (hepatitis B virus, hepatitis C virus, human immunodeficiency virus, human T-lymphotropic virus or syphilis) requiring treatment will be excluded.<br>• Patients that received any anti-myeloma therapy within the last

	7 days prior to pre-conditioning or leukapheresis will be excluded.
**Study Product Dose, Dosing Regimen and Administration**	Eligible patients will receive AUTO2 IV following pre-conditioning treatment. The AUTO2 product contains both transduced (RQR8/APRIL CAR positive) and non-transduced cells. The dose is expressed as the number of RQR8/APRIL CAR positive T cells. Four dose cohorts are planned in Phase I:<br>• Cohort 1, Dose Level 1: 15 × 10^6 RQR8/APRIL CAR positive T cells<br>• Cohort 2, Dose Level 2: 75 × 10^6 RQR8/APRIL CAR positive T cells<br>• Cohort 3, Dose Level 3: 225 × 10^6 RQR8/APRIL CAR positive T cells<br>• Cohort 4, Dose Level 4: 450 × 10^6 RQR8/APRIL CAR positive T cells<br>The Safety Evaluation Committee will convene after the first and third patient as well as at the end of each cohort to decide on the next dose level, after the last cohort in the escalation phase to decide on the dose level for Phase II, and at the interim analysis stage. When a recommended Phase II dose decision is made by the Safety Evaluation Committee, the decision will need to be reviewed and endorsed by the Independent Data Monitoring Committee prior to opening Phase II. On occasion, a patient may receive re-treatment upon satisfying re-treatment criteria.
**Pre-conditioning Treatment**	All patients will receive a pre-conditioning regimen using fludarabine 30 mg/m^2 IV over 30 minutes immediately followed by cyclophosphamide 300 mg/m^2 IV over 30 minutes. Both these drugs will be given on Days -6, -5, and -4 before AUTO2 infusion.
**Safety Evaluation**	Safety will be assessed by physical examinations, ECOG status, laboratory tests, vital signs, electrocardiograms, AE and SAE monitoring, and concomitant medication usage. The severity of AEs will be assessed using the National Cancer Institute Common Terminology Criteria for Adverse Events (Version 4.03), with the exception of cytokine release syndrome (CRS), which will be graded according to the CRS grading.
**Efficacy Evaluation**	Efficacy will be evaluated as per IMWG criteria.
**Biomarker Evaluation**	Biomarker evaluation will include the following:<br>• Expansion and persistence of RQR8/APRIL CAR positive T cells as determined by quantitative polymerase chain reaction and/or flow cytometry.<br>• Depletion of BCMA+/TACI+ cell compartment as determined by flow cytometry and/or immunohistochemistry on bone marrow.
**Special Study Procedures**	All patients will undergo leukapheresis for AUTO2 generation.

**Statistical Analysis**	The data will be summarised using descriptive statistics. Continuous variables will be summarised using the number of observations, mean, standard deviation, median, and range as appropriate. Categorical values will be summarised using the number of observations and percentages as appropriate. Time-to-event endpoints will be estimated using Kaplan-Meier methodology.<br>The Phase II part of the study will enrol up to 30 evaluable patients. With this number of patients, assuming an overall response rate of 30%, the lower bound of the 2-sided 95% confidence interval for overall response rate will exclude 14%.<br>Data from all treated patients (safety analysis set) will be used for safety analyses and will be summarised by dose level. All treated patients (efficacy analysis set) and other subsets will be defined for the efficacy analyses.
**Interim Analyses**	In the Phase II part of the study, an interim analysis will be performed after 10 patients are enrolled and evaluable. The study will be stopped at this first stage if no more than 1 response (as per the IMWG) has been observed.

SCHEDULE OF ASSESSMENTS

Visits / Assessments	Screen 1	Leuka pheresis	Screen 2	Pre-conditioning	Treatment Phase				Follow-up Phase	End of Study***
	**Day -84 to -35**	**Day -84 to -35**	**Day -14 to -7**	**Day -6, -5, -4 (±1d)**	**Day 0**	**Day 1 then q.a.d. in hospital* (±1d)**	**Weekly after discharge (±2d)**	**End of DLT period** (+3d)**	**Month 2, 3, 4, 5, 6, 8, 10, 12, 15, 18 & 24 (±7d)**	**Year 2 (±14d)**
Informed consent	X									
Demographic data [1]	X									
Eligibility criteria [2]	X		X	X [D-6]						
Medical/Multiple Myeloma history [3]	X									
**EXAMINATIONS/INVESTIGATIONS**										
ECOG status	X			X [D-6]				X	X	X
Physical examination [4]	X			X [D-6]	X	X [4]				
Weight	X	X		X			X	X	X	X
Vital signs [5]	X			X [D-6]	X	X	X	X	X	X
12-lead ECG [6]	X				X [6]			X		
ECHO or MUGA [7]	X									
**DISEASE EVALUATION**										
Beta 2 microglobulin	X									
Serum quantitative immunoglobulins [8]	X									
Serum free light chain [9]	X		X [9]		X [9]			X [9]	X [9]	
Serum immunofixation [10]	X									
Paraprotein in Serum (SPEP)	X		X		X			X	X	
Paraprotein in Urine (UPEP) [11]	X		X		X			X	X	
Urine immunofixation [10]	X									

Visits / Assessments	Screen 1	Leuka pheresis	Screen 2	Pre-conditioning	Treatment Phase				Follow-up Phase	End of Study***
	Day -84 to -35	Day -84 to -35	Day -14 to -7	Day -6, -5, -4 (±1d)	Day 0	Day 1 then q.a.d. in hospital* (±1d)	Weekly after discharge (±2d)	End of DLT period** (+3d)	Month 2, 3, 4, 5, 6, 8, 10, 12, 15, 18 & 24 (±7d)	Year 2 (±14d)
Bone marrow aspirate and biopsy [12]	X [12]		X					X	X M3, M6, M12, M18, M24	
Imaging [13]			X							
**SAFETY AND EFFICACY EVALUATIONS**										
Haematology [14]	X	X	X	X D-6	X	X	X	X	X M2, M3, M6	
Biochemistry [15]	X	X	X	X D-6	X	X D7	X	X	X M2	
CRP and ferritin [16]				X D-6	X	X	X	X		
Coagulation [17]	X				X	X [16]				
Infectious disease screen [18]	X	X								
Pregnancy test [19]	X		X	X D-6	X			X	X M3, M6, M12	
**PHARMACODYNAMICS AND BIOMARKER ASSAYS**										
Serum for cytokines [20]	X		X	X D-6, D-4 [20]	X	X	X	X	X M3	
Blood for RQR8/APRIL CAR T cells [21]			X		X [21]	X	X	X	X M2, M3, M6, M12, M15, M18, M24	
Blood for phenotyping and genotyping [21]			X			X D7	X W1	X	X M3, M6, M12	
Blood for RCR and insertional mutagenesis			X					X	X M3, M12, M24	
**TREATMENTS**										
Cyclophosphamide/Fludarabine				X						
AUTO2 infusion					X					
Adverse event [22]	X	X	X	X	X	X	X	X	X	X
Concomitant medication [22]			X	X	X	X	X	X		

APRIL=a proliferation inducing ligand; CAR=chimeric antigen receptor; CPK=creatine phosphokinase; CRP=C-reactive protein; DLT=dose limiting toxicity; ECG=electrocardiogram; ECHO=echocardiogram; ECOG=Eastern Cooperative Oncology Group; MUGA=multiple gated acquisition; q.a.d=every other day of a treatment phase (e.g. Day 3, Day 5, Day 7 and Day 9 during hospital stay or outpatient visits); RCR=replication competent retrovirus; SPEP=serum protein electrophoresis; UPEP=urine protein electrophoresis.

*: In the event of prolonged hospitalisation beyond the 10 recommended days, assessments will be collected every other day after AUTO2 dose, as per schedule of assessment.

**: End of DLT period set as follow: 28 days after AUTO2 dose.

***: The End of Study visit is to be performed upon completion of all study visits or in case of early withdrawal.

Patients who have received AUTO2 and have discontinued or completed the study may continue to be monitored for AUTO2 treatment-related serious adverse events and adverse events until they enrol onto the long-term follow-up study (AUTO-LT1) and the End of Study visit will be delayed. Patient eligible for re-treatment will undergo assessments from Screen 2 onwards unless repeat leukapheresis is required in which case assessments will be performed from leukapheresis visit onwards, as per schedule of assessments.

X ^{D7}: Test to be performed on a particular day of the schedule rather than systematically at every visit. Please refer to the number to determine the day of assessment.

X ^{W1}: Tests to be performed only at Week 1 after discharge. Please refer to the number to determine the day of assessment.

X ^{M3}: Tests to be performed only at the Month 3 visit. Please refer to the number to determine the day of assessment.

1.	Demographic data:	Race/ethnicity, height, age and gender.
2.	Eligibility criteria:	To be re-assessed prior to pre-conditioning during screen visit 2.
3.	Medical/Multiple Myeloma history:	To include clinically significant diseases, surgeries, cancer history (including prior cancer therapies and procedures) and prior medications (e.g. prescription drugs, over-the-counter drugs, herbal/homeopathic remedies, nutritional supplements). Record disease status at last assessment prior to screening. Obtain histological confirmation of disease diagnosis (pathology report) and if available, record cytogenetic biomarkers.
4.	Physical examination:	Complete physical examination to be performed at screening then focused examination as appropriate at following visits if indicated. From Day 0 onwards, only record physical examination if clinically significant and report as an AE as appropriate.
5.	Vital signs:	Temperature, systolic and diastolic blood pressure, pulse rate and respiratory rate will be recorded while the patient is in a seated position. On Day 0 record vital signs immediately prior to AUTO2 infusion and every 15 min (± 5 min) for the 1st hour and every 30 minutes (± 10 min) for the next 3 hours post AUTO2 infusion, and thereafter monitored as per hospital policy but no less than 3 times a day. Record weight as per schedule of assessment above. Body Surface Area to be recorded only on Day -6.
6.	12-lead ECG:	On Day 0, recording to be performed prior to AUTO2 infusion.
7.	ECHO or MUGA:	To be performed at screening and to be repeated if clinically indicated. Same method should be used throughout the study.
8.	Serum quantitative immunoglobulins (Ig):	IgG, IgA, IgM. To be repeated as clinically indicated.
9.	Serum free light chain:	Serum free light chain assessments should be performed and repeated at the indicated time points in the schedule of assessments for patients who are serum free light chain positive i.e. light chain positive multiple myeloma or to confirm complete response (CR) in all patients and when disease progression is suspected.
10.	Immunofixation:	Serum and urine immunofixation to be repeated at time of suspected CR or suspected disease progression.
11.	Paraprotein in urine:	24h urine collection assessments should be performed at the indicated time points in the schedule of assessments and when CR or disease progression is suspected.
12.	Bone marrow aspirate and biopsy:	Percentage of plasma cells in the bone marrow will be assessed by morphology as per local institution standards. Minimal residual disease assessment will be performed on bone marrow aspirate at a central laboratory. Other biomarkers will be measured on bone marrow aspirate and biopsy at a central laboratory. All assessments to be performed at the indicated time points in the schedule of assessments. An additional bone marrow aspirate and biopsy is also required to confirm CR. Optional sample at Screen 1 and at time of disease progression. Refer to laboratory manual for collection instructions.
13.	Imaging:	Assessment of plasmacytomas and bone lesions at baseline using computerised tomography or positron emission tomography-computerised tomography or magnetic resonance imaging scan as per standard of care. To be repeated as clinically indicated or in case of suspected CR or disease progression. Baseline assessment does not need to be repeated if imaging is within 4 weeks prior to pre-conditioning.

14.	Haematology:	Haemoglobin, red blood cell count, platelet count, white blood cell count with differential (neutrophils, eosinophils, lymphocytes, monocytes, and basophils). Test to be performed prior to chemotherapy on pre-conditioning days and prior to AUTO2 infusion on Day 0.
15.	Biochemistry:	Sodium, phosphate, potassium, alanine aminotransferase, aspartate aminotransferase, creatinine, creatine phosphokinase (CPK), lactate dehydrogenase, total bilirubin, calcium, total protein, albumin. All tests must be performed prior to AUTO2 infusion on Day 0. Glomerular filtration rate and creatinine clearance should be calculated at screening as per institutional preferred method.
16.	C-reactive protein and ferritin:	To be measured up to end of DLT period only.
17.	Coagulation:	Prothrombin time, international normalised ratio, activated prothrombin time, fibrinogen after baseline assessment may be repeated if patient experiences severe cytokine release syndrome.
18.	Infectious disease screen:	Human immunodeficiency virus, hepatitis B, hepatitis C, human T-lymphotropic virus-1, human T-lymphotropic virus-2 and syphilis and any other infectious agents required as per national and/or institutional guidelines. First test to be performed max 30 days before leukapheresis with results available prior to leukapheresis and second test to be performed on the day of leukapheresis or within 7 days after leukapheresis.
19.	Pregnancy test:	Serum (β-human chorionic gonadotropin) or urine pregnancy testing for women of childbearing potential.
20.	Serum for cytokines and biomarkers:	Serum cytokine sample to be taken prior to pre-conditioning on Day -6 and post pre-conditioning on Day -4. To be collected daily during cytokine release syndrome. During hospital stay, sample collection to be performed every other day (±1 day). Refer to laboratory manual for collection instructions. Additional optional samples maybe collected between Day -4 (±1 day) and Day 0 if patient is hospitalised or in ambulatory care. Based on emerging data, additional optional blood samples may be collected to assess the drug levels of fludarabine / cyclophosphamide prior to pre-conditioning on Day-6 and approximately 1 hour or later post pre-conditioning on Day -6 and -4.
21.	Blood for RQR8/APRIL CAR T cells:	Sample to be taken prior to AUTO2 infusion on Day 0. During hospital stay, sample collection to be performed every other day (±1 day). An optional sample will be collected at time of disease progression. Refer to laboratory manual for collection instructions.
22.	Adverse events and con med:	Collected on an ongoing basis throughout the study. Please refer to Sections 6 and 10.3.1 for specifics about the reporting period.

Note: The total estimated volume of blood collected for safety, biomarkers and immunological assessments (with the exception of the leukapheresis procedure) across the entire study will not normally exceed 665 mL. The maximum volume of blood collected on any 1 day will unlikely exceed 50 mL. No more than 300 mL of blood will be collected in any 28-day period.

TABLE OF CONTENTS

PROTOCOL SYNOPSIS 5
SCHEDULE OF ASSESSMENTS 10
TABLE OF CONTENTS 14
LIST OF TABLES 19
LIST OF FIGURES 19
LIST OF ABBREVIATIONS 20
1 INTRODUCTION 22
1.1 MULTIPLE MYELOMA 22
1.2 B CELL MATURATION ANTIGEN AND TRANSMAMBRANE ACTIVATOR AND CALCIUM-MODULATOR AND CYCLOPHILIN LIGAND INTERACTOR BIOLOGY AND EXPRESSION IN MULTIPLE MYELOMA 23
1.3 CLINICAL EXPERIENCE WITH CAR T THERAPIES IN MULTIPLE MYELOMA 23
1.4 DESCRIPTION OF RQR8/APRIL CAR T CELLS: AUTO2 25
1.5 SUMMARY OF NONCLINICAL STUDIES 25
1.5.1 *In Vitro* and *In Vivo* Pharmacology 26
1.5.2 Toxicology 27
1.6 SUMMARY OF CLINICAL STUDIES 28
1.7 OVERALL RATIONALE FOR THE STUDY 29
2 STUDY OBJECTIVES AND ENDPOINTS 30
2.1 PRIMARY OBJECTIVES AND ENDPOINTS 30
2.2 SECONDARY OBJECTIVES AND ENDPOINTS 30
2.3 EXPLORATORY OBJECTIVES 31
3 STUDY OVERVIEW 32
3.1 STUDY DESIGN AND METHODOLOGY 32
3.1.1 Study Overview 32
3.1.2 Study Duration 33
3.1.3 Phase I Dose Escalation 33
3.1.4 Dose Limiting Toxicity 35
3.1.5 Planned Dose Levels 37
3.1.6 Phase II Dose Expansion 37
3.2 RATIONALES FOR STUDY DESIGN, DOSE AND POPULATION 37
3.2.1 Study Design Rationale 37
3.2.2 Study Population Rationale 38
3.2.3 Cyclophosphamide and Fludarabine Pre-conditioning Rationale 38
3.2.4 Starting Dose Rationale 41
3.2.5 Dosing and Assessment Rationale 42
3.2.6 Biomarker Collection and Assessment Rationale 42
3.3 RISKS AND MITIGATION STRATEGY 42

4 PATIENT POPULATION....46
4.1 NUMBER OF PATIENTS....46
4.2 INCLUSION CRITERIA....46
4.3 EXCLUSION CRITERIA....47
4.4 EARLY DISCONTINUATION....49
4.4.1 Discontinuation of Study....49
4.4.2 Patient Withdrawal from the Study....50
4.4.3 Withdrawal from the use of samples in future research....50
4.4.4 Procedures for Handling Withdrawals....50
4.4.5 Replacement Policy....51
5 STUDY DRUG AUTO2....52
5.1 DRUG DESCRIPTION....52
5.2 AUTO2 MANUFACTURING....52
5.3 PACKAGING AND LABELLING....53
5.4 SUPPLY AND STORAGE....53
5.5 ACCOUNTABILITY AND DESTRUCTION....53
6 CONTRAINDICATIONS, CONCOMITANT MEDICATIONS AND PROHIBITED THERAPIES....55
6.1 ALLOWED CONCOMITANT MEDICATIONS/THERAPIES....55
6.2 PROHIBITED AND CAUTIONARY THERAPIES....56
6.3 OVER-DOSAGE....56
6.4 DIETARY AND LIFESTYLE RESTRICTIONS....56
7 STUDY PROCEDURES....58
7.1 LEUKAPHERESIS....58
7.2 PRE-CONDITIONING CHEMOTHERAPY FOR AUTO2 THERAPY....58
7.2.1 Supply of Cyclophosphamide and Fludarabine....59
7.2.2 Accountability of Cyclophosphamide and Fludarabine....59
7.3 AUTO2 TREATMENT AND PATIENT MONITORING....59
7.3.1 Treatment Allocation and Blinding....59
7.3.2 AUTO2 Administration....59
7.3.3 Monitoring During and After Drug Administration....60
7.3.4 Dose Delay....60
7.3.5 Interruption of AUTO2 Infusion....60
7.3.6 Re-treatment of Patients....61
8 GUIDELINES FOR PREVENTION, MONITORING AND MANAGEMENT OF ADVERSE EVENTS....62
8.1 GENERAL SUPPORTIVE CARE GUIDELINES FOR PATIENTS RECEIVING CAR T CELL THERAPY....62
8.2 PRE- AND POST-INFUSION SUPPORTIVE THERAPY....63
8.3 TREATMENT OF INFUSION RELATED REACTIONS....64
8.4 GRADING AND MANAGEMENT OF CYTOKINE RELEASE SYNDROME....66

8.5 MANAGEMENT OF TUMOUR LYSIS SYNDROME 68
8.6 GRADING AND MANAGEMENT OF NEUROTOXICITY 69
8.7 MANAGEMENT OF THROMBOCYTOPENIA 73
8.8 MANAGEMENT OF IMMUNE RELATED ADVERSE EVENTS DUE TO ON-TARGET BUT OFF-TUMOUR TOXICITY 73
8.9 RITUXIMAB RESCUE THERAPY 73

9 STUDY VISITS AND ASSESSMENTS 75

9.1 STUDY OVERVIEW 75
9.1.1 Screening Phase 75
9.1.2 Leukapheresis Phase 75
9.1.3 Pre-conditioning Phase 75
9.1.4 Treatment Phase 76
9.1.5 Follow-up Phase 76
9.1.6 Long-term Follow-up 76
9.2 DETAILS OF STUDY ASSESSMENTS 76
9.2.1 Demographic and Baseline Assessments 76
9.2.2 Safety Evaluations 77
9.2.3 Pharmacodynamics and Biomarker Evaluation 79
9.2.4 Efficacy Evaluation 80
9.2.5 Blood Volume Collections 83

10 SAFETY MANAGEMENT 84

10.1 ADVERSE EVENTS DEFINITIONS 84
10.1.1 Adverse Event 84
10.1.2 Adverse Reactions 84
10.1.3 Serious Adverse Events 85
10.1.4 Suspected Unexpected Serious Adverse Reactions 86
10.1.5 Adverse Events of Special Interest 86
10.2 ADVERSE EVENTS ASSESSMENT 86
10.2.1 Severity of Adverse Events 87
10.2.2 Assessment of Relationship 87
10.3 ADVERSE EVENTS REPORTING PROCEDURES 88
10.3.1 All Adverse Events 88
10.3.2 Serious Adverse Events 89
10.3.3 Adverse Events of Special Interest 91
10.3.4 Management of Pregnancy 91
10.3.5 Other Significant Events Reporting 92
10.3.6 Reporting Exemptions 92
10.4 SAFETY STOPPING CRITERIA FOR THE CLINICAL TRIAL 93
10.5 SAFETY EVALUATION COMMITTEE 93
10.6 INDEPENDENT DATA MONITORING COMMITTEE 94

11 STATISTICS 95
11.1 SAMPLE SIZE ESTIMATION 95
11.2 DESCRIPTION OF ANALYSIS DATASETS 95
11.2.1 Full Analysis Set 95
11.2.2 Safety Analysis Set for the Study and for Study Treatment 95
11.2.3 Efficacy Analysis Set 95
11.3 STATISTICAL ANALYSES AND METHODS 95
11.3.1 Primary Endpoints 96
11.3.2 Secondary Endpoints 97
11.3.3 Exploratory 98
11.3.4 Immunogenicity Data 98
11.3.5 Pharmacokinetics 98
11.3.6 Other Analyses 98
11.3.7 Interim Analysis 98
12 DATA QUALITY CONTROL AND ASSURANCE 99
12.1 COMPLIANCE WITH THE PROTOCOL AND PROTOCOL REVISIONS 99
12.2 PROTOCOL VIOLATIONS AND DEVIATIONS 99
12.3 MONITORING 99
12.4 AUDITS AND SITE INSPECTIONS 100
13 DATA HANDLING AND RECORD KEEPING 101
13.1 DATA MANAGEMENT 101
13.2 STUDY DOCUMENTATION AND RETENTION OF RECORDS 101
14 CLINICAL TRIAL AGREEMENT, FINANCE AND INSURANCE 102
15 ETHICAL AND REGULATORY CONSIDERATIONS 103
15.1 ETHICS COMMITTEE REVIEW AND APRROVAL 103
15.2 REGULATORY AUTHORITY REVIEW AND APPROVAL 103
15.3 INVESTIGATOR RESPONSIBILITIES 103
15.3.1 Overall Responsibilities 103
15.3.2 Site Review 103
15.3.3 Informed Consent 103
15.3.4 Delegation of Investigator Duties 104
15.3.5 Communication with Independent Ethics Committee (IEC)/Institutional Review Board (IRB) 104
15.3.6 Confidentiality of Trial Documents and Patient Records 105
15.4 LONG-TERM RETENTION OF SAMPLES FOR ADDITIONAL FUTURE RESEARCH 105
16 STUDY COMPLETION 106
17 PUBLICATION OF DATA AND PROTECTION OF TRADE SECRETS 107
18 REFERENCES 108
19 APPENDICES 115

LIST OF TABLES

Table 1: Primary Objectives and Endpoints for Phase I ... 30
Table 2: Primary Objectives and Endpoints for Phase II ... 30
Table 3: Secondary Objectives and Endpoints ... 30
Table 4: 3+3 Dose Escalation Decision Rules ... 35
Table 5: Treatment Cohorts ... 37
Table 6: Leukapheresis – Risks and Mitigation Strategy ... 43
Table 7: Pre-conditioning Chemotherapy – Risks and Mitigation Strategy ... 43
Table 8: AUTO2 Infusion – Risks and Mitigation Strategy ... 44
Table 9: General Supportive Care Guidelines for Patients Receiving CAR T Cell Therapy ... 62
Table 10: Pre- and Post-infusion Medications ... 64
Table 11: Guidelines for the Management of Infusion-related Reactions ... 65
Table 12: Severity Grading and Management of CRS ... 66
Table 13: Definitions and Doses of Vasopressors ... 67
Table 14: Pharmacologic Management of CRS ... 68
Table 15: Grading of Neurological AEs and CAR-T-Cell-Related Encephalopathy Syndrome (CRES) ... 70
Table 15A: Management and Follow-up of Neurological AEs ... 70
Table 15B: Recommendations for the Management of Status Epilepticus after CAR T Cell Therapy ... 72
Table 15C: Recommendation for Management of Raised Intracranial Pressure (ICP) after CAR T Cell Therapy ... 72
Table 16: Clinical Laboratory Tests ... 78
Table 17: Severity Grading of AEs Not Listed on the NCI CTCAE Grading System ... 87
Table 18: Reporting Period for All AEs ... 88

LIST OF FIGURES

Figure 1: Structure of the Constructs Expressed on AUTO2 ... 25
Figure 2: Efficacy of RQR8-APRIL CAR T Cells in a Xenograft Tumour Model ... 27
Figure 3: Dose Escalation and Dose Expansions Phases ... 32
Figure 4: Overview of the Stages of the Study ... 33
Figure 5: CRS Management Overview ... 67

LIST OF ABBREVIATIONS

Abbreviation	Definition
AE	Adverse event
ALL	Acute lymphoblastic leukaemia
ALT	Alanine aminotransferase
APRIL	A proliferation inducing ligand
AST	Aspartate aminotransferase
ATIMP	Advanced therapy investigational medicinal product
BCMA	B cell maturation antigen
CAR	Chimeric antigen receptor
CD3, -19, -20, -28, -134	Cluster of differentiation 3, 19, 20, 28, 134
CPK	Creatine phosphokinase
CR	Complete response
CRF	Case Report Form
CRS	Cytokine release syndrome
CT	Computerised tomography
CTCAE	Common Terminology Criteria for Adverse Events
Cy/Flu	Cyclophosphamide and fludarabine
DLT	Dose limiting toxicity
ECG	Electrocardiogram
ECHO	Echocardiogram
ECOG	Eastern Cooperative Oncology Group
eCRF	Electronic Case Report Form
EDC	Electronic data capture
EU	European Union
EudraCT	European Clinical Trials Database
FDA	Food and Drug Administration
FLC	Free light chain
GCP	Good clinical practice
GMP	Good Manufacturing Practice
IB	Investigator’s Brochure
ICH	International Conference on Harmonisation
IDMC	Independent Data Monitoring Committee
IEC	Independent ethics committee
IFN	Interferon
Ig	Immunoglobulin
IHC	Immunohistochemistry
IL	Interleukin
IMiD	Immunomodulatory therapy
IMWG	International Myeloma Working Group
irAE	Immune-related adverse event
IRB	Institutional review board

Abbreviation	Definition
IV	Intravenous(ly)
M-protein	Monoclonal protein
MAD	Maximum administered dose
MM	Multiple myeloma
MR	Minor response
MRD	Minimal residual disease
MRI	Magnetic resonance imaging
mRNA	Messenger ribonucleic acid
MTD	Maximum tolerated dose
MUGA	Multiple gated acquisition
NCI	National Cancer Institute
NSG	NOD Scid Gamma
ORR	Overall response rate
OS	Overall survival
OX40	Also known as tumour necrosis factor receptor superfamily 4 [TNFRSF4] and cluster of differentiation 134 [CD134])
PBMCs	Peripheral blood mononuclear cells
PCR	Polymerase chain reaction
PET-CT	Positron emission tomography-computerised tomography
PFS	Progression-free survival
PR	Partial response
PTT	Partial thromboplastin time
qPCR	Quantitative polymerase chain reaction
RCR	Replication competent retrovirus
RNA	Ribonucleic acid
RP2D	Recommended Phase II dose
RQR8	Safety switch gene containing 2 copies of the rituximab mimotope flanking the QBEnd10 epitope on a CD8 stalk
SAE	Serious adverse event
sCR	Stringent complete response
SEC	Safety Evaluation Committee
SPEP	Serum protein electrophoresis
TACI	Transmembrane activator and calcium modulator and cyclophilin ligand interactor
TLS	Tumour lysis syndrome
TNF	Tumour necrosis factor
ULN	Upper limit of normal
UPEP	Urine protein electrophoresis
VGPR	Very good partial response

Lee L, *et al. J Immunother Cancer* 2023; 11:e006699. doi: 10.1136/jitc-2023-006699

1 INTRODUCTION

1.1 MULTIPLE MYELOMA

Multiple myeloma (MM) is a malignant disease of plasma cells that is responsible for approximately 10% of all haematological malignancies (Kyle and Rajkumar 2004). It occurs more frequently in elderly patients, with a median age of 67 years at presentation. Although some patients remain in long-term remission, the majority of patients relapse and die from the disease. Multiple myeloma is recognised as a source of significant morbidity and mortality. In 2012, approximately 114,000 patients per year are diagnosed with MM worldwide, with 39,000 new cases within the European Union (EU). Worldwide, 80,000 patients per year die due to the disease (CRUK 2014).

The progression to symptomatic MM involves genetic mutations in plasma cells, alterations in the bone marrow microenvironment, increased bone resorption, aberrant signalling-loops involving cytokines and more importantly, loss of immune surveillance (Kocoglu and Badros 2016). The proliferating MM cells displace the normal bone marrow, leading to dysfunction in normal haematopoietic tissue and destruction of the normal bone marrow architecture, which is reflected by clinical findings such as anaemia, paraprotein in serum or urine, and bone resorption seen as diffuse osteoporosis or lytic lesions shown in radiographs (Kyle et al. 2003). Patients with MM produce a monoclonal protein (M-protein), also called paraprotein (comprising M-protein and free-light chain), which is an immunoglobulin (Ig) or a fragment of one that has lost its function (Kyle and Rajkumar 2009, Palumbo and Anderson 2011). Normal Ig levels are compromised, leading to susceptibility to infections. Furthermore, hypercalcemia, renal insufficiency or failure, and neurological complications are frequently seen.

Treatment choices for MM vary with the aggressiveness of the disease and related prognostic factors. Newly diagnosed patients in good physical health with active disease will generally receive high-dose chemotherapy with autologous stem cell transplantation (Attal et al. 1996, Child et al. 2003). Patients in poor health receive chemotherapy (Harousseau and Moreau 2009). The therapeutic landscape of MM has changed markedly in the past decade, with the introduction of the novel immunomodulatory agents, thalidomide and lenalidomide, as well as proteasome inhibitor such as bortezomib (Laubach et al. 2009). New approaches to therapy that incorporate these agents have produced significantly higher response rates and improved duration of both progression-free survival (PFS) and overall survival (OS) in the context of randomised, controlled studies. Patients with disease that is refractory to both proteasome inhibitors and immunomodulatory drugs have poor prognoses; the estimated median OS is 9 months, and the estimated event-free survival is 5 months at best (Kumar et al. 2012, Laubach et al. 2014).

Recent new approaches involving selective elimination of the malignant clones through use of monoclonal antibodies such as daratumumab (Lonial et al. 2016) and elotuzumab (Jakubowiak et al. 2016), or stimulation of myeloma-specific immune responses through the use of vaccines (e.g. dendritic cell or peptide-based antibodies) have shown encouraging efficacy in heavily pre-treated and refractory patients with MM. Panobinostat, an oral pan-deacetylase inhibitor, in a Phase 3 study (San-Miguel et al. 2014) of panobinostat plus bortezomib and dexamethasone versus placebo plus bortezomib and dexamethasone, showed superior median PFS (11.99 months [95% confidence interval 10.33 to 12.94] vs 8.08 months [7.56 to 9.23]. Collectively, these novel therapies for MM have been associated with substantial improvements in patient outcome; however, it continues to be a fatal disease. This

Lee L, *et al. J Immunother Cancer* 2023; 11:e006699. doi: 10.1136/jitc-2023-006699

relapsed and refractory setting represents an area of unmet need for this serious and life-threatening disease.

1.2 B CELL MATURATION ANTIGEN AND TRANSMAMBRANE ACTIVATOR AND CALCIUM-MODULATOR AND CYCLOPHILIN LIGAND INTERACTOR BIOLOGY AND EXPRESSION IN MULTIPLE MYELOMA

B cell maturation antigen (BCMA) is a well-documented and clinically precedented target for MM. Expression of BCMA is restricted to the B cell lineage and it is widely expressed at increased levels on malignant plasma cells in MM (Claudio et al. 2002, Tarte et al. 2002, Tarte et al. 2003, Carpenter et al. 2013, Tai and Anderson 2015, Lee et al. 2016). The function of BCMA is to promote the survival of normal plasma cells and plasmablasts in response to binding its ligands - a proliferation inducing ligand (APRIL; a trimer with nanomolar affinity interaction) and B cell activating factor (a lower affinity interaction), regulating B cell maturation and differentiation into plasma cells. Currently an antibody-drug conjugate (NCT02064387 2014) and 3 chimeric antigen receptor (CAR) T cell products targeting BCMA (NCT02215967 2014, NCT02546167 2015, NCT02658929 2016) are in clinical trials for the treatment of MM.

Transmembrane activator and calcium modulator and cyclophilin ligand interactor (TACI) is also expressed on plasmablasts and plasma cells within the bone marrow as well as on MM plasma cells, although levels of expression on MM cells are lower and more variable than those of BCMA (Moreaux et al. 2005). It is also expressed on B cells, especially marginal zone B cells and cluster of differentiation (CD) 27+ memory B cells. Activation of TACI by its ligands, APRIL and B cell-activating factor, leads to B cell differentiation, upregulation of activation-induced cytidine deaminase messenger ribonucleic acid (mRNA), isotype switch, and maturation to T cell-independent antibody production (Garcia-Carmona et al. 2015). Chimeric antigen receptor T cell based targeting of BCMA and TACI could hold significant potential.

1.3 CLINICAL EXPERIENCE WITH CAR T THERAPIES IN MULTIPLE MYELOMA

Novel immunotherapies such as CAR T cell therapies hold promise for significant improvement in the overall outcome of MM. Autologous T cells can be genetically modified to express CARs, which are fusion proteins that include an antigen recognition moiety and T cell activation domains (Kershaw et al. 2005). Adoptive T cell approaches that utilise anti-CD19 CARs are in clinical development for the treatment of B-lineage malignancies and have demonstrated efficacy in clinical trials (Brentjens et al. 2003, Cooper et al. 2003, Hoyos et al. 2010, Jensen et al. 2010, Kochenderfer et al. 2010, Porter et al. 2011, Savoldo et al. 2011, Kochenderfer et al. 2012).

One clinical trial assessed CAR-modified T cells recognising CD138, highly expressed on MM cells, for the treatment of MM. Five patients received CAR-modified T cells recognising CD138 in an escalating dose. Efficient CAR-modified T cell expansion was detected in the peripheral blood and bone marrow. Four of the 5 patients had stable disease longer than 3 months, and 1 patient with advanced plasma cell leukaemia had a reduction of the peripheral myeloma cells.

Several ongoing clinical studies are utilising CARs targeting BCMA. Early results with this approach have shown encouraging results in the treatment of MM, including recent results

from a clinical study using a BCMA CAR (a single chain fragment variable recognising BCMA) (Ali et al. 2016). This study included 11 patients treated at 1 of 4 dose levels, 0.3×10^6, 1×10^6, 3×10^6, and 9×10^6 CAR T cells/kg. Toxicity among the 9 patients treated with the lowest 3 dose levels was mild and included cytopenias (attributable to chemotherapy), fever, and signs of cytokine release syndrome (CRS) including tachycardia and hypotension. More severe CRS was noted at the highest dose level. Two patients were treated with the highest dose level of 9×10^6 CAR T cells/kg. The first patient on this dose level had MM making up 90% of total bone marrow cells before treatment. Starting 4 hours after infusion of CAR T cells, 1 patient (Patient 10, second patient on highest dose) exhibited signs of CRS including fever, tachycardia, dyspnoea, acute kidney injury, coagulopathy, hypotension requiring vasopressor support, and muscle damage manifesting as an elevated creatine kinase level and weakness. His neutrophil count was less than 500/µL before the BCMA CAR T cell infusion and remained below 500/µL for 40 days after the CAR T cell infusion, before recovering. He also experienced prolonged thrombocytopenia. Patient 10's myeloma was rapidly eliminated after BCMA CAR T cell infusion. By immunohistochemistry (IHC) staining for CD138, bone marrow plasma cells were shown to have decreased from 90% before treatment to 0% 1 month after the CAR T cell infusion. Serum M-protein decreased from 1.6 g/dL before treatment to an undetectable level 2 months after treatment. Serum and urine immunofixation electrophoresis tests were negative 2 months after the CAR T cell infusion. Patient 10's myeloma relapsed at Week 17.

The second patient treated on the 9×10^6 CAR T cells/kg dose level had IgG lambda MM with 80% bone marrow plasma cells before treatment. Patient 11 experienced signs of CRS with toxicities including fever, tachycardia, hypotension, delirium, hypoxia, and coagulopathy. Patient 11's M-protein decreased from 3.6 g/dL before treatment to 0.8 g/dL 4 weeks after treatment. His serum lambda free light chain decreased from 95.9 mg/dL before treatment to 0.15 mg/dL 4 weeks after treatment. Four weeks after CAR T cell infusion, bone marrow plasma cells were undetectable and continued to be so at 28 weeks.

In another study CRB 401 (Berdeja et al. 2017), an anti-BCMA CAR-T was evaluated in 21 heavily pre-treated patients with MM at doses ranging from 50 to 800×10^6 CAR T cells. These patients were selected to have >50% expression of BCMA by IHC. The patients had received a median of 7 prior lines of therapy and all had progressed post autologous stem cell transplant. The overall response rate (ORR) was 100% for doses $>50 \times 10^6$ CAR T cells, with complete response (CR) rate of 56%. No dose limiting toxicities (DLTs) were reported up to a dose of 800×10^6 CAR T cells administered as a single dose. Cytopenias were very common and were attributed to lymphodepleting chemotherapy. Cytokine release syndrome (CRS) was noted in 71% of patients and was mostly mild, 2 patients reported Grade 3 CRS that resolved within 24 hours. No Grade 3/4 neurotoxicity was reported in this cohort of patients.

Another study (Fan et al. 2017) with an anti-BCMA CAR T in patients with relapsed/refractory MM, with at least 10% BCMA expression, reported ORR of 100% and sCR was seen in 14 of the 19 evaluable patients. The treatment was well tolerated with 83% of patients having symptoms of CRS, most of which were mild. Two patients had Grade 3 CRS managed by treatment. No neurotoxicity was reported. In general, the toxicities in patients receiving BCMA CAR T cells especially CRS were similar to toxicities in leukaemia patients treated with anti-CD19 CAR T cells.

There have been reports of antigen escape clones that have resulted in disease relapse in CD19 CAR studies, and similarly in early BCMA CAR studies (Ali et al. 2016). Additionally the CR of the patient in the BCMA CAR T study lasted only 17 weeks (Ali et al. 2016). This

Lee L, et al. J Immunother Cancer 2023; 11:e006699. doi: 10.1136/jitc-2023-006699

is possibly linked to the nature of current CAR therapies which only targets a single antigen. Therefore, there is a significant need for further improving the efficacy and safety of these therapies, including durable responses and inclusion of patients with low BCMA expression.

1.4 DESCRIPTION OF RQR8/APRIL CAR T CELLS: AUTO2

AUTO2 is a novel autologous enriched T cells retrovirally transduced to express a proliferation inducing ligand (APRIL) Chimeric Antigen Receptor (CAR) and the RQR8 safety switch (Figure 1). AUTO2 expressing APRIL targets 2 antigens, B cell maturation antigen (BCMA) and TACI. Instead of using a typical CAR construction of an antibody-based binding domain, AUTO2 includes an APRIL CAR construct that utilises the receptor-binding domain of APRIL (without the proteoglycan binding domain) to target BCMA and TACI. The APRIL CAR construct is a third generation CAR format that potentially results in maximal signal amplification. Furthermore, dual BCMA/TACI targeting may prevent escape by target antigen down-regulation as observed with CD19 targeting (Sotillo et al. 2015). AUTO2 therefore has the potential to be a potent CAR T cell therapy for MM.

The APRIL CAR consists of the receptor-binding domain of APRIL connected to the hinge region from Ig G1, which in turn is connected to the CD28 transmembrane domain. The endodomain is a composite of the endodomains of CD28, OX40 and CD3 zeta as illustrated in Figure 1. RQR8 is a compact safety switch gene that consists of 2 copies of a rituximab binding peptide, flanking a fragment of CD34, which binds the anti-CD34 antibody QBEnd10. This is attached to a CD8 stalk, transmembrane domain and anchor. RQR8 allows selective depletion of AUTO2 with the therapeutic monoclonal antibody rituximab, in the event of unmanageable toxicity (Philip et al. 2014).

Figure 1: Structure of the Constructs Expressed on AUTO2

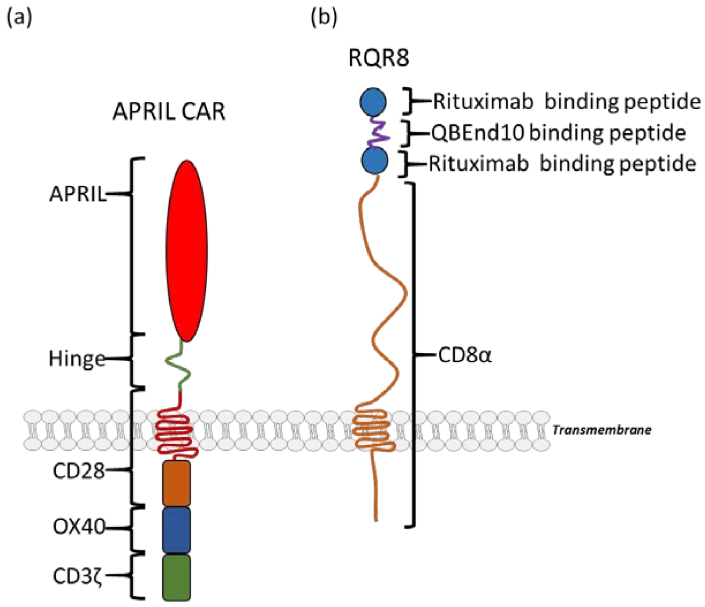

1.5 SUMMARY OF NONCLINICAL STUDIES

Further details regarding AUTO2 can be found in the Investigator's Brochure (IB).

1.5.1 *In Vitro* and *In Vivo* Pharmacology

Surface plasmon resonance was used to evaluate binding kinetics between the APRIL construct used in the RQR8/APRIL CAR and its receptor ligands, BCMA and TACI. Binding affinities of 3 nM and 5.6 nM were measured between APRIL and BCMA and APRIL and TACI respectively, similar to high affinity antibodies.

The cytolytic activity, *in vitro* proliferation and cytokine production of RQR8/APRIL CAR T cells when co-cultured with MM1.S or SupT1 cells was evaluated. MM1.S cells are a human cell line established from peripheral blood of a MM patient and express both BCMA and TACI, whereas SupT1 cells do not naturally express BMCA or TACI. Co-culture with RQR8/APRIL CAR T cells, but not an irrelevant CAR construct recognising disialoganglioside-2 (GD2), showed near complete abolition of MM1.S target cells (P<0.05) but not SupT1 cells at a 1:1 ratio for 48 hours. RQR8/APRIL CAR T cells demonstrated a strong proliferative response when co-cultured with MMI.S cells (86.9±1.9% (mean, standard error of the mean) of transduced cells showing at least 1 cell division) but not SupT1 cells (9.3±1.9%). Analysis of cytokines in cell supernatants revealed the production of the cytokines interleukin (IL)-2, interferon (IFN)-γ and granzyme B. Together, these data demonstrate a functional cytolytic/killing phenotype of RQR8/APRIL CAR T cells. The specificity of the RQR8/APRIL CAR T cells for BMCA or TACI was further evaluated by engineering SupT1 cells to express BCMA or TACI at a low density. RQR8/APRIL CAR T cells were co-cultured with engineered SupT1 cells and killing of target cells was assessed by flow cytometry. RQR8/APRIL CAR T cells do not kill wild-type SupT1 cells but effectively kill SupT1 cells engineered to express BCMA or TACI.

To demonstrate co-expression of RQR8 and APRIL CAR, transduced T cells were stained for RQR8 (using the anti-CD34 antibody, QBEnd10) and APRIL (using a polyclonal mouse anti-APRIL antibody). Results showed a strong positive correlation running broadly along the line of unity between RQR8 and APRIL expression, supporting the 1:1 co-expression of RQR8 and APRIL on transduced lymphocytes. The 'safety-switch' function of RQR8, when co-expressed with APRIL CAR was tested *in vitro* using a complement-mediated cytotoxicity assay. Addition of rituximab resulted in a near complete depletion of CAR T cells after 48 hours, with the remaining cells showing only a residual anti-tumour activity. Furthermore, the *in vivo* activity of RQR8 in an immunocompetent haploidentical adoptive transfer mouse model has been previously described (Philip et al. 2014).

RQR8/APRIL CAR was tested in an *in vivo* tumour xenograft mouse model. MM1. s-Fluc is a human MM cell line expressing both BCMA and TACI, transduced with firefly luciferase to allow detection of cells by bioluminescence, which when administered intravenously (IV) to immunocompromised (NOD scid gamma [NSG]) mice, results in a disseminated disease burden. NSG mice were injected with 5×10^5 MM1s.Fluc cells IV. After 13 days, bioluminescent imaging showed progressive disease with involvement of the skull, spine, and pelvis. Treated animals were given 5×10^6 RQR8/APRIL CAR T cells. Results show a rapid and complete response (CR) with myeloma becoming undetectable 4 days post-injection of CAR T cells (see Figure 2). In contrast, control mice showed further progression of tumour burden. Analysis of the bone marrow and spleen of mice treated with RQR8/APRIL reveal complete abrogation of tumour cells (P<0.01) compared to tumour-only controls (see Figure 2).

Lee L, *et al. J Immunother Cancer* 2023; 11:e006699. doi: 10.1136/jitc-2023-006699

Figure 2: Efficacy of RQR8-APRIL CAR T Cells in a Xenograft Tumour Model

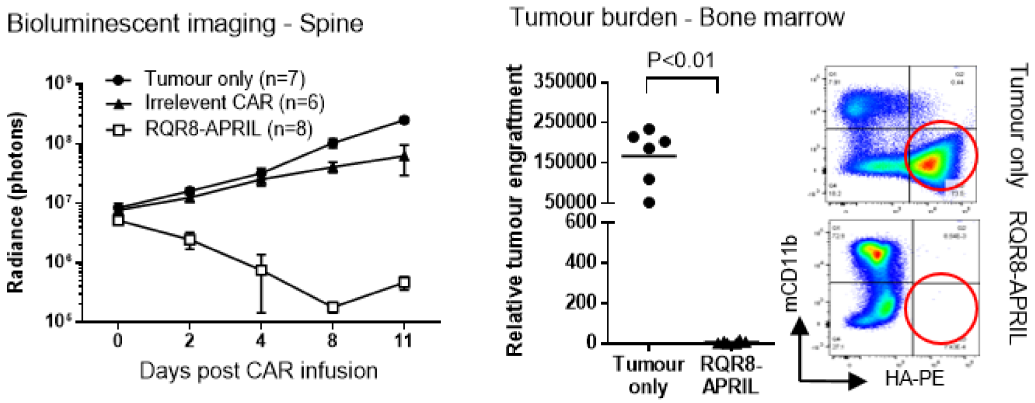

1.5.2 Toxicology

A proliferation inducing ligand can bind both human and murine BCMA/TACI at similar affinity (Kimberley et al. 2012) and efficient cytolysis is observed when APRIL CAR T cells are co-cultured with SupT1 cells engineered to express either human or murine BMCA/TACI. This cross-species reactivity enabled an efficacy study with toxicological endpoints using a xenograft mouse model of MM. The efficacy study is described above. For the toxicological assessment, mice were sacrificed on Day 24 and a complete necropsy examination performed which revealed no gross pathological abnormalities. Fifty-four tissue types were collected, preserved, and sections prepared for pathological assessment after haematoxylin and eosin staining. Histological examination of tissues revealed that there were no treatment-related histopathological findings observed in animals treated with RQR8-APRIL CAR T cells in any of the tissues examined.

Although BCMA is a clinically precedented target for both BCMA-targeted antibodies and CAR T cell therapy, TACI is a less well clinically characterised receptor. The mRNA expression of BCMA and TACI was measured in 72 human tissues by reverse transcription-polymerase chain reaction (PCR). Expressed genes were detectable at a low level in most tissues which likely reflects the presence of mononuclear blood cells expressing BCMA and/or TACI within the tissue vasculature. Distributions of the highest levels of mRNA for TACI and BCMA were broadly similar; in all tissues where TACI expression was demonstrated, BCMA was co-expressed. B cell maturation antigen gene expression were higher than TACI by approximately 5 to 10-fold in all tissues except in the spleen parenchyma, where expression of TACI and BCMA was at a similar level. The highest expression levels of both BCMA and TACI were in lymphoid tissues (spleen parenchyma and/or lymph gland/tonsil tissues). Notable expression levels were also seen in gastrointestinal tissues (caecum, colon, stomach, ileum, jejunum, duodenum and rectum) and in bronchial tissues (bronchus, lung parenchyma and tracheal) likely due to these being tissues where lymphoid tissue was present and B cells or plasma cells would normally reside. Low levels of BCMA expression, but not TACI, were noted in the testes and gall bladder. Similar profiles of BCMA expression were reported by (Carpenter et al. 2013) although this group further reported that protein expression of BMCA was restricted to plasma cells and was not detected in normal human tissues. In summary, these data suggest the risk of

on-target/off-tumour mediated toxicity is very low and therefore targeting both BCMA and TACI does not present a greater risk to patient safety than targeting BCMA alone.

A tissue-cross reactivity study using a labelled construct containing the APRIL-binding domain was technically unsuccessful (refer to the IB for more details) due to the complexity of creating a specific construct that is suitable for use in an IHC assay. However, data generated from the mRNA expression analysis, alongside the pathology data from the murine tumour model, provides assurance that targeting both BCMA and TACI is unlikely to present a greater risk of off-tumour toxicology than targeting BCMA alone.

1.6 SUMMARY OF CLINICAL STUDIES

The current study will be the first-in-human study of the advanced therapy investigational medicinal product (ATIMP), AUTO2, in patients with relapsed or refractory MM. There are limited data from the ongoing clinical studies, which are summarised below.

The first-in-man study AUTO2-MM1 has treated three patients as of 11 January 2018; they are disease evaluable and have completed the DLT observation period. All patients had relapsed MM, refractory to both proteasome inhibitor and immunomodulatory therapies (IMiDs), and received preconditioning chemotherapy with fludarabine and cyclophosphamide, as per protocol, prior to AUTO2 infusion.

The first patient (Patient 0201GB01001) treated in Cohort 1 received 15×10^6 RQR8/APRIL-CAR T cells, had maximal expansion at Day 21 (41 RQR8/APRIL CAR T cells/µL, 13.7% of total T cells). The patient had best response of stable disease as per International Myeloma Working Group (IMWG) response criteria at 1 and 3 months following AUTO2 treatment. Cohort 2 was started and the next two patients were treated with 75×10^6 RQR8/APRIL-CAR T cells. The first patient in this cohort (Patient 0201GB01002) had CAR T cells maximal expansion at Day 15 (5.54 RQR8/APRIL CAR T cells/µL, 2.14% of total T cells). Patient has stable disease as per IMWG response criteria at Month 1 post AUTO2 treatment. Subsequently, next patient (Patient 0201GB01003) had maximal expansion at Day 14 (33 RQR8/APRIL CAR T cells/µL, 5% of total T cells). At first disease assessment, the patient had stable disease but with increased serum paraprotein.

No infusion related issues were reported with the administration of preconditioning fludarabine and cyclophosphamide treatment or with AUTO2 treatment. No DLTs were reported for the three patients treated. As expected, bone marrow suppression was seen with pre-conditioning. The most frequent adverse event (AE) reported for the patients was neutrophil count decreased. Patient 0201GB01001 had prolongation of hospitalisation due to Grade 4 neutrophil count decreased, possibly related to AUTO2, and reported as a serious AE (SAE). This was not associated with infection and the patient received treatment with granulocyte colony stimulating factor. The patient had Grade 1 fever CRS that resolved with antipyretics. No neurotoxicity was reported.

There were no DLTs and the decision to open the next cohort (75×10^6 RQR8/APRIL CAR T cells) was made. Patient 0201GB01002 had no treatment-related SAEs reported. This patient had no AEs related to CRS or neurotoxicity. The patient had Grade 3 and 4 neutrophil count decreased, which was related to AUTO2, and the patient received granulocyte colony stimulating factor. Patient 0201GB01003 had no treatment related SAEs. Patient had Grade 1 CRS, which resolved, and was treated with a dose of tocilizumab. Patient had no AEs related to neurotoxicity.

Since this is a first study looking at dual BCMA/TACI it is noteworthy that no off-tumour toxicity with TACI has been reported to date. Patients receiving the 75×10^6 RQR8/APRIL CAR T cells had expansion of CAR T cells in peripheral blood, however they were undetectable by Day 28. The lack of a clinical response indicates that 75×10^6 RQR8/APRIL CAR T cells maybe a sub-therapeutic dose. In *CRB 401* (Berdeja et al. 2017) the active dose was considered to be at least 150×10^6 CAR T cells.

1.7 OVERALL RATIONALE FOR THE STUDY

The past decade has seen major progress in the understanding of the molecular oncogenesis of plasma cell neoplasms, which has significantly influenced the clinical management of these tumours. Despite these major advances, most cases of MM have remained incurable with the currently established therapy modalities. A considerable number of MM patients will ultimately experience a final tumour relapse without an additional, effective treatment option. Novel treatment concepts are therefore needed that address and overcome the limitations of current therapeutic modalities.

Novel immunotherapies such as CAR T cell therapies hold promise for significant improvement in the overall outcome of MM. Anti-CD19 CAR T cells in clinical development for the treatment of B-lineage malignancies have demonstrated efficacy in clinical trials. Several ongoing clinical studies are utilising CARs targeting BCMA. Early results with this approach have shown encouraging results in the treatment of MM. Unlike BCMA, CAR AUTO2 expressing APRIL can target 2 antigens expressed on myeloma cells, BCMA and TACI; enabling targeting of tumour cells expressing low antigen. Dual BCMA/TACI targeting may also prevent escape by target antigen down-regulation as observed with CD19 targeting. AUTO2 has also been engineered to be more active with the incorporation of both proliferation CD28 and survival OX40 co-stimulatory signals. Additionally, incorporation of RQR8 safety switch into the CAR adds to the overall safety of AUTO2. This first-in-human, Phase I/II study will assess the safety and preliminary activity of AUTO2 in patients with relapsed or refractory MM.

Lee L, *et al. J Immunother Cancer* 2023; 11:e006699. doi: 10.1136/jitc-2023-006699

2 STUDY OBJECTIVES AND ENDPOINTS

2.1 PRIMARY OBJECTIVES AND ENDPOINTS

Primary objectives and endpoints for Phase I are presented in Table 1.

Table 1: Primary Objectives and Endpoints for Phase I

Objectives	Endpoints
To assess the safety and tolerability of AUTO2 administration.	Incidence of Grade 3 to 5 toxicity occurring within the DLT period (28 days post AUTO2 infusion).
To identify the recommended Phase II dose (RP2D) and maximum tolerated dose (MTD), if an MTD exists, of AUTO2.	Frequency of DLT and the persistence of AUTO2.

AE=adverse event; DLT=dose limiting toxicity; MTD=maximum tolerated dose; RP2D=recommended Phase II dose; SAE=serious adverse event.

Primary objectives and endpoints for Phase II are presented in Table 2.

Table 2: Primary Objectives and Endpoints for Phase II

Objectives	Endpoints
To evaluate the anti-tumour effect of AUTO2	Best overall response post-AUTO2 infusion.
To assess the safety and tolerability of AUTO2 administration.	Frequency and severity of AEs and SAEs.

AE=adverse event; SAE=serious adverse event.

2.2 SECONDARY OBJECTIVES AND ENDPOINTS

Secondary objectives and endpoints are presented in Table 3.

Table 3: Secondary Objectives and Endpoints

Objectives	Endpoints
To evaluate the feasibility of generating the ATIMP, AUTO2.	Proportion of patients for whom an AUTO2 product can be generated (feasibility).
To evaluate the clinical efficacy of AUTO2	• Determine the clinical benefit (stringent complete response + complete response + very good partial response + partial response + minor response [sCR+CR+VGPR+PR+MR]) rate following treatment with AUTO2.<br>• To evaluate clinical outcomes including duration of response, time to disease progression, PFS and OS.
Biomarker and pharmacodynamic effects of AUTO2	Quantitative PCR (qPCR) and/or flow cytometry at a range of time points in the peripheral blood.

CR=complete response; MR=minor response; PR=partial response; qPCR=qualitative polymerase chain reaction; sCR=stringent complete response; VGPR=very good partial response.

Lee L, *et al. J Immunother Cancer* 2023; 11:e006699. doi: 10.1136/jitc-2023-006699

2.3 Exploratory Objectives

The exploratory objectives for this study are as follow:

- To assess Minimal Residual Disease following AUTO2 treatment.
- To characterise the CAR T cell immunophenotype pre- and post-infusion, and the relationship with dose of AUTO2 and severity of CRS.
- To assess the immune responses against AUTO2 i.e. presence or absence of human anti-CAR antibodies and related antibodies in the serum.
- Time course and magnitude of cytokine release evaluated using an appropriate assay.
- Depletion of BCMA+/TACI+ cell compartment as determined by flow cytometry and/or IHC on bone marrow samples.
- Presence of AUTO2 in bone marrow samples as determined by flow cytometry and/or IHC.
- Seek any relationship between parameters of activity and tumour burden (% plasma cells in bone marrow, or positron emission tomography-computerised tomography [PET-CT] assessment of disease) and level of BCMA/TACI expression (IHC or flow cytometry).
- Seek any relationship between incidence and severity of CRS and tumour burden (% plasma cells in BM, or PET-CT assessment of disease) and level of BCMA/TACI expression (IHC or flow cytometry).

3 STUDY OVERVIEW

3.1 STUDY DESIGN AND METHODOLOGY

3.1.1 Study Overview

This multi-centre, single-arm study will consist of 2 phases, a Phase I/dose escalation phase and a Phase II/expansion phase:

- Phase I: **Dose escalation** to identify the optimal dose of AUTO2 (doses from 15 × 10^6 to 450 × 10^6 RQR8-APRIL positive CAR T cells to be assessed).
- Phase II: **Dose expansion** to further assess safety and anti-tumour activity at the recommended dose identified in Phase II.

An overview of the study design is presented in Figure 3 below.

Figure 3: Dose Escalation and Dose Expansions Phases

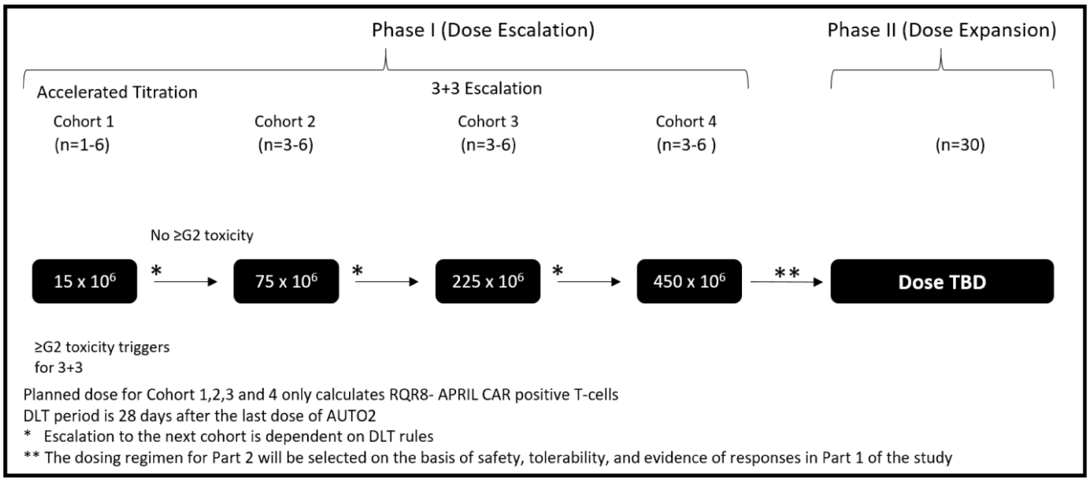

The study will consist of the following 5 stages:

- **Screening**: After providing written informed consent for study participation, all patients will be screened for study eligibility. Eligible patients will proceed to leukapheresis.
- **Leukapheresis**: Eligible patients will undergo leukapheresis to facilitate manufacture of the ATIMP, AUTO2. If sufficient quantity of the cells (prescribed dose ±20%) are produced, the patient will proceed to the Pre-Conditioning Phase.
- **Pre-conditioning**: If sufficient AUTO2 for the prescribed dose is successfully manufactured and the patients continue to meet eligibility requirements for the study, they will proceed to receive a lymphodepleting pre-conditioning treatment with cyclophosphamide and fludarabine for 3 days, timed to end 4 days before AUTO2 infusion.
- **Treatment**: AUTO2 for the prescribed dose will be administered IV as a single infusion on Day 0.). The treatment phase will extend from Day 0 (infusion day) until the end of the DLT observation period (28 days post last AUTO2 infusion).

Lee L, *et al*. *J Immunother Cancer* 2023; 11:e006699. doi: 10.1136/jitc-2023-006699

- **Follow-up**: The follow-up phase will begin after the treatment stage and end 2 years (±14 days) after infusion with AUTO2 or at disease progression or withdrawal of consent, which ever happens first (End of Study visit).

An overview of the 5 study stages is presented in Figure 4.

Figure 4: Overview of the Stages of the Study

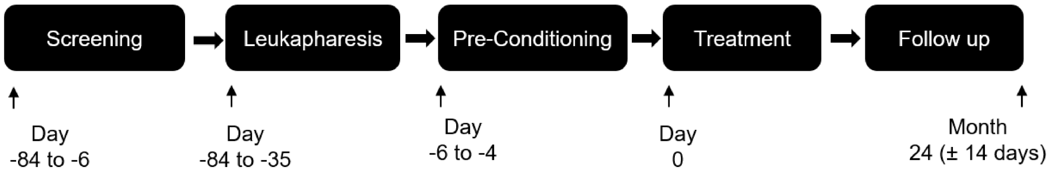

From signing of consent until the End of Study visit, patients will be closely monitored for AEs, laboratory abnormalities, CR and biomarker changes according to the schedule of assessments.

All patients will be enrolled into a long-term follow-up protocol (AUTO-LT1) at the End of Study visit and will be followed for safety evaluation and survival for 15 years from the first AUTO2 infusion or until death or withdrawal of consent, which ever happens first.

Note: Patients who have received AUTO2 and have discontinued or completed the study may continue to be monitored for AUTO2 treatment-related SAEs and AEs until they enrol onto the long-term follow-up study (AUTO-LT1) and the End of Study visit will be delayed.

3.1.2 Study Duration

The total study duration is estimated to be 4.5 years from first patient enrolled to the last patient, last visit (24-month visit). The end of the trial will be 24 months after the last patient has received AUTO2 infusion (or earlier if appropriate).

3.1.3 Phase I Dose Escalation

The dose escalation phase is designed to determine the RP2D of AUTO2 (based on total RQR8/APRIL positive CAR T cells) in patients with relapsed or refractory MM. Dose escalation will initially follow an accelerated dose titration design, in which a single patient will be dosed at the lowest dose level, followed by a 3+3 design (Figure 3). The trial will consist of up to 4 dose levels; eligible patients will be assigned to treatment groups sequentially. The inter-patient dosing interval for AUTO2 will be at least **14 days**, to allow for assessment of possible toxicity, the dosing interval between the 1st and 2nd AUTO2 naive patient in every cohort will be a minimum of 28 days. The Safety Evaluation Committee (SEC) may change the dosing interval based on emerging data. This window may be reduced to 1 week after a cohort has enrolled at least 3 patients and has been declared safe by the SEC. The DLT evaluation period will be **28 days** after the AUTO2 dose. Patients will be admitted to hospital for at least 10 days (or longer) for monitoring after receiving AUTO2 or until all AUTO2 related non-haematological toxicities have returned to ≤Grade 1 or baseline, or longer as clinically necessary.

3.1.3.1 Accelerated Titration

Dose escalation will follow an accelerated titration design and will start according to the following principles (Simon et al. 1997):

- A single patient will be treated with AUTO2 at the starting dose of 15×10^6 RQR8/APRIL positive CAR T cells.
- Accelerated titration will be stopped after the first occurrence of Grade 2 or higher toxicity that is considered probably or definitely related to AUTO2 during the DLT evaluation period. If the aforementioned events occur at the first dose level of 15×10^6 RQR8/APRIL positive CAR T cells, the study will transition to a 3+3 phase, where a minimum of 3 and maximum of 6 patients will be treated with the same dose.
- Based on emerging data (for example, 1st patient is considered to have low disease burden, <20% of bone marrow cells are plasma cells), the SEC may consider enrolling a second patient prior to dose escalation, even if the criteria for transition to 3+3 (as outlined above) have not been met.
- If the decision to dose escalate is made, then the second cohort will be opened at the dose level of 75×10^6 RQR8/APRIL positive CAR T cells.
- The study will transition to 3+3 phase at the second dose cohort, where a minimum of 3 and maximum of 6 patients will be treated with the same dose.

3.1.3.2 3+3 Dose Escalation

Once the criteria for stopping the accelerated dose titration phase have been met, or a dose level of 75×10^6 RQR8/APRIL positive CAR T cells has been reached (i.e. second dose level escalations), whichever comes first, dose escalation will transition to the 3+3 scheme (Table 4). Each dose level may enrol up to 6 patients. Evaluation of a dose level with at least 3 patients treated at the planned dose level completing the DLT evaluation period is required prior to escalation to the next dose level, except in cohort 1.

Based on emerging data, the SEC may decide whether it is safe to escalate the dose when fewer than three patients (but no fewer than two) have been treated in the 75 x 10^6 RQR8/APRIL positive CAR T cells (e.g. escalation from a dose level that may be considered sub therapeutic based on the emerging CAR T expansion, persistence and efficacy data); this may be done with a protocol amendment.

Patients treated at doses below the planned dose level will not be considered evaluable for making dose escalation decisions (additional patients will be enrolled to meet the minimum number needed to make the dose escalation decision). Similarly, if a patient is treated at the higher dose before it is declared safe (lower dose considered sub therapeutic) then that patient will not be evaluable for dose escalation decision making (will be considered evaluable for dose escalation decision making if they develop a DLT with retreatment). However, dose escalation decisions will take into account all available data, including biomarker data and the safety profile of all patients treated in prior dose levels.

The SEC will meet after the first patient in every cohort completes the DLT assessment period, to confirm continuation of enrolment to that cohort and thereafter meet again after the third patient in a cohort has completed the DLT assessment period. Only once a cohort is declared safe by the SEC can the next higher dose level be opened. Back filling of a cohort (maximum n=6 evaluable patients/cohort) declared safe may be undertaken in parallel to the ongoing enrolment of a higher dose level, to obtain additional safety and efficacy data in

patients who are unable to wait for the required inter-patient dosing interval due to risk of clinical deterioration. A minimum dosing interval of 7 days will be maintained between patients enrolled to a cohort for back filling purposes as well as for those being re-treated as per Section 7.3.6.

Dose escalation will continue until the planned maximum administered dose (MAD) of 450×10^6 RQR8/APRIL positive CAR T cells is reached or lower if patients experience adverse effects (Table 4) that are presumed to be related to AUTO2 and are considered unacceptable due to severity and/or irreversibility. Intermediate doses may be explored based on emerging data, however it will not exceed the current maximum suggested dose of 450×10^6 RQR8/APRIL positive CAR T cells without a protocol amendment.

Recent safety data from two anti-BCMA CAR T studies (Berdeja et al. 2017, Fan et al. 2017) has shown mostly mild CRS and no Grade 3 or 4 neurotoxicity at various escalating doses. Notably the CRB-401 study (Berdeja et al. 2017) evaluated 4 dose cohorts: 50, 150, 450 and 800×10^6 CAR-T cells given as a single dose. No DLTs were observed in these cohorts and treatment was well tolerated up to 800×10^6 CAR T cells. Seventy one percent of patients had CRS most of which was mild (Grade 1 or 2), 2 patients had Grade 3 CRS that resolved within 24 hours. This study has initiated expansion phase evaluating the active doses from 150 to 450×10^6 CAR-T cells.

Given the safety profile and reported data from other anti-BCMA CAR-T studies, the dose cohorts in this study of up to 450×10^6 cells are within range of what has been evaluated and administered safely as a single dose. Based on emerging safety data, if a split dose regimen is considered appropriate a protocol amendment will be submitted to implement it.

Table 4: 3+3 Dose Escalation Decision Rules

Number of Patients with DLT at a Given Dose Level	Escalation Decision Rule
0 out of 3	Escalate to the next dose level
1 out of 3	Enrol 3 additional patients at the current dose level for a total of 6 patients.
1 out of 6	Escalate to the next dose level with a dose increase of ≤100% of the next planned dose level.
2 or more patients in a dosing cohort (up to 6 patients)	The MTD has been exceeded. Either:<br>• May evaluate an intermediate dose lower than the current dose.<br>• Expand a prior cohort up to 6 patients.

DLT=dose limiting toxicity; MTD=maximum tolerated dose.
Note: The Investigators may override these guidelines if there are particular safety issues, for which moving to a higher dose is not considered appropriate.

The dose escalation decisions will be made by the SEC (Section 10.5) and the study could be stopped by the Independent Data Monitoring Committee (IDMC) or SEC upon occurrence of any of the events described in Section 10.4.

3.1.4 Dose Limiting Toxicity

Toxicities will be graded for severity according to the National Cancer Institute Common (NCI) Terminology Criteria for Adverse Events (CTCAE), Version 4.03 and the CRS will be graded as per (Lee et al. 2014). The DLT evaluation period will end 28 days after the AUTO2 dose. The DLT criteria take into consideration the single dose nature of AUTO2 treatment (unlike repeat dose treatments of usual anti-cancer agents) and the potential for differential

expansion of CARs post infusion in different patients. As well as features inherent to CAR therapy, transient fever due to low grade CRS, and in the setting of pre-conditioning, induced cytopenias/neutropenia are seen in most patients. These are not necessarily classical neutropenic fevers related to infection. The CRB 401 study with anti-BCMA CAR shows 86% patients had Grade 3/4 neutropenia that was related to preconditioning and lasted for several weeks after infusion. None of these patients had a DLT. Similarly, both anti-CD19 CAR-T therapies axicabtagene ciloleucel and tisagenlecleucel have prolonged cytopenias after infusion related to lymphodeleting therapy (FDA 2017, FDA Undated), 40% of patients treated with tisagenlecleucel had Grade3/4 neutropenia lasting more than 28 days after treatment. Another anti-CD19 CAR, JCAR017 (NCT03310619) study showed the majority of patients had Grade 4 neutropenia.

Dose limiting toxicity will be defined as:

- Any new non-haematological AE of Grade 3 or higher toxicity using the NCI CTCAE (Version 4.03), which is probably or definitely related to AUTO2 therapy, which occurs within the DLT evaluation period, and which fails to resolve to Grade 2 or better within 14 days, despite appropriate supportive measures.
- A Grade 4 CRS.
- Any other reason for activation of the safety switch after receiving AUTO2.
- Any other fatal event (Grade 5) or life-threatening event (Grade 4) that cannot be managed with conventional supportive measures or which in the opinion of the SEC necessitates dose reduction or other modification to trial treatment to avoid a similar hazard in future patients. Effort should be made to perform an autopsy in case of fatal event where the aetiology is unclear.
- Any event that in the opinion of treating investigators and/or Medical Monitor puts the patient at undue risk may also be considered a DLT.

Reporting Requirements for DLT

All DLTs must be reported to the Sponsor as SAEs within 24 hours of site staff becoming aware of them (see Section 10.1.5). All DLTs will be notified to the SEC by the Sponsor.

Although not necessarily considered a DLT, a decision to activate the safety switch by administration of rituximab at any time should be reported as an SAE to the Sponsor. The SEC will also be informed by the Sponsor (see Section 10.1.3).

Maximum Tolerated Dose: The MTD is defined as the highest dose level of AUTO2 at which ≤1 patient out of 6 patients experience a DLT during the DLT evaluation period. If 2 or more out of 6 patients at a dose level experience a DLT during the DLT evaluation period, the MTD has been exceeded. If the MTD is exceeded due to a specific toxicity that can be managed with supportive care, an additional 3 patients may be enrolled at the dose level that exceeded the MTD with establishment of supportive care measures. A summary of available safety data and a description of the plans for supportive care measures with further enrolment at that dose level will be provided to Independent Ethics Committees/Institutional Review Boards (IECs/IRBs) prior to dosing.

Maximum Administered Dose: The planned MAD for this study is 450×10^6 RQR8/APRIL positive CAR T cells in case the MTD is not defined. The MAD may be lower based on emerging data.

Lee L, *et al. J Immunother Cancer* 2023; 11:e006699. doi: 10.1136/jitc-2023-006699

Recommended Phase II Dose: The RP2D is either identical to the MTD or MAD or a lower dose level AUTO2 is selected on the basis of a cumulative review of safety, persistence of the CAR T cells and clinical activity. The RP2D dose level may be expanded up to 6 patients to further characterise safety.

3.1.5 Planned Dose Levels

The 4 planned dose cohorts are presented in Table 5.

Table 5: Treatment Cohorts

Treatment Cohorts	RQR8/APRIL CAR Positive T Cell Dose 1 - Day 0
Cohort 1	15×10^6
Cohort 2	75×10^6
Cohort 3	225×10^6
Cohort 4	450×10^6

APRIL=a proliferation inducing ligand; CAR=chimeric antigen receptor; RQR8=safety switch gene.

Patients weighing less than 50 kg will have a 25% reduction in total dose, patients weighing more than 100 kg may receive a 25% increase in total dose.

These dose levels were selected as they provide an optimum range for assessing safety, CAR T cell persistence and anti-tumour activity. This range is within the dose levels assessed in other CAR T studies. Based on emerging data, intermediate dose levels may also be explored.

On occasion, RQR8/APRIL CAR positive T cell production may fail to generate sufficient cells for the current dose level. In this case, the patient can be treated on study but at the dose level achieved during manufacture. If a production fails to generate sufficient RQR8/APRIL CAR positive T cells for Cohort 1, the patient cannot be treated on study.

Note: The dose determination is based solely upon the transduced cells (i.e. RQR8/APRIL CAR positive T cells). The non-transduced cells in the drug product will not be counted towards determining the dose. A patient may be eligible for dosing if the dose is within 20% of the prescribed dose (±20%).

3.1.6 Phase II Dose Expansion

Once the RP2D is determined, the Phase II dose expansion part of the study will open. This part will enrol up to 30 patients. There is no required inter-patient dosing interval.

3.2 RATIONALES FOR STUDY DESIGN, DOSE AND POPULATION

3.2.1 Study Design Rationale

This single-arm dose-escalation Phase I/II study, is designed to assess safety, tolerability and optimum RP2D and MTD, if a MTD exists. Due to the novelty of targeting TACI, the design encompasses an accelerated phase allowing for low starting dose whilst exposing a minimum number of patients to a potential sub-therapeutic dose, should there be no significant off-target toxicity. A minimum dosing interval has been incorporated to reduce the risk of inducing severe adverse effects in more than 1 patient. There will be a 28-day observation period between the 1st and 2nd patient in every cohort and at least 14 days between subsequent

Supplemental material BMJ Publishing Group Limited (BMJ) disclaims all liability and responsibility arising from any reliance placed on this supplemental material which has been supplied by the author(s) J Immunother Cancer

patients until the dose is declared safe. The rationale for the dosing interval is based on the anticipated peak expansion of the infused cells that is likely to happen within the 1st 14 days.

Following the accelerated phase, the subsequent escalation is based on the standard 3+3 format and is considered reasonable for these cohorts. This design would allow for dosing an adequate number of patients prior to evaluation of safety and biological activity such as serum cytokine levels and CAR T cell persistence. However, based on emerging data, if a dose cohort is considered safe but has poor expansion, persistence or clinical response, then the SEC can recommend escalation to the next dose level to minimise patients being dosed at a sub-therapeutic dose. The dose may be escalated after SEC endorsement and a protocol amendment. Additionally, should emerging safety data indicate that splitting the total dose is likely to be safer and effective then this will be implemented after a protocol amendment The current experience from *CRB 401* (Berdeja et al. 2017) and other BCMA CAR studies suggest that single dose is likely to be safe and effective. The proposed design incorporates assessing a range of doses from 15×10^6 to 450×10^6 CAR T cells. This would allow for de-risking any potential for off target binding of TACI at the lower doses and still allow safely escalating the dose to a range that has been found to be safe and active in BCMA CAR T cell studies (Ali et al. 2016, Berdeja et al. 2017, Fan et al. 2017).

The Phase II/expansion part of the study is based on a Simon's 2-stage optimal design; the rationale for dosing up to 30 patients is to detect early signs of efficacy in addition to generating additional safety data at the RP2D.

3.2.2 Study Population Rationale

AUTO2 is designed to specifically bind to BCMA or TACI, which are only expressed on plasma cells. The study therefore will restrict the patient population to the plasma cell malignancy MM. In addition, since this is an experimental therapy, the study patient population will be restricted to only those with confirmed diagnosis of MM as per the IMWG, that have relapse or intolerance after exposure to alkylator therapy, immunomodulatory drugs and proteasome inhibitor. Patients included in this study would have received at least 3 prior lines of myeloma therapy or double refractory to IMiD and proteasome inhibitor. Moreover, only patients whose leukapheresis sample has been successful in generating AUTO2 in adequate quantity will be treated (or undergo pre-conditioning), as this is a pre-requisite for AUTO2 therapy.

3.2.3 Cyclophosphamide and Fludarabine Pre-conditioning Rationale

Pre-conditioning strategies that deplete host lymphocytes have been shown to enhance clinical responses to some adoptive T cell therapies (Muranski et al. 2006, Spear et al. 2013). Lymphodepletion prior to adoptive transfer of tumour specific T-lymphocytes is thought to enhance treatment efficacy by eliminating regulatory T cells and increasing access of the transfused CAR T cells to activating cytokines (Klebanoff et al. 2005, Wrzesinski and Restifo 2005). Cyclophosphamide has an established history in lymphodepleting regimens used prior to adoptive cell immunotherapy (Sporn et al. 1993, Curti et al. 1998, Brentjens et al. 2011, Chu et al. 2012). It is used alone or often used in combination with other agents (Dudley et al. 2008, Laurent et al. 2010, Geller et al. 2011). Fludarabine, either as a single agent or in combination with other cytotoxic agents, has been used as a lymphodepleting preparative regimen to reduce transplant-related toxicities and allow stem cell transplantation in elderly and medically infirm patients, as well as in adoptive T cell therapies, including CAR T cell therapy (Louis et al. 2011). Fludarabine is also commonly used in combination with cyclophosphamide (and rituximab) in the treatment of patients with chronic lymphocytic

Lee L, et al. J Immunother Cancer 2023; 11:e006699. doi: 10.1136/jitc-2023-006699

leukaemia (CLL) (Hallek 2013). Here-in its given every 4 weeks for multiple cycles compared to a single cycle therapy used for pre-conditioning.

Cyclophosphamide and fludarabine based pre-conditioning have become the preferred regimen for CAR T therapies and have been used in multiple studies (Kochenderfer et al. 2015, Lee et al. 2015, Ali et al. 2016)). The combination is also considered to be superior to cyclophosphamide alone. In an anti-CD19 CAR T cell study (JCAR014) patients with acute lymphoblastic leukaemia (ALL) who received cyclophosphamide and fludarabine (Cy/Flu) based lymphodepletion were compared with 1-third who received cyclophosphamide without fludarabine. At 18 months, a disease-free survival rate of approximately 60% was seen in the Cy/Flu ALL group compared with <10% of the cyclophosphamide-only arm ($p = 0.0005$) (Turtle et al. 2016). Additionally, in Non-Hodgkin lymphoma, the objective response rate for patients treated with Cy/Flu and CAR-modified T cells at the MTD ($n = 19$; 2×10^6/kg) was 84% (47% CR). Chimeric antigen receptor T cell expansion and persistence, and OS and PFS were better in Cy/Flu patients compared to those who received cyclophosphamide alone (objective response rate 50%, CR 8%, $n = 12$).

Cyclophosphamide administered alone is given at a high dose (2 to 4 gm/m^2). However, since the purpose of pre-conditioning chemotherapy is lymphodepletion and not myelosuppression, higher doses of cyclophosphamide (2 to 3 gm/m^2 × 1 to 2 days) is probably unnecessary, especially when given with fludarabine (Turtle et al. 2016). A lower dose of cyclophosphamide (300 mg/m^2 × 3 days or 900 mg/m^2 × 1) is likely to be adequate as this regimen has been evaluated in multiple clinical studies and considered relatively safe (Lee et al. 2015) and active (Ali et al. 2016, Neelapu et al. 2016).

Fludarabine like all purine analogues is neurotoxic, and neurotoxicity was commonly observed in the 1980s while the therapeutic window was being established. At "standard" doses of 25 to 30 mg/m^2, neurotoxicity is rarely seen. However, severe neurotoxicity leading to death was reported recently in the JCAR015 ROCKET trial (NCT02535364 2016) that used a fludarabine based conditioning regimen.

On 07 July 2016, the Food and Drug Administration (FDA) had suspended recruitment into the ROCKET trial following the deaths of 3 young adult ALL patients due to cerebral oedema. The 3 deaths occurred amongst approximately 7 patients treated with Cy/Flu conditioning prior to JCAR15 treatment. Previous patients in the trial had been conditioned with cyclophosphamide alone (approximately 14 patients; 20 patients recruited to the trial in total) with no deaths or unexpected toxicity. The Sponsor's report mentions "cerebral oedema", although no detail of the neurological syndrome is yet available. Most studies have described the neurotoxicity associated with CD19 targeting as a leukoencephalopathic picture. In the absence of post mortem data, there is little sound information about the pathophysiology of this process. The FDA lifted the recruitment hold a week later (13 July 2016) following agreement to return to conditioning with cyclophosphamide alone in this trial. No other trials (including similar trials using Cy/Flu conditioning) were affected. A subsequent announcement on the 14 July 2016 indicated that there are now 4 confirmed deaths from cerebral oedema in the ROCKET trial (NCT02535364 2016) attributable to therapy. Of note, it has been reported that the ZUMA-1 trial (NCT02348216 2016) had successfully completed recruitment of 72 patients to its trial of CD19-targeted therapy (using KTE-CD19), in which Cy/Flu conditioning was used but at lower doses than in the ROCKET trial, with no neurotoxicity-related deaths. There have been 50 patients on the NCI studies with no deaths due to neurotoxicity. In all of the studies conducted at the University of Pennsylvania using Cy/Flu and a CD19-41BB-zeta CAR in the last 3 years, only 1 neurotoxicity death has been reported.

There is overwhelming evidence that the toxicity is attributable to the enhanced conditioning of fludarabine potentiating the CD19 CAR T cells, which affect an immunological neurotoxicity dependent on the anti-CD19 single chain fragment variable component of the CAR, rather than being a direct effect of fludarabine.

- Neurotoxicity is a feature of all CD19 CAR studies and of bi-specific T cell engagers (Goebeler et al. 2016) which target CD19 (e.g. blinatumomab). Approximately 30 to 40% of patients with CD19 CAR T cell engraftment report some form of neurotoxicity. Neurotoxicity is the most challenging clinical problem associated with CD19 CAR since it does not easily correlate with T cell dose, underlying disease or the neurological involvement of leukaemia/lymphoma or CRS.
- This toxicity does not seem to occur with other CAR therapies where T cells engraft for instance against CD22 and BCMA (Fry et al. 2015, Ali et al. 2016) although there have been fewer patients treated. One theory is that CD19 or CD19-like protein is expressed on glial cells.
- The ZUMA-1 study used similar T cell doses. The receptor is also anti-CD19/28-Z, but containing a different single chain fragment variable and reports neurotoxicity including a few severe cases but no deaths. This study employed a lower dose/intensity of pre-conditioning (Kochenderfer et al. 2016, Neelapu et al. 2016) with cyclophosphamide (300 to 500 mg/m^2 × 3 days or 900mg/m^2 × 1) and fludarabine (30 mg/m^2 × 3 days) compared to the ROCKET study's 1 to 3 gm/m^2 cyclophosphamide + 25 to 30 mg/m^2 of fludarabine × 5 days which is repeated 2 to 4 weeks later. Additionally, a short washout period of only 1 day after pre-conditioning may also have contributed to this toxicity.
- The most likely explanation of the ROCKET trial deaths is that the addition of fludarabine potentiated the CD19 CAR activity and resulted in CD19 CAR mediated neurotoxicity. It is possible that the higher dose of fludarabine (additionally giving 2 cycles of it) used in the ROCKET trial combined with the specific CD19 CAR (JCAR015) are a combination particularly prone to causing neurotoxicity.

Cyclophosphamide administered alone is given at a high dose (2 to 4 gm/m^2) and could result in severe myelosuppression. However, since the purpose of pre-conditioning chemotherapy is lymphodepletion and not myelosuppression, higher doses of cyclophosphamide are probably unnecessary, especially when given with fludarabine (Turtle et al. 2016). A lower dose of cyclophosphamide (300 mg/m^2 × 3 days or 900 mg/m^2 × 1) is likely to be adequate as this regimen has been evaluated in multiple clinical studies and considered relatively safe (Lee et al. 2015) and active (Ali et al. 2016, Fry 2016, Kochenderfer et al. 2016, Neelapu et al. 2016).

In the current study, we propose to start at a very low initial dose of 15 × 10^6 RQR8/APRIL CAR Positive T Cells (approximately 2 × 10^5 cells/kg) combined with the cyclophosphamide/fludarabine based pre-conditioning regimen and titrate the dose up based on emerging safety data. In addition, we plan to administer a low dose of both cyclophosphamide (300 mg/m^2 × 3 days) and fludarabine (30 mg/m^2 × 3 days), followed by a 4-day washout period prior to infusion of AUTO2 which has taken into consideration the half-life of fludarabine of 20 hours. This would result in elimination of over 4 half-lives of fludarabine and will potentially decrease any cumulative toxicity with AUTO2. A longer washout period is unlikely to add any further benefit and is likely to be suboptimal as the cytokine surge, especially the IL-15 surge, observed post-conditioning chemotherapy with Cy/Flu happens in the first few days and returns to baseline around 10 days later (Rossi et al. 2015). It is probably ideal to dose the CAR T cells early within this window as IL-15 is

necessary for CAR T cell expansion and resulting efficacy (Xu et al. 2014). In an anti-CAR 19 study in refractory diffuse large B cell lymphoma, patients obtaining CR or PR had higher peak levels of IL-15 and IL-10 than patients with stable disease or progressive disease (Kochenderfer et al. 2016). Considering the nature of the patients being treated and the fact that pre-conditioning with Cy/Flu has been shown to increase clinical activity, we believe the proposed dose and regimen is justified.

3.2.4 Starting Dose Rationale

The primary consideration for selection of a starting dose for the proposed trial is the safety of the patient. Chimeric antigen receptor T cell dosing is a challenging area and the standard methods for dose determination based on pre-clinical data are not transposable for cell therapies. Chimeric antigen receptor T cells expand *in vivo*, with expansion differing from one patient to another. Consequently, efficacy and toxicity cannot easily be correlated with the starting dose administered. Therefore, the choice of the starting dose has been based mainly on CAR-related clinical trial literature.

For CAR T therapies, there is no clear correlation with dose given and either efficacy or toxicity, although there is a suggestion from 1 CD19 CAR study in children and young adults that 10^6 CAR T cells/kg is the MTD (Lee et al. 2016) this could vary based on the disease burden and degree of expansion of the infused CAR T cells. In another study in adult patients with ALL, a dose of 2×10^7/kg was considered unacceptable and 2×10^6/kg was considered to manageable, a further recommendation was to administer this dose to patients with 20% or less BM blasts to further reduce severe CRS (Turtle et al. 2016) Based upon published literature and recent presentations/discussions in the field of CD19 CAR T cell therapies, the minimum effective therapeutic dose is considered to be around 10^6 CAR T cells/kg. The MTD is also likely to vary based on the CAR T cell construct and the target antigen expression, lower antigen expression is likely to require higher dose of cells to see biological activity.

B cell maturation antigen and TACI expression on MM cells is significantly less compared to CD19 expression on B-cells. Recently, the interim results of a NCI clinical study using a BCMA CAR (a single chain fragment variable recognising BCMA) in MM patients have been published (Ali et al. 2016). Eleven patients were treated at 1 of 4 dose levels, 0.3×10^6, 1×10^6, 3×10^6, and 9×10^6 CAR T cells/kg. Toxicity among the 9 patients treated with the lowest 3 dose levels was mild and included cytopenias (attributable to chemotherapy), fever, and signs of CRS (including tachycardia and hypotension). More severe CRS was noted at the highest dose level which was considered to be at or near MTD. The recommendation was to administer this dose (9×10^6 CAR T cells/kg) only to patients with less than 50% bone marrow plasma cells.

The proposed starting dose of 15×10^6 RQR8/APRIL CAR positive T cells (fixed dose) corresponding to a dose of 2×10^5 RQR8/APRIL CAR positive T cells/kg based on an average person weight of 75 kg, is considered a safe starting dose as it is at the lower end of starting doses in CD19 CAR T clinical trials (refer to the IB) and lower than the 10^6 CAR T cells/kg suggested to be an effective clinical dose. It is slightly lower than the starting dose in the BCMA CAR T cell clinical trial and 45-fold lower than doses at which severe CRS was observed. Although it is possible that this dose level is sub-therapeutic based on the BCMA CAR trial, efficacy has been observed at similar doses in some patients in CD19 trials. Furthermore, Autolus believes that AUTO2 may be more potent than the NCI BCMA CAR (Ali et al. 2016), as it is designed to trimerise on binding BCMA/TACI. Trimerisation may contribute to activity at low antigen density but does not increase maximal activity.

Additionally, early clinical data from testing of a 3rd generation CD19 CAR does not show increased CRS despite doses of 2×10^8 CAR T cells/m^2 (equivalent approximately to a flat dose of 1×10^8 (Enblad et al. 2015). It seems less likely hence that 3rd generation CARs will intrinsically have significantly greater propensity for toxicity. Since there is less precedence for the use of CAR T cells in MM than there is in other haematological malignancies we have chosen a conservative starting dose which is based on a body of clinical experience of CAR T cell therapy. Moreover, with the proposed study design, only 1 or 2 patients may be exposed to this dose level.

3.2.5 Dosing and Assessment Rationale

Patients will be treated with AUTO2 once at doses that are likely to be safe and efficacious. Most CAR T therapies are generally administered once and thereafter undergo expansion *in vivo* upon contact with the target antigen expressed on tumour cells, resulting in significant anticancer activity in most patients. It is anticipated that AUTO2 will have similar expansion *in vivo*, rendering the need for re-dosing un-necessary. On occasion, a re-treatment dose may be given if the patient meets the criteria for re-treatment (Section 7.3.6). Subsequent to treatment with AUTO2, safety will be monitored closely (both in hospital and as an outpatient) and efficacy will be assessed periodically as described in the Schedule of Assessments. The timing of assessments is designed to capture early signs of toxicity and efficacy.

3.2.6 Biomarker Collection and Assessment Rationale

Current experience with CAR T cell therapies indicate that the safety depends on the disease burden, CAR T proliferation, cytokine release and efficacy depends on CAR T proliferation and persistence. To this end, the current biomarker strategy is designed to assess blood cytokine levels and CAR T levels and persistence at various time points after AUTO2 infusion. In addition, bone marrow sampling will be performed to assess disease status and expression of BCMA/TACI on the plasma cells, which would help evaluate efficacy as well as resistance. Additional optional samples may be collected to assess cyclophosphamide and/or fludarabine drug levels.

3.3 RISKS AND MITIGATION STRATEGY

This exploratory study is designed to assess the safety and biological activity of different doses of AUTO2 in a limited number of patients. Given the treatment novelty, this study will involve patients whose disease has progressed after treatment with the main approved classes of anti-myeloma agents. Such patients are considered to have poor prognosis and are candidates for promising experimental therapies.

Leukapheresis: This is necessary for enrolling onto this study as it is a prerequisite for preparing AUTO2. Cell production efficiency of at least 80% is anticipated and is dependent on the quality of the leukapheresate. AUTO2 infusion may or may not provide clinical benefit to these patients. Risks of leukapheresis are summarised in Table 6.

Table 6: Leukapheresis – Risks and Mitigation Strategy

Risks	Mitigation Strategy
**Pain and bruising** due to blood draw.	Experienced people performing the procedure, analgesics to be used as needed for pain
**Bacterial bloodstream infections** associated with the insertion of access and return venous access devices.	The procedure will be carried out by trained and experienced personnel and risks will be minimised by strict adherence to aseptic measure.
**Anticoagulant Citrate Dextrose Solution, Solution A toxicity** is a recognised complication as blood that comes into contact with the artificial surfaces of the extracorporeal tubing set must be anticoagulated to prevent clotting.	Patients will be monitored for symptoms of hypocalcaemia, the rate of citrate infusion to the patient and duration of the procedure will be controlled by experienced personnel.

Pre-conditioning Chemotherapy: Lymphodepletion with cyclophosphamide and fludarabine prior to adoptive transfer of tumour specific T cells is thought to enhance treatment efficacy by eliminating regulatory T cells and increasing access of the transfused CAR T cells to activating cytokines. Pre-conditioning is expected to increase the survival of AUTO2 and therefore the chance of anti-tumour efficacy. Pre-conditioning will only be undertaken after confirmation of the successful production of AUTO2. Risks of pre-conditioning with chemotherapy are summarised in Table 7.

Table 7: Pre-conditioning Chemotherapy – Risks and Mitigation Strategy

Risks	Mitigation Strategy
**Myelosuppression** resulting in anaemia, thrombocytopenia and lymphopenia, are the most common toxicities. Moderate to severe myelosuppression is possible. Nadir for granulocyte is 1 to 2 weeks and platelets 2 to 4 weeks after chemotherapy, with recovery usually within 4 to 6 weeks. Neutropenic fever, infections, sepsis and septic shock may occur and may sometimes be fatal.	The fludarabine and cyclophosphamide chemotherapy given is milder compared to general chemotherapy received by patients and will be given only once (1 cycle, given over 3 days).
**Cyclophosphamide** associated toxicities, including but not limited to haemorrhagic cystitis, pyelitis, myocarditis and myopericarditis, pneumonitis and pulmonary fibrosis, veno-occlusive liver disease (per approved label) may also occur.	Considering the low dose and short duration of treatment, these toxicities are less likely. Toxicities will be managed as per standard institutional policy and by trained personnel.
**Fludarabine** is generally well tolerated: the most common side effects are lymphopenia and infection. Serious, and sometimes fatal infections, including opportunistic infections and reactivations of latent viral infections such as Herpes zoster, Epstein-Barr virus and progressive multifocal leukoencephalopathy, have been reported in patients treated with higher doses and for much longer durations. Neurotoxicity can occur but generally at higher doses. Other associated toxicities (as per the label) include but are not limited to autoimmune disorders, hepatic impairment, neurotoxicity, and renal impairment.	Considering the low dose and short duration of treatment, these toxicities are less likely to occur. Toxicities will be managed as per standard institutional policy and by trained personnel.

AUTO2 Infusion

Risks associated with the infusion of AUTO2 are presented in Table 8.

Table 8: AUTO2 Infusion – Risks and Mitigation Strategy

Risks	Mitigation Strategy
**Infusion reactions** may occur with the infusion of AUTO2.	The product is autologous and the risk is likely to be low. Patients will be pre-medicated with chlorpheniramine and paracetamol.
**Cytokine-release syndrome** is a key toxicity expected for any CAR T cell treatment. Potentially life-threatening complications of CRS may include cardiac dysfunction, acute respiratory distress syndrome, neurologic toxicity, renal and/or hepatic failure, and disseminated intravascular coagulation. A number of Investigators have noted an association between CRS/macrophage activation syndrome and clinical response when administering CD19 CAR-redirected T cell therapies. The absolute level of risk is difficult to determine.	The risk will be mitigated by adherence to CRS management guidelines as per Lee et al (2014), (Section 8.4) formulated based on prior CAR T cell-gene therapy experience at various institutions. Splitting of CAR T dose at the highest dose level is likely to reduce risk of severe CRS. Additionally, presence of RQR8, which allows selective depletion of APRIL CAR T cells will help manage unresponsive CRS.
**Neurotoxicity** has been seen with the CD19 CARs in patients with leukaemia and lymphoma. Clinical presentation can be variable and manifest as mild to severe encephalopathy, focal neurologic deficits, generalised seizures, severe irreversible neurologic deficits and death in a patient with ALL. The cause of neurotoxicity is not well-understood, although it is reported to be fully reversible in most cases.	The patient will be closely monitored for neurological signs and symptoms, neuroimaging will be performed as appropriate. Patients will be managed with early supportive care as appropriate.
**Thrombocytopenia** is often seen in CAR-treated patients. Thrombocytopenia was observed in 2 patients in a BCMA-CAR study that responded to treatment; in 1 case thrombocytopenia was severe, requiring platelet transfusions for 9 weeks after infusion of the CAR T cells. Thrombocytopenia is believed to be efficacy-related, due to release of cytokines in the bone marrow.	Thrombocytopenia will be managed as per standard institutional guidelines which may include platelet transfusion as necessary.
**Off-tumour toxicity** could be due to either on-target (due to expression of the antigen on non-tumour cells) or off-target (recognition of a molecular target other than BCMA or TACI) interactions. Historically, there have been reports of on-target/off-tumour toxicity with CAR therapy as well as T cell receptor engineered T cells, the details are described in the IB.	Preclinical toxicology indicates the risk to be low as BCMA and TACI expression is largely limited to plasma. Ongoing BCMA CAR studies have not shown any off-target toxicity. Bone marrow monocytes from some MM patients are also known to express TACI, but it remains to be investigated if the expression is adequate to trigger a CAR T cell response. Presence of RQR8, which allows selective depletion of APRIL CAR T cells, will help manage any severe off-target toxicity.
**Cardiac toxicity**: One cardiac-related fatality has been reported with CD19 CAR T cells, although the causality was unclear.	The protocol excludes patients with underlying/prior cardiac history. Additionally, patients will be required to undergo echocardiogram (ECHO) or multiple gated acquisition (MUGA) cardiac scans to demonstrate normal cardiac functioning prior to study enrolment.
**Tumour lysis syndrome** may occur on treatment with AUTO2 due to rapid killing of malignant plasma cells and B cells.	Tumour lysis syndrome is a common toxicity seen with the treatment of haematological malignancies; supportive care will be initiated rapidly.

Risks	Mitigation Strategy
**Hypogammaglobulinemia** may occur as a consequence of depletion of the plasma cells by AUTO2 increasing the risk of infections.	Hypogammaglobulinemia is seen with other commonly used agents such as rituximab. Oncologists are generally well versed with managing patients with chemotherapy associated hypogammaglobulinemia.
**Sepsis:** Sepsis leading to death has been noted in 3 patients treated with very high doses (5×10^8 CAR T cells) of CD19 CAR in a study done at the Hospital of University of Pennsylvania	Gradual titration of dose, splitting of dose at the highest dose level, prophylactic antibiotics and antiviral medications, close monitoring of patients and early intervention is likely to reduce the risk.
**Insertional mutagenesis**: Disruption of the cellular transcriptome by retroviral-mediated insertional mutagenesis can, in rare cases, give rise to haematopoietic stem cells with elevated expression of growth-related genes, which can subsequently result in T cell leukaemia and/or lymphoma.	T cells that have been transduced with a retrovirus have, to date, been proven safe. Unlike haematopoietic stem cells, T cells are highly resistant to retroviral vector-induced transformation. The patients will be monitored for secondary malignancy and survival in a long-term follow-up protocol for a total of 15 years.
**Risks associated with a RCR:** There is a risk that a recombination event may occur during vector production that results in a RCR, which may be pathogenic in humans.	All vector lots are tested for a RCR prior to release to sites. The risks of a RCR are unknown. To date, no patient has developed an RCR with a retroviral based CAR T cell therapy. Patients will be monitored for a RCR by PCR during their scheduled follow-up visits. If a positive signal is confirmed, additional testing will be performed and medical and research experts will be consulted for the optimal treatment approach should any complication arise.
**Dimethyl sulfoxide** which is part of cryopreservative buffer may cause at high doses nausea, vomiting, abdominal cramps, and headache. Some patient may also experience a strong taste of garlic in their mouth or have a similar body odour lasting for a few days. Rarely, they may experience mild or severe cardiac, pulmonary, renal or neurological symptoms.	Most patient are likely to be exposed to a small dose of DMSO, even at the highest dose the median amount of DMSO exposure is likely to be 17.4 grams and maximum 34 grams which is significantly lower than the general institutional max limit of 70 grams. Additionally, at these dose levels the side effects are likely to be mild and short lasting.

APRIL=a proliferation inducing ligand; BCMA=B cell maturation antigen; CAR=chimeric antigen receptor; CD=cluster of differentiation; CRS=cytokine release syndrome; DMSO=dimethyl sulfoxide; ECHO=echocardiogram; IB=Investigator's Brochure; MM=multiple myeloma; MUGA=multiple gated acquisition; PCR=polymerase chain reaction; RCR=replication competent retrovirus; RQR8= safety switch gene; TACI=transmembrane activator and calcium modulator and cyclophilin ligand interactor.

Unknown long-term risks of gene therapy: Following completion of this study, all patients will be enrolled and monitored for SAEs, AEs of interest and survival in a long-term follow-up study.

4 PATIENT POPULATION

Patients will be eligible for the trial if all the inclusion criteria are met and none of the exclusion criteria applies. There will be no exception to the eligibility requirements at the time of registration. Ensuring patient eligibility is the responsibility of the Principal Investigator or other delegated Investigator(s).

4.1 NUMBER OF PATIENTS

It is anticipated that approximately 80 patients will be enrolled (consented) into the study with up to 54 patients receiving the treatment as outlined below:

- Phase I: A dose escalation involving up to a total of 24 patients (up to 6 patients per dose cohort).
- Phase II: Dose expansion involving up to a total of 30 patients.

Phase II of the study will follow Simon's 2-stage optimal design as described in Section 11.1.

4.2 INCLUSION CRITERIA

Patients must meet all the following criteria for study entry:

1. Male or female patients, aged ≥18.
2. Willing and able to give written, informed consent for the current study protocol AUTO2-MM1.
3. Confirmed diagnosis of MM as per IMWG.
4. Measurable disease as defined by any 1 of the following:
    - Serum M-protein ≥500 mg/dL;
    - Urine M-protein ≥200 mg/24 hours;
    - Involved serum free light chain level ≥10 mg/dL, provided serum free light chain ratio is abnormal.
5. Relapsed or refractory disease after either 1 of the following:
    a. Had at least 3 different prior lines[1] of therapy including proteasome inhibitor (e.g. bortezomib or carfilzomib), and IMiD; e.g. thalidomide, lenalidomide or pomalidomide) and alkylator or monoclonal antibody.

    or

    b. Have "double refractory" disease to a proteasome inhibitor and IMiD, defined as progression on or within 60 days of receiving these agents.
6. For females of childbearing potential (defined as <2 years after last menstruation or not surgically sterile), a negative serum or urine pregnancy test must be documented at screening, prior to pre-conditioning and confirmed before receiving the first dose of study treatment.

[1] Induction therapy with stem-cell transplant with or without maintenance is considered a prior therapy.

a. For females who are not postmenopausal (<24 months of amenorrhea) or who are not surgically sterile (absence of ovaries and/or uterus), two methods of contraception comprising of one highly effective method of contraception together with a barrier method must be used during the treatment period and for at least 12 months after the last dose of study treatment. They must agree not to donate eggs (ova, oocytes) for the purposes of assisted reproduction during the study and for 12 months after receiving the last dose of study drug (please refer to Appendix 3).

b. For males, it must be agreed that two acceptable methods of contraception are used (one by the patient – usually a barrier method, and one highly effective method by the patient's partner as defined in Appendix 3) during the treatment period and for at least 12 months after the last dose of study treatment and that sperm will not be donated during the treatment period and for at least 12 months after the last dose of study treatment.

7. Eastern Cooperative Oncology Group (ECOG) Performance Status 0 to 1.
8. Patients must weigh >30 kg.
9. Serum calcium (corrected for serum albumin) or ionised calcium^{+} ≤upper limit of normal (ULN). Note: Prior treatment of hypercalcaemia is permitted and patients may enrol if hypercalcaemia returns to normal with standard treatment.
10. Peripheral blood total lymphocyte count >0.5 × 10^9/L at enrolment and prior to leukapheresis.
11. Patient has adequate bone marrow function without requiring ongoing blood product or granulocyte-colony stimulating factor support, and meets the following criteria:
    - Neutrophils ≥1.0 × 10^9/L;
    - Haemoglobin ≥80 g/L;
    - Platelets ≥75 × 10^9/L.

4.3 EXCLUSION CRITERIA

Patients meeting any of the following exclusion criteria must not be enrolled into the study:

1. Women who are pregnant or lactating.
2. Previous diagnosis of systemic light chain amyloidosis.
3. Prior treatment with investigational or approved gene therapy or cell therapy products.
4. Patient has previously received an allogenic stem cell transplant.
5. Clinically significant, uncontrolled heart disease (New York Heart Association Class III or IV heart failure, uncontrolled angina, severe uncontrolled ventricular arrhythmias, sick-sinus syndrome, or electrocardiographic evidence of acute ischaemia or Grade 3 conduction system abnormalities unless the patient has a pacemaker) or a recent (within 6 months) cardiac event.
6. Uncontrolled cardiac arrhythmia (patients with rate-controlled atrial fibrillation are not excluded).
7. Corrected QT interval (QTc)>470 ms on the screening electrocardiogram (ECG).
8. Patients with evidence of uncontrolled hypertension.

9. Patients with a history of hypertension crisis or hypertensive encephalopathy.
10. Left Ventricular Ejection fraction <50 (by ECHO or MUGA) unless the institutional lower limit of normal is lower.
11. Patients with a history or evidence of deep vein thrombosis or pulmonary embolism requiring ongoing therapeutic anticoagulation at the time of pre-conditioning.
12. Patients with active gastrointestinal bleeding.
13. Patients with any major surgical intervention in the last 3 months, cement augmentation for vertebral collapse is permitted.
14. Significant liver disease: alanine aminotransferase (ALT) or aspartate aminotransferase (AST) ≥3 × ULN, or total bilirubin ≥25 µmol/L (1.5 mg/dL), except in patients with Gilbert's syndrome or evidence of end-stage liver disease (e.g. ascites, hepatic encephalopathy).
15. Chronic renal impairment requiring dialysis, or calculated creatinine clearance <30 mL/min.
16. Active infectious bacterial or viral disease (hepatitis B virus, hepatitis C virus, human immunodeficiency virus, human T-lymphotropic virus or syphilis) requiring treatment.
17. Known active central nervous system involvement of MM. History or presence of clinically relevant central nervous system pathology such as epilepsy, paresis, aphasia, stroke within 3 months prior to enrolment, severe brain injuries, dementia, Parkinson's disease, cerebellar disease, organic brain syndrome, uncontrolled mental illness, or psychosis.
18. Patients receiving corticosteroids at a dose of >5 mg prednisolone per day (or equivalent) that cannot be discontinued.
19. Use of rituximab (or rituximab biosimilar) within the last 3 months prior to AUTO2 infusion.
20. Active autoimmune disease requiring immunosuppression.
21. Past or current history of other neoplasms, except for:
    - Curatively treated non-melanoma skin cancer;
    - Adequately treated in situ carcinoma of the cervix.

    Other cancer curatively treated and with no evidence of disease for at least 2 years or malignancy that in the opinion of the Investigator, with concurrence with the Sponsor Chief Medical Officer or medical designee, is considered cured with minimal risk of recurrence.
22. Received any anti-myeloma therapy within the last 7 days prior to pre-conditioning or leukapheresis. G-CSF should stop 10 days prior to leukapheresis.
23. Received any radiotherapy within the last 7 days prior to pre-conditioning or leukapheresis. Localised radiation to a single site, e.g. for bone pain is permitted at any time.
24. Life expectancy <3 months.
25. Known allergy to albumin, dimethyl sulfoxide (DMSO), cyclophosphamide or fludarabine.

26. Any other condition which in the Investigator's opinion would make the patient unsuitable for the clinical trial.

Prior to initiating the pre-conditioning and for AUTO2 Infusion: Patients meeting any of the following criteria must not initiate pre-conditioning or infusion with AUTO2 or must have treatment delayed until they no longer meet these criteria:

1. Severe intercurrent infection at the time of treatment.
2. Requirement for supplementary oxygen or active pulmonary infiltrates at the time of scheduled AUTO2 infusion.
3. Clinical deterioration of organ functions (hepatic or renal function) exceeding the criteria set at study entry

4.4 EARLY DISCONTINUATION

4.4.1 Discontinuation of Study

A patient should be discontinued if:

- Disease progression as assessed by the Investigator.
- Clinical progression as assessed by the Investigator.
- The patient received concurrent (non-protocol) anticancer treatment.
- Intercurrent illness that prevents administration of pre-conditioning chemotherapy and or AUTO2 administration.
- Intercurrent illness that prevents further follow-up.
- Patient refuses further follow-up.
- Noncompliance with study procedures.

If a patient discontinues the study, this will not result in automatic withdrawal of the patient from the study. Following discontinuation, the patient should complete the End of Study visit as described in Section 9.1.5. The primary reason for treatment discontinuation will be documented in the electronic Case Report Form (eCRF). Patients who discontinue due to intercurrent illness that prevents administration of pre-conditioning chemotherapy and / or AUTO2 administration, may enrol back into the study after discussion with the Sponsor.

Once a patient discontinues, they will be entered into a long-term follow-up study (or continue to be monitored in this study until they enrol onto the long-term study), unless the patient refuses to participate in the study. Patients who discontinue prior to AUTO2 infusion are not eligible for the long-term follow-up study.

Of note, patients becoming pregnant will not be discontinued but will stay on study for follow-up. However, these patients will not undergo any invasive procedure or scans during the follow-up phase nor will they be eligible for retreatment.

Lee L, *et al. J Immunother Cancer* 2023; 11:e006699. doi: 10.1136/jitc-2023-006699

4.4.2 Patient Withdrawal from the Study

A patient will be withdrawn from the study for any of the following reasons:

- Lost to follow-up.
- Withdrawal of consent*.
- The Sponsor discontinues the study.

* Following withdrawal, no further protocol procedures can be performed. However, patients may be followed in the long-term protocol based on consent.

If a patient is lost to follow-up, every reasonable effort must be made by the study site personnel to contact the patient and determine the reason for discontinuation/withdrawal. The measures taken to follow-up must be documented. Data, such as information on survival or potential endpoints, may be collected from healthcare providers, public or medical records, or other sources as allowed by local guidelines and regulations. These data will be collected until the end of the study, even if the patient no longer attends study visits, unless the patient specifically withdraws consent and refuses to allow the collection of further data.

When a patient withdraws before completing the study, the reason for withdrawal is to be documented in the eCRF and in the source document. Patients who withdraw may be replaced at the discretion of the Sponsor.

A patient who withdraws from the study will be invited to participate in the long-term follow-up study. Additionally, research/biomarker samples collected will be retained and used in accordance with the original separate informed consent for research samples.

4.4.3 Withdrawal from the use of samples in future research

The patient may withdraw consent for use of samples for future research. In such a case, samples will be destroyed after they are no longer needed for the clinical study. Details of the sample retention for research are presented in the main patient information sheet and informed consent form.

4.4.4 Procedures for Handling Withdrawals

A patient may, of their own volition, withdraw their consent at any time during the course of the study without any resulting detriment. All data collected up to the point of withdrawal will be maintained in the study database and included in subsequent analyses, as appropriate. Where a patient is withdrawn from the trial at their own request or based on a decision of the Investigator, the follow-up should be maintained for safety review, subject to the continuing consent of the patient. The Investigator will discuss the arrangements for withdrawing from any further study interventions and continuing to be followed for safety purposes.

If a patient is lost to follow-up at a site, every effort should be made to contact the patient’s family doctor/general practitioner to obtain information on the patient’s status.

In the case AUTO2 does not meet the specifications of the release criteria, the case will be discussed with the Principal Investigator. It may be necessary to generate AUTO2 again if time permits (and biological screening repeated if necessary), otherwise the patient will be withdrawn from the study.

4.4.5 Replacement Policy

If a patient is unable to be dosed on the planned day, they may undergo delayed dosing and be re-preconditioned (if appropriate) as long as they continue to meet the study enrolment criteria. Bone marrow sampling and imaging studies may not be repeated if the patient has not received any other anti-myeloma therapy in the interim (excluding steroids and pre-conditioning chemotherapy). Patients undergoing delayed dosing may be evaluable for dose escalation decision making if the SEC so concludes.

Patients that have disease progression prior to completion of the DLT evaluation period or withdrawn from the study for reasons other than toxicity may be replaced, unless the SEC concludes that the patient is evaluable for dose escalation decision making.

5 STUDY DRUG AUTO2

5.1 DRUG DESCRIPTION

AUTO2 is made of enriched T cells retrovirally transduced to express a proliferation inducing ligand (APRIL) CAR and the RQR8 safety switch. The autologous T cells (for IV infusion) transduced to express APRIL CAR, which consists of the receptor-binding site of APRIL expressed extracellularly, hence targeting both BCMA and TACI, coupled to intracellular CD28, OX40 and CD3 zeta signalling domains. In addition, the cells co-express a safety switch gene RQR8 (Philip et al. 2014). RQR8 is a small type 1 transmembrane protein that contains 2 linear fragments of CD20 and the extreme amino-terminal of CD34. There is no post-transduction selection of cells, therefore, AUTO2 also contains non-transduced autologous lymphocytes as a product-related impurity.

AUTO2 is presented in a CryoMACS® bag as a suspension in saline/human albumin solution/DMSO. The final product, AUTO2, may consist of 1 or more CryoMACS® bags for IV infusion.

5.2 AUTO2 MANUFACTURING

The Sponsor is responsible for the manufacturing of the drug product, AUTO2, according to Good Manufacturing Practice (GMP) principles and guidelines applicable to advanced therapy investigational medicinal products. The starting material for generation of AUTO2 is an unstimulated leukapheresate from the patient, which will be performed at the clinical trial centre's local apheresis collection unit. This may require insertion of central venous access and is a day case procedure to collect peripheral blood mononuclear cells (PBMCs) only. Sites will be responsible for ensuring the patient biological screening and leukapheresis procedure, including the labelling and issue of the leukapheresis product are carried out according to the Human Tissue (Quality and Safety for Human Application) Regulations (SI 2007/1523) and in line with local procedures.

The leukapheresis procedure will be performed by the apheresis unit staff as described in Section 7.1. The collected PBMCs will be transported to the Sponsor Manufacturing Unit for generation of AUTO2, where under aseptic GMP conditions, the PBMCs will be transduced with a recombinant viral vector to co-express APRIL and RQR8. Further details regarding this process can be found in the IB.

AUTO2 will be generated by *ex vivo* transduction of activated PBMCs using an engineered murine leukaemia virus-derived retroviral vector (RV22668) containing the APRIL CAR expression cassette and an RQR8 expression cassette. The retroviral vector is produced under GMP conditions by 3-plasmid co-transfection of HEK293T cells and subsequent harvest and purification of the culture supernatant. In brief, cells from the leukapheresate are washed and stimulated with mitogenic ligands and cytokines. After activation, cells are transduced in bags by the retroviral vector. Post-transduction, cells are expanded for between 3 and 7 days to produce adequate dose. The appropriate dose of cells is then transferred to a CryoMACS® bag and formulated with a saline/human albumin solution/DMSO buffer. Full release testing will be performed to pre-defined specifications. AUTO2 manufacture and release will take approximately 4 weeks. AUTO2 will be cryopreserved in sealed CryoMACS® bag maintained in vapour-phase liquid nitrogen environment prior to administration.

Please refer to the AUTO2 IB and Cell Handling Manual for further details on the manufacture of AUTO2, shipment, storage and handling.

5.3 PACKAGING AND LABELLING

AUTO2 is supplied as a cryopreserved cell suspension in a sealed CryoMACS® bag containing the required number of transduced T cells for the planned dose level. The exact pre-cryopreserved cell content is provided on the container label and accompanying records. Each AUTO2 CryoMACS® Freezing Bag will be labelled and will include all the required information as applicable per local regulations.

CryoMACS® Freezing bags are single use only, sterile, containers intended for a single cycle of freezing, storage (down to -196°C), and subsequent thawing (at 37°C) of AUTO2 cells. The CryoMACS® Freezing Bags are comprised of a freezing bag (with access ports) as the primary containment for AUTO2 and an overwrap bag as secondary containment. Additionally, the CryoMACS® Freezing Bag has a built-in label pocket which allows for the insertion of the patient label to include patient identification and product specifications for AUTO2. As part of the bag assembly, 2 spike ports are available which allow access to the bag contents for therapeutic use of the product (via attachment of a sterile transfusion assembly).

All AUTO2 will be released by the Qualified Person residing in the EU.

5.4 SUPPLY AND STORAGE

AUTO2 will be supplied frozen to the study sites (authorised to receive genetically modified organisms, in accordance with local requirements) by the Sponsor. Transport of AUTO2 to the investigator site will be performed by validated shippers under controlled temperature conditions below -150°C. In the case of a temperature excursion occurring during transit, the disposition of AUTO2 will be decided following the review of the temperature data by the responsible Qualified Person or their designee. All temperature excursions outside the storage conditions specified in the Investigators' Brochure/Labels/Trial-Specific Procedures for receipt and storage of AUTO2 must be documented and reported to the Sponsor via the Clinical Research Associate as per the study-specific Cell Handling Manual. The Sponsor must be informed immediately if a patient has been administered AUTO2 where a temperature excursion has occurred.

The Investigator/pharmacist/designated personnel will take an inventory and acknowledge receipt of all shipments of the study product to confirm the shipment condition and content.

AUTO2 must be stored at below -150°C, under controlled temperature and alarmed conditions, in a secure storage area with restricted access and in liquid nitrogen until ready for thawing and preparation as specified in the Cell Handling Manual. The study site will be responsible for the receipt, storage and issue of AUTO2, and will comply with trial-specific procedures and the Cell Handling Manual.

AUTO2 supplied for the AUTO2-MM1 trial is specific for each patient.

5.5 ACCOUNTABILITY AND DESTRUCTION

Study drug accountability and traceability records will be maintained at the site at all times. The identification number of the patient, the administration date, batch number, expiry date and dose of AUTO2 dispensed will be recorded on the appropriate inventory forms.

The Sponsor may authorise unused AUTO2 and any product contact materials to be disposed of as standard clinical waste (typically autoclaving). The site must obtain authorisation from the Sponsor before any AUTO2 is destroyed, and AUTO2 destruction must be documented

on the appropriate form. Further details can be found in the Cell Handling Manual. Documented evidence of destruction will be made available to the Sponsor. Ancillary supplies are not required to be returned to the Sponsor. Accurate records of all AUTO2 received at, dispensed from, returned to, and disposed of by the study site should be recorded on the Product Inventory Log.

6 CONTRAINDICATIONS, CONCOMITANT MEDICATIONS AND PROHIBITED THERAPIES

Any medication the patient is receiving at the time of enrolment and receives for an AE during the study until 60 days post AUTO2 infusion must be recorded along with:

- Reason for use.
- Dates of administration, including start and end dates.
- Dosage information including dose and frequency.

Only concomitant medication related to AEs attributed to AUTO2 treatment will be recorded from Day 60 post AUTO2 infusion onwards. Concomitant medication may be given as medically indicated. Details (including doses, frequency, route and start and stop dates) of the concomitant medication/treatment given must be recorded in the patient's medical records and details entered into the eCRF. Standard drugs required by the patient may be administered alongside the trial protocol.

All patients will receive full supportive care, including pre-medication with antihistamine (dose in accordance with product licence) 30 minutes before T cell infusion, and IV fluids. Transfusions of blood products, antibiotics, analgesics, anti-epileptics and intensive care will also be provided as clinically indicated. Details of these should be recorded in the eCRF. CRS will be managed according to guidelines provided in the protocol (Section 8.4) and as per local policy. All safety management guidelines are only recommendations and deviations from this scheme are allowed according to the Investigator's judgement and local institutional practice.

6.1 ALLOWED CONCOMITANT MEDICATIONS/THERAPIES

Bisphosphonate therapy:

Bisphosphonates can be given to patients with evidence of lytic destruction of bone or with osteopenia, according to the American Society of Clinical Oncology Clinical Practice Guidelines, unless specifically contraindicated (Kyle et al. 2007). If bisphosphonate therapy was not started before the study, then it can be started 4 weeks after AUTO2 infusion and administered as per the standard practice.

Palliative radiotherapy:

Radiotherapy may be given concomitantly for the palliative control of pain. Irradiated lesions will not be evaluable for response.

Other permitted therapies:

The following medications and supportive therapies are examples of support therapies that may be used during the study:

- Antimicrobials or other supportive therapy as required to prevent infections.
- Colony stimulating factors, erythropoietin, and transfusion of platelets and red cells.

Pre and post AUTO2 infusion supportive therapies:

Please refer to Section 8.1.

6.2 PROHIBITED AND CAUTIONARY THERAPIES

Herbal, homeopathic agents or high dose vitamins and mineral supplements:

No herbal, homeopathic agents or high dose vitamin and mineral supplements will be allowed between Day -10 and Day 28 following AUTO2 infusion, unless recommended by the Principal Investigator.

Corticosteroids and immunosuppressant (except for managing treatment related toxicity):

Patients should not be receiving corticosteroids at doses of >5 mg prednisolone or equivalent at the time of AUTO2 infusion. The use of immunosuppressants such as high dose corticosteroids should be avoided where possible, as these are likely to influence the efficacy and possibly safety of AUTO2. Corticosteroids should also be avoided post-infusion if possible, although it is recognised that their use may be required in the context of development of CRS or infusion reactions. Physicians may use any medication as clinically appropriate and necessary to manage emerging AEs. The use of other immunosuppressants should be discussed with the Sponsor's Medical Monitor.

Anti-cancer therapies:

In general, patients should not receive other anti-cancer therapy (including radiotherapy except for pain management as mentioned above) or any other investigational drugs after AUTO2 infusion. Administration of other systemic anti-cancer therapy at any time after AUTO2 infusion will be considered an indicator of treatment failure (progressive disease). However, palliative radiotherapy for symptom control can be administered without necessarily indicating progressive disease. Patients who have been administered AUTO2 and subsequently require alternative anti-cancer therapy will complete the End of Study visit and roll on to a follow-up protocol.

Intervening/bridging anti-cancer therapy administered during the screening period is permitted as long as compliant with the defined washout period. This therapy will be recorded in a new medical history eCRF covering the whole screening period. Please refer to see Section 10.3.1 and eCRF completion guidelines.

Investigators can use any medication based on their clinical judgement and local institutional practice to optimise patient's safety.

6.3 OVER-DOSAGE

There is currently no experience of overdose of AUTO2 as no clinical studies have been performed to date. There is no specific treatment for an overdose of AUTO2. In event of overdose, any adverse reactions could be treated symptomatically. In the event of unmanageable toxicity, rituximab may be used to deplete AUTO2.

AUTO2 cells will be provided in patient specific dose aliquots and will be administered by trained staff in a hospital setting, therefore the chance of overdose is unlikely.

6.4 DIETARY AND LIFESTYLE RESTRICTIONS

No dietary restrictions are recommended. A normal balanced diet is recommended; the patient may also continue his/her normal diet as appropriate.

Donating blood products and organs:

Patients should not donate blood, organs, tissue and cells to others for transplant purposes after receiving AUTO2.

Lee L, *et al. J Immunother Cancer* 2023; 11:e006699. doi: 10.1136/jitc-2023-006699

7 STUDY PROCEDURES

7.1 LEUKAPHERESIS

Enrolled patients who meet the eligibility criteria will be scheduled for leukapheresis to have their white blood cells collected. The leukapheresis may be repeated if inadequate number of cells has been collected, the leukapheresate is contaminated, or additional leukapheresate is necessary for re-treatment. The leukapheresate will be shipped to the Sponsor to prepare the cell product (Cell Handling Manual). Leukapheresis may be performed as per the preferred institutional process.

The leukapheresate is considered to be the starting material for the manufacture of the ATIMP, AUTO2. The total cell number that is required for successful manufacture varies according to the dose level. The target collection may vary from 1×10^8 to 1×10^9 (for the highest dose proposed) leukocytes. If the collection is below this number, the Sponsor will advise as to the feasibility of successful manufacture. If collection is determined to be inadequate, then collected cells may be used for research purposes as per the consent. The leukapheresate will be transported to the Sponsor for generation of AUTO2 at a temperature of 2 to 8°C within 24 hours, ideally within 3 hours (Cell Handling Manual). Further details regarding this process can be found in the Cell Handling Manual.

Each leukapheresate will be identified by a unique patient ID number plus any additional patient identifiers as allowed as per local regulations.

7.2 PRE-CONDITIONING CHEMOTHERAPY FOR AUTO2 THERAPY

Cyclophosphamide and fludarabine will be used for the pre-conditioning regimen prior to administration of AUTO2. The patients should receive fludarabine and cyclophosphamide daily for 3 days. The dosing is described below and fludarabine will be given first.

- Fludarabine 30 mg/m^2 followed by cyclophosphamide 300 mg/m^2 Day 1 (Day -6)
- Fludarabine 30 mg/m^2 followed by cyclophosphamide 300 mg/m^2 Day 2 (Day -5)
- Fludarabine 30 mg/m^2 followed by cyclophosphamide 300 mg/m^2 Day 3 (Day -4)

The pre-conditioning chemotherapy should be completed 4 days (+/- 1 day) prior to AUTO2 infusion.

Fludarabine will be given by IV infusion over approximately 30 minutes in sodium chloride 0.9%. For patients with renal impairment (glomerular filtration rate [GFR] 30 to 60 mL/min/1.73m^2 [corrected]), the dose of fludarabine should be reduced according to routine clinical practice (generally by 25%). When patients receive both cyclophosphamide and fludarabine, the **fludarabine will be given first**.

Cyclophosphamide will be given by IV infusion over approximately 30 minutes. Adequate pre and post hydration for up to 4 to 6 hours (or as per institutional practice) should be given post infusion to induce diuresis. Use of mesna for the prescribed dose is generally considered unnecessary but may be considered based on institutional practice. Cyclophosphamide dose may be reduced if the leukocyte count is <2500 cells/ microliter with 6 hours post hydration.

In the event of severe infusion reaction, the cyclophosphamide or fludarabine infusion should be stopped and the patient treated as clinically indicated. When the patient has recovered, the infusion may be restarted. If the patient develops an allergic reaction or has a history of

allergy to the drugs in the pre-conditioning regimen, then alternative drugs as per the institutional practice may be considered after discussion with the Sponsor.

For additional information regarding both the drugs please follow the Summary of Product Characteristics.

7.2.1 Supply of Cyclophosphamide and Fludarabine

Cyclophosphamide and fludarabine are considered to be non-investigational medicinal products as they are not being tested or used as a comparator used in this trial. Cyclophosphamide and fludarabine will be used to induce a physiological response in the current study as a pre-conditioning treatment, prior to ATIMP treatment. Both drugs are authorised and commercially available in the Member States. The Investigators will be responsible for their own supply of cyclophosphamide and fludarabine. Sufficient quantities of cyclophosphamide and fludarabine will be dispensed to cover the prescribed dose and will be prepared as per site Standard Operating Procedures and accordingly to manufacturer recommendations. Cyclophosphamide and fludarabine are cytotoxics and must be handled with care in accordance with local policy. Good aseptic practice must be employed when preparing cyclophosphamide and fludarabine solutions for infusion.

7.2.2 Accountability of Cyclophosphamide and Fludarabine

Pharmacy records should be kept of the cyclophosphamide and fludarabine dispensed to trial patients.

7.3 AUTO2 TREATMENT AND PATIENT MONITORING

7.3.1 Treatment Allocation and Blinding

7.3.1.1 Patient Assignment

After providing written informed consent, the patient will be issued with a unique patient identification number. The patient identification number will be used to identify the patient for the duration of the study. Patient identification numbers will not be reassigned or reused.

7.3.1.2 Randomisation and Blinding Procedures

Randomisation will not be used in this study. Patients will be allocated to receive study treatment in the order in which they qualify for the study. As this is an open-label study, blinding procedures are not applicable.

7.3.2 AUTO2 Administration

AUTO2 (fulfilling the release criteria) will be administered as a single rapid IV infusion under 30 minutes from AUTO2 being thawed on Day 0 in an in-patient setting. Full details will be provided in the Cell Handling Manual. Only the Investigator or Investigator’s designee will dispense the study product. The Investigator or Investigator’s designee will verify the dose calculation and dose preparation in each treatment period. Details of the exact dose, time of preparation, and time of study medication administration will be documented in the applicable study records.

In brief, AUTO2 will be thawed in a 37°C water bath under sterile conditions and the entire contents of the bag will be given as an IV infusion through a central or peripheral venous line

by a study research nurse experienced in the administration of cellular blood products as per the trial-specific procedure. The infusion line and the bag should be flushed as described in the Handling Manual to ensure all cells have been administered.

7.3.3 Monitoring During and After Drug Administration

All patients will receive full supportive care, including pre-medication (see Section 8.2) 30 minutes before T cell infusion, and IV fluids (approximately 125 mL/m^2/hour) from 1 hour prior to the T cell infusion until 2 to 4 hours post infusion. The patient will be monitored closely every 15 min (± 5 min) for the first hour and then every 30 minutes (± 10 min) for the next 3 hours as clinically indicated and thereafter monitored as per hospital policy but no less than 3 times a day. Transfusions of blood products, antibiotics, analgesics, and intensive care will also be provided as clinically indicated. Prophylaxis for tumour lysis syndrome (TLS) may be initiated if the risk is considered high or for all new patients if TLS occurs at a certain dose level. In the event of allergic adverse reactions, anti-histamines and hydrocortisone may be administered, as well as oxygen and salbutamol in the event of respiratory distress. Patients will be admitted to hospital for at least 10 days (or longer) for monitoring after receiving AUTO2 or until all AUTO2 related non-haematological toxicities have returned to ≤ Grade 1 or baseline, or longer as clinically necessary.

After completing the study (Month 24, post AUTO2 administration or after disease progression), the patients will be followed on a separate long-term follow-up protocol (AUTO-LT1).

7.3.4 Dose Delay

In the event that the pre-conditioning regimen is interrupted for intercurrent illness or other reasons, the patient may complete or recommence the preparative regimen after recovery, according to the Investigator's judgment after consultation with the Sponsor. Patients will be closely monitored during and after the pre-conditioning regimen. If the patient has completed the pre-conditioning regimen but is unable to receive AUTO2 on Day 0 for any reason, clinical judgement will be used to decide whether it is appropriate to delay the administration of AUTO2 or to wait for the patient's safety blood results to recover sufficiently before repeating the pre-conditioning regimen and administering AUTO2.

If the patient is deemed unsuitable to receive the AUTO2, they will be discontinued from the clinical trial (see Section 4.4.1 discontinuation of study) and replaced. Each case will be discussed with the Sponsor.

7.3.5 Interruption of AUTO2 Infusion

In the event of severe infusion reaction, the AUTO2 infusion should be stopped and the patient treated as clinically indicated. When the patient has recovered, the infusion may be restarted as long as the cells remaining in the bag are given within the 30 minutes of thawing. In the event of the dose consisting of multiple bags, interruption of AUTO2 should not be greater than 2 hours between the first and the last bag administered. If an infusion is interrupted for mechanical, technical or any other reason, then this should be dealt with according to local practice and the infusion restarted as soon as possible and administered within 30 minutes of thawing. In case of uncertainty, individual cases should be discussed with the Sponsor. Duration of Treatment

Lee L, et al. J Immunother Cancer 2023; 11:e006699. doi: 10.1136/jitc-2023-006699

In most patients, it is expected that AUTO2 will be given once. However, if a patient has sufficient AUTO2 remaining from the original manufacture and meets the re-treatment criteria, a second treatment may be given (see re-treatment of patient Section 7.3.6).

7.3.6 Re-treatment of Patients

It is expected that most patients will receive a single as part of their treatment. However, some patients may have sufficient transduced T cells leftover, or leukapheresate which can be used to manufacture transduced cells, or they may be eligible for repeat leukapheresis, for a re-treatment at a later date. Such patients may receive a re-treatment of their own stored AUTO2. Prior to re-treatment, the patient needs to meet the following criteria:

1. Patient shows evidence of minimal or no AUTO2 survival (<0.2 × 10^9/dL AUTO2 cells) and no significant anti-tumour effect (PR or better) after the 1st dose, and the 1st treatment dose is considered to be sub therapeutic (e.g. lower than the RP2D).

OR

2. There was objective clinical evidence of anti-tumour activity following the previous AUTO2 infusion (i.e. a CR, PR, or stable disease for at least 6 weeks).

OR

3. The patient now has evidence of progressive disease in the context of declining levels of AUTO2. Circulating levels AUTO2 cells must be low (<0.2 × 10^9/L) or undetectable for at least 2 weeks prior to the second infusion.

AND all of the following:

4. The patient tolerated the first infusion without dose-limiting or other severe or unmanageable toxicity for a follow-up period of at least 4 weeks.
5. The patient still fulfils the trial entry criteria for ECOG and renal function required to tolerate another pre-conditioning treatment.
6. The patient has not received any alternative systemic anti-cancer therapy within 7 days of pre-conditioning (palliative radiotherapy and/or corticosteroids for symptom control are allowed).

Patients undergoing a second AUTO2 infusion may receive the same or modified pre-conditioning as clinically appropriate. If bridging therapy is required, it should be with an IMiD-based regimen, when clinically feasible. The dose of AUTO2 can be up to the highest dose level cohort that is actively recruiting patients. Patients who are being re-treated will not be considered when making dose escalation decisions in that dose cohort, unless a DLT occurs as per DLT criteria in Section 3.1.4. Depending on the number of cells available, an intermediate dose may also be administered if considered appropriate. The decision to re-treat a patient will be made by the treating Investigator and Sponsor in consultation with the SEC. Patients re-treated will be monitored in a similar way to patients being treated for the first time, in that they would start evaluation and management as defined in the protocol, starting from the pre-conditioning stage. A patient with progressive disease that receives re-treatment will be censored from primary efficacy analysis at point of progression but will continue to remain on the study.

8 GUIDELINES FOR PREVENTION, MONITORING AND MANAGEMENT OF ADVERSE EVENTS

The guidelines detailed in Table 9 are suggested ways of managing key toxicities, however patients may be managed as per local institutional guidelines and or using clinical judgement.

8.1 GENERAL SUPPORTIVE CARE GUIDELINES FOR PATIENTS RECEIVING CAR T CELL THERAPY

Table 9: General Supportive Care Guidelines for Patients Receiving CAR T Cell Therapy

Toxicity	Preventive and Supportive Care Interventions
Constitutional	Administer acetaminophen for symptomatic management of fevers in patients with normal hepatic function;
	Provide cooling blankets for fevers >40°C;
	Avoid corticosteroids and non-steroidal anti-inflammatory drugs; and
Respiratory	Monitor for oxygen saturation at every visit, a significant decrease in oxygen saturation at room air should be investigated and managed with supportive care including supplemental oxygen, anti-microbials and ventilator support as appropriate.<br>Patients with an oxygen requirement of ≥40% (or lower based on emerging data) in setting of CRS should receive treatment with tocilizumab.
Cardiovascular	Stop or taper antihypertensive medications prior to cell infusion;
	Monitor vital signs at least every 4 to 6 hours during hospital or ambulatory care stay
	Monitor vital signs every 2 hours in patients with fevers and tachycardia;
	Initiate replacement IV fluids for patients with poor oral intake or high insensible losses to maintain net even fluid balance;
	Administer IV fluid boluses for patients with systolic blood pressure less than their pre-infusion baseline;
	Patients with a systolic blood pressure <80% of their pre-infusion baseline and <100 mm Hg receive a 1 litre normal saline bolus;
	Patients with a systolic blood pressure <85 mm Hg receive a 1 litre normal saline bolus regardless of baseline blood pressure;
	Patients receiving >1 IV fluid bolus for hypotension or patients in the intensive care unit for toxicity management have a serum troponin drawn, and an ECG and an ECHO performed to evaluate for cardiac toxicity; and
	Norepinephrine is the preferred first-line vasopressor for patients with hypotension initiated on vasopressor support.
Infectious disease	Initiate prophylactic antimicrobials, such as trimethoprim-sulfamethoxazole, for *Pneumocystis* prophylaxis prior to conditioning chemotherapy;
	Initiate prophylactic antimicrobials, such as acyclovir or valacyclovir, for herpes virus prophylaxis prior to conditioning chemotherapy; and
	All patients with fevers and neutropenia have blood cultures drawn and assessment for broad-spectrum antibiotics as per institutional practice.

Lee L, et al. J Immunother Cancer 2023; 11:e006699. doi: 10.1136/jitc-2023-006699

Toxicity	Preventive and Supportive Care Interventions
Hypogammaglobulinemia	Consider IV Ig administration if the serum IgG level is <400mg/dl and or clinically appropriate.
Hematologic	Initiate allopurinol for TLS prophylaxis in patients without a contraindication prior to conditioning chemotherapy; if indicated.
	Transfuse packed red cells for goal haemoglobin of ≥80 g/dL;
	Transfuse platelets for a goal platelet count of ≥20,000/µL;
	Monitor complete blood count with differential at least daily. When absolute neutrophil count decreases to <500/µL, initiate granulocyte colony stimulating factor support. Continue until absolute neutrophil count increases to ≥1500 µL;
	Transfuse fresh frozen plasma with a goal of normalisation of partial thromboplastin time (PTT) in patients with a PTT >1.5-fold above the ULN; and
	Transfuse cryoprecipitate to maintain fibrinogen of ≥100 mg/dL. If patient is bleeding, a higher level of fibrinogen should be maintained.
Neurologic	The nursing staff conducts focused neurologic examinations at least every 4 to 6 hours in patients experiencing neurologic toxicity;
	Perform brain MRI in any patient experiencing neurologic toxicity;
	Perform lumbar puncture to evaluate for infectious pathogens, cytokine levels, and CAR T cell levels in patients experiencing neurologic toxicity whenever feasible;
	Consider a neurology consultation for any patient experiencing neurologic toxicity; and
	Standard antiepileptic medications for patients with active seizures.

CAR=chimeric antigen receptor; ECG=electrocardiogram; ECHO=echocardiogram; Ig=immunoglobulin; IV=intravenous; MRI=magnetic resonance imaging; PTT=partial thromboplastin time; ULN=upper limit of normal.
Reference: (Brudno and Kochenderfer 2016).

8.2 PRE- AND POST-INFUSION SUPPORTIVE THERAPY

AUTO2 is an autologous product, with fully human CAR construct and is less likely to be immunogenic and induce an infusion or hypersensitivity reaction. However, the following medications should be given 30 minutes before the study drug infusion: paracetamol (acetaminophen; 650 to 1000 mg orally) and an antihistamine (diphenhydramine 25 to 50 mg, or equivalent). These medications may be discontinued based on emerging data. In addition, pre-infusion medications listed in Table 10 may also be administered if necessary.

Post-infusion medication listed in Table 10 may be considered following AUTO2 infusion if necessary. Post-infusion medication(s) may be continued for up to 48 hours after the infusion. Use of additional supportive care measures may be instituted as clinically necessary at the discretion of the Investigator.

Lee L, et al. J Immunother Cancer 2023; 11:e006699. doi: 10.1136/jitc-2023-006699

Table 10: Pre- and Post-infusion Medications

Medication	Dose	Administration	Pre-infusion	Post-infusion
**Antihistamine**	Diphenhydramine/ Chlorpheniramine (10 to 20 mg) or equivalent	Oral – administer at least 1 hour prior to study drug<br><br>IV or as appropriate – administer at least 30 minutes prior to study drug	X	
	Diphenhydramine/ Chlorpheniramine (8 to 16 mg) or equivalent	Oral - as clinically indicated		Optional
**Antipyretic**	Paracetamol (acetaminophen) (650 to 1000 mg) or equivalent	Oral - administer at least 30 minutes prior to study drug	X	
	Paracetamol (acetaminophen) (650 to 1000 mg) or equivalent	Oral - as clinically indicated		Optional
**H_2-antagonist**	Ranitidine or equivalent (50 mg)	IV- start infusion 30 minutes prior to study drug	Optional	
**Antiemetic**	Ondansetron (16 mg) or equivalent	IV - start infusion 30 minutes prior to study drug	Optional	
	Ondansetron (8 mg) or equivalent (long or short acting agents)	Oral - as clinically indicated		Optional

IV=intravenous.
Note: Steroids may be used in case of severe reactions not controlled by other measures.

8.3 TREATMENT OF INFUSION RELATED REACTIONS

Patients who experience infusion-related reactions (to pre-conditioning or AUTO2) that manifest as wheezing, flushing, hypoxemia, fever, chills, rigors, bronchospasm, headache, rash, pruritus, arthralgia, hypo- or hypertension or other symptoms, should have the symptoms managed according to the recommendations provided in Table 11 or as per institutional practice. All NCI CTCAE Grade 3 or 4 infusion-related reactions should be reported within 24 hours to the Sponsor. If the event meets the criteria of an SAE, follow SAE reporting criteria in Section 10.1.3.

Lee L, et al. J Immunother Cancer 2023; 11:e006699. doi: 10.1136/jitc-2023-006699

Table 11: Guidelines for the Management of Infusion-related Reactions

NCI CTCAE Grade	Treatment/Intervention	Premedication at Subsequent Dosing
**Grade 1**<br>Mild reaction; infusion interruption not indicated; intervention not indicated	**No intervention indicated**;<br>Monitor patient as medically indicated until recovery from symptoms.	May consider chlorpheniramine 10 to 20 mg or equivalent and or paracetamol (acetaminophen) 650 to 1000 mg if not instituted (see Table 10).
**Grade 2**<br>Moderate reaction; requires therapy or infusion interruption but responds promptly to symptomatic treatment	**Interrupt infusion**<br>Start IV fluids; give diphenhydramine 50 mg (or equivalent) IV and/or paracetamol 500 to 1000 mg (acetaminophen); consider bronchodilator therapy; may also consider corticosteroids if necessary; monitor patient closely until recovery from symptoms.<br>Restart infusion if AUTO2 dose has not been fully administered<br>**Symptoms recur:** Stop and discontinue further infusion.<br>The amount of AUTO2 infused must be recorded on the eCRF.	Diphenhydramine/ Chlorpheniramine 10 to 20 mg or equivalent and/or paracetamol (acetaminophen) 500 to 1000 mg if not instituted (see Table 10).
**Grade 3 or 4**<br>Severe reaction;<br>**Grade 3**: prolonged (i.e. not rapidly responsive to symptomatic medication and/or brief interruption of infusion); recurrence of symptoms following initial improvement; hospitalisation indicated for other clinical sequelae (e.g. renal impairment, pulmonary infiltrates)<br>**Grade 4:** life-threatening; pressor or ventilator support indicated	**Stop Infusion**<br>Start IV saline infusion; recommend bronchodilators, epinephrine 0.2 to 1 mg of a 1:1000 solution for subcutaneous administration or 0.1 to 0.25 mg of a 1:10000 solution injected slowly for IV administration, and/or diphenhydramine/chlorpheniramine 50 mg IV with methylprednisolone 100 mg IV (or equivalent), as needed, and other drugs as appropriate.<br>Patients should be monitored until the Investigator is comfortable that the symptoms will not recur. Investigators should follow their institutional guidelines for the treatment of anaphylaxis. In the case of late-occurring hypersensitivity symptoms (e.g. appearance of a localised or generalised pruritus within 1 week after treatment), symptomatic treatment may be given (e.g. oral antihistamine or corticosteroids), as appropriate.	**Grade 3:** Discuss with Sponsor medical monitor about merits of completing AUTO2 infusion if full dose has not been administered.<br>**Grade 4:** Permanently discontinue any remaining AUTO2.
**General**	Appropriate personnel and appropriate resuscitation equipment should be available in or near the infusion room and a physician should be readily available during the infusion of study drug.	

CTCAE=Common Terminology Criteria for Adverse Events; eCRF=electronic case report form; IV=intravenous; NCI=National Cancer Institute.

8.4 GRADING AND MANAGEMENT OF CYTOKINE RELEASE SYNDROME

Cytokine release syndrome is a known toxicity with CAR T therapies. Clinical symptoms indicative of CRS may include but are not limited to fever (with or without rigors), arthralgia, nausea, vomiting, tachycardia, hypotension, headache, confusion, tremor, and delirium. Potentially life-threatening complications of CRS may include cardiac dysfunction, acute respiratory distress syndrome, neurologic toxicity, renal and/or hepatic failure, and disseminated intravascular coagulation. Patients should be closely monitored for early signs and symptoms indicative of CRS. Patients with temperature ≥38.9°C within the first 36 hours after AUTO2 infusion are at higher risk of severe CRS and should be monitored closely. Trained clinical personnel should be prepared to intervene in the event of CRS. Resources necessary for resuscitation and supportive care including intensive care should be readily available. Recommendations for the clinical management of CRS are provided in Table 12.

Table 12: Severity Grading and Management of CRS

CRS Grade	Treatment
**Grade 1**<br>Symptoms are not life threatening and require symptomatic treatment only, e.g. fever, nausea, fatigue, headache, myalgia, malaise.	Supportive care according to institutional standards including analgesics and antipyretics, assess and treat for neutropenic infections<br>Consider tocilizumab or siltuximab for persistent (lasting >3 days) and refractory fever
**Grade 2**<br>Symptoms require and respond to moderate intervention<br>Oxygen requirement <40% or<br>Hypotension responsive to fluids or low dose of 1 vasopressor or NCI CTCAE Grade 2 organ toxicity or Grade 3 transaminitis.	Supportive care including fluid substitution is recommended.<br>Use tocilizumab (or siltuximab) early if persistent fever of ≥39°C despite antipyretics for 10 hours, persistent/ recurrent hypotension after initial fluid bolus, and initiation of oxygen supplementation.<br>Low-dose vasopressor therapy.<br>Low-flow-oxygen (<40% fraction of inspired oxygen).<br>Use dexamethasone if hypotension persists after 1 to 2 doses of anti-IL-6 therapy, dexamethasone can be used at 10 mg IV every 6 hours.<br>Hospitalisation for observation (for longer than 24 hours) as clinically appropriate.
**Grade 3**<br>Symptoms require and respond to aggressive intervention<br>Oxygen requirement ≥40% or<br>Hypotension not responsive to fluid therapy within 24 hours and requiring high dose or multiple vasopressors or NCI CTCAE Grade 3 organ toxicity or Grade 4 transaminitis.	Intensive care should be considered.<br>Immunosuppressive therapy with tocilizumab (or siltuximab), repeat dose of tocilizumab at 6 to 12 hours if no clinical improvement.<br>Consider adding siltuximab if not previously administered.<br>Add steroids if unresponsive within 24 hours, dexamethasone 10 mg IV every 6 hours; if refractory, increase to 20 mg IV every 6 hours.<br>Consider anti-tumour necrosis factor (TNF) antibodies as clinically appropriate.<br>Multiple vasopressors or high-dose vasopressors.<br>Oxygen (flow ≥40% fraction of inspired oxygen) (nasal cannula oxygen, high flow oxygen, continuous positive airway pressure or bilevel positive airway pressure).<br>May consider use of rituximab to activate safety-switch if unresponsive to standard immunosuppressive

**CRS Grade**	**Treatment**
	therapies.
**Grade 4**<br>Life-threatening symptoms, requirement for ventilator support or NCI CTCAE Grade 4 organ toxicity (excluding transaminitis).	Intensive care.<br>Immunosuppressive therapy with tocilizumab +/-, siltuximab and high dose steroids (methylprednisolone 1 g/day IV).<br>If unresponsive, consider alternative agents such as anti-TNF, and other agents as appropriate (Gargett et al. 2015, Kenderian et al. 2017, Ruella et al. 2017).<br>Use of rituximab to activate safety-switch.
**Grade 5**<br>Death	

CTCAE=Common Terminology Criteria for Adverse Events; NCI=National Cancer Institute; TNF=tumour necrosis factor.
Reference: (Lee et al. 2014).

Details of the steroid doses and supportive medication doses are presented in Table 13.

Table 13: Definitions and Doses of Vasopressors

Definition of **High-Dose** Vasopressors	
**Vasopressor**	**Dose for ≥3 hours**
Noradrenaline monotherapy	≥0.2 µg/kg/min
Dopamine monotherapy	≥10 µg/kg/min
Phenylephrine monotherapy	≥200 µg/min
Adrenaline monotherapy	≥0.1 µg/kg/min
If on vasopressin	High dose if vasopressin + noradrenaline equivalent of ≥0.1 µg/kg/min (using Vasopressin and Septic Shock Trial formula)
If on combination vasopressors (not vasopressin)	Noradrenaline equivalent of ≥20 µg/min (using Vasopressin and Septic Shock Trial formula)

An overview of the CRS management is presented in Figure 5.

Figure 5: CRS Management Overview

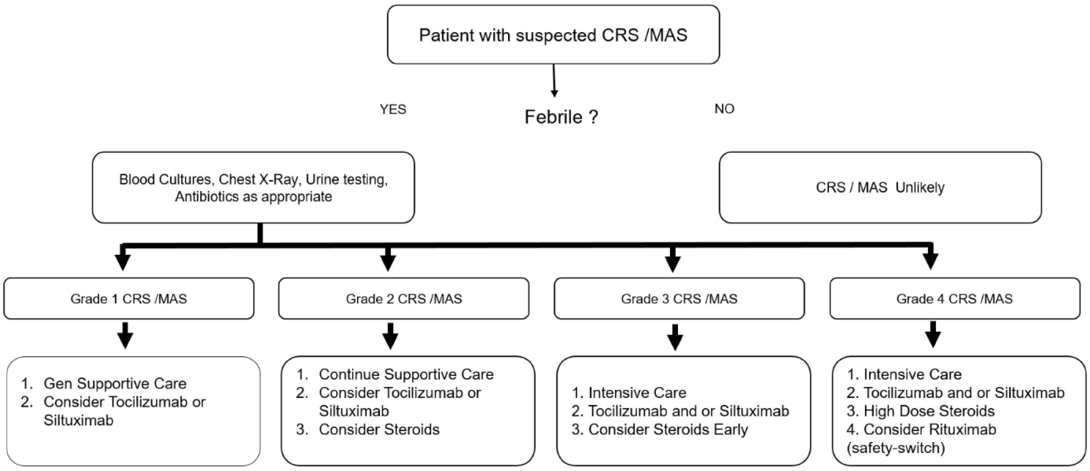

The pharmacologic management of CRS is presented in Table 14.

Table 14: Pharmacologic Management of CRS

Drug	Indication	Dose
Tocilizumab<br><br>And/Or<br><br>Siltuximab	**Consider using (early use criteria) if**<br>Persistent fever of ≥39°C despite antipyretics for 10 hours;<br>Persistent/ recurrent hypotension after initial fluid bolus, and<br>Initiation of oxygen supplementation (Gardner et al. 2016).<br>**Highly recommended - if**<br>Left ventricular ejection fraction <40% by ECHO;<br>Creatinine >2.5-fold higher than the most recent level prior to CAR T cell infusion;<br>Norepinephrine requirement at a dose >2 pg/min for 48 hours since the first administration of norepinephrine, even if administration is not continuous;<br>Systolic blood pressure of 90 mm Hg that cannot be maintained with norepinephrine;<br>Oxygen requirement of fraction of inspired oxygen >50% or more for more than 2 hours continuously;<br>Dyspnoea that is severe enough to potentially require mechanical ventilation;<br>Activated PTT >2 × the ULN;<br>Clinically-significant bleeding; and<br>Creatine kinase >5 × the ULN for longer than 2 days.	Tocilizumab: 8 mg/kg infused over 1 hour, dose not to exceed 800 mg.<br>Repeat dose if no response within 6 to 12 hours and consider steroids as below.<br><br>Siltuximab: 11 mg/kg infused over 1 hour.
Methylprednisolone	Cytokine Release Syndrome toxicity refractory to tocilizumab.	1 to 2 mg/kg IV every 12 hours<br>For Grade 4 CRS use 1 gm/day.

CAR=chimeric antigen receptor; ECHO=echocardiogram; IV=intravenous; PTT=partial thromboplastin time; ULN=upper limit of normal.
Reference: (Brudno and Kochenderfer 2016).

8.5 MANAGEMENT OF TUMOUR LYSIS SYNDROME

Treatment with CAR T cells, such as AUTO2, can lead to rapid killing of malignant B cells, which can be associated with a release of intracellular ions and metabolic by-products into the systemic circulation and can thus, result in signs and symptoms indicative of TLS. Clinically, TLS can be characterised by rapid development of hyperuricemia, hyperkalaemia, hyperphosphatemia, hypocalcaemia, and potentially acute renal failure.

Early recognition and monitoring of signs and symptoms of patients at risk for TLS, including identification of abnormal clinical and laboratory values, can lead to successful prevention of the serious clinical complications of the condition. Supportive care should be initiated according to the institutional standards and at the Investigator’s discretion.

Management of TLS, including dehydration and management of abnormal laboratory test results such as hyperkalaemia, hyperuricemia, and hypocalcaemia, is highly recommended. It is also recommended that high-risk patients, i.e. those with a high tumour burden, be treated prophylactically in accordance with local standards (e.g. rehydration; diuretics; allopurinol and medication to increase urate excretion).

8.6 GRADING AND MANAGEMENT OF NEUROTOXICITY

Neurotoxicity has been seen with the CD19 CARs in patients with leukaemia and lymphoma, with a time course similar to CRS. The cause of neurotoxicity is not well-understood, although it is reported to be fully reversible. Transient neurological complications have also been reported with CD19 Bispecific T Cell Engagers, suggesting that the target may have some relevance. In neither case do these toxicities correlate with CRS/macrophage activation syndrome. Ongoing studies with BCMA CAR in myeloma patients have not reported any severe neurotoxicity or only reported occasional cases of neurotoxicity. However, close attention should be paid to neurological signs and symptoms.

Neurological AEs can manifest as central abnormalities (e.g. aseptic meningitis or encephalitis) or peripheral sensory/motor neuropathies (e.g. Guillain-Barre Syndrome). The onset can be early after treatment, within hours to days. In general severe neurotoxicity is observed at or after the peak CRS. Patients with baseline thrombocytopenia may be at higher risk of severe neurotoxicity. Early recognition and treatment of neurologic AEs is critical to their management. Patients should be closely monitored for neurologic signs and symptoms such as headaches, confusion, alterations in wakefulness, hallucinations, dysphasia, ataxia, apraxia, facial nerve palsy, tremor, dysmetria, and seizures. These events are likely to be reversible; they should be managed with supportive care as appropriate.

Neurotoxicity may also be caused by fludarabine but generally at higher doses (Helton et al. 2013) than those being administered in the AUTO2-MM1 protocol. Symptoms including objective weakness, agitation, confusion, seizures, visual disturbances, optic neuritis, optic neuropathy, blindness and coma have been reported in chronic lymphocytic leukaemia (CLL) patients treated with multiple cycles of fludarabine. As described in Section 3.2.3 death of 3 young adults ALL patients due to cerebral oedema was partly attributed to fludarabine; however, two additional deaths due to cerebral oedema in the absence of fludarabine indicates that was not the cause. Moreover, it now appears that cerebral oedema is likely a consequence of very rapid CAR T cell proliferation, driven by the CD28 co-stimulatory domain, this event has not been reported with CARs containing a 41BB-ζ costimulatory domain which results in a slower proliferation. Patients should be closely monitored and managed as appropriate. Please see Table for suggested grading and guidelines for management of neurotoxicity (Neelapu et al. 2017); however, the patient may be managed as per institutional management guidelines.

Table 15: Grading of Neurological AEs and CAR-T-Cell-Related Encephalopathy Syndrome (CRES)

Symptoms or Sign	Grade 1	Grade 2	Grade 3	Grade 4
General neurotoxicity by NCI CTCAE	Mild	Moderate	Severe	Life threatening
CAR-T-cell-related encephalopathy syndrome<br>Neurological assessment score (by CARTOX-10*)	7–9 (mild impairment)	3–6 (moderate impairment)	0–2 (severe impairment)	Patient in critical condition, and/or obtunded and cannot perform assessment of tasks
Raised intracranial pressure	NA	NA	Stage 1–2 papilloedema‡, or CSF opening pressure <20 mmHg	Stage 3–5 papilloedema‡, or CSF opening pressure ≥20 mmHg, or cerebral oedema
Seizures or motor weakness	NA	NA	Partial seizure, or non-convulsive seizures on EEG with response to benzodiazepine	Generalized seizures, or convulsive or non-convulsive status epilepticus, or new motor weakness

CAR=chimeric antigen receptor; CARTOX-10=CAR-T-cell-therapy-associated toxicity 10-point neurological assessment CSF=cerebrospinal fluid; EEG=electroencephalogram; NA=not applicable.

*In the CARTOX-10, one point is assigned for each of the following tasks that is performed correctly (normal cognitive function is defined by an overall score of 10):

Orientation to year, month, city, hospital, and President/Prime Minister of country of residence (total of 5 points);

Name three objects — for example, point to clock, pen, button (maximum of 3 points);

Write a standard sentence, for example, ‘our national bird is the bald eagle’ (1 point);

Count backwards from 100 in tens (1 point).

Papilloedema grading is performed according to the modified Frisén scale (Frisen 1982).

Table 15A: Management and Follow-up of Neurological AEs

**Management and Follow-up of Neurological AEs.**	
**Grade 1**	Monitor closely. Treat symptomatically.<br>Vigilant supportive care; aspiration precautions; IV hydration<br>Withhold oral intake of food, medicines, and fluids, and assess swallowing<br>Convert all oral medications and/or nutrition to IV if swallowing is impaired<br>Avoid medications that cause central nervous system depression<br>Low doses of lorazepam (0.25 to 0.5 mg IV every 8 hours) or haloperidol (0.5 mg IV every 6 hours) can be used, with careful monitoring, for agitated patients<br>Neurology consultation<br>Fundoscopic exam to assess for papilloedema<br>MRI of the brain with and without contrast; diagnostic lumbar puncture with measurement of opening pressure; MRI spine if the patient has focal peripheral neurological deficits; CT scan of the brain can be performed if MRI of the brain is not feasible<br>Daily 30 min EEG until toxicity symptoms resolve; if no seizures are detected on

Lee L, et al. J Immunother Cancer 2023; 11:e006699. doi: 10.1136/jitc-2023-006699

**Management and Follow-up of Neurological AEs.**	
	EEG.<br>Consider levetiracetam 750 mg every 12 hours (oral or IV) for a month.<br>If EEG shows non-convulsive status epilepticus, treat as per algorithm in Table 15B.<br>Consider anti-IL-6 therapy with tocilizumab 8 mg/kg IV or siltuximab 11 mg/kg IV, if neurotoxicity is associated with concurrent CRS.<br>**Worsening**: treat as ≥Grade 2
**Grade 2**	Monitor closely. Supportive care and neurological work-up as indicated for Grade 1.<br>Tocilizumab 8 mg/kg IV or siltuximab 11 mg/kg IV if associated with concurrent CRS.<br>Dexamethasone 10 mg IV every 6 hours or methylprednisolone 1 mg/kg IV every 12 hours if refractory to anti-IL-6 therapy, or for neurotoxicity without concurrent CRS.<br>Consider transferring patient to ICU if neurotoxicity is associated with Grade ≥2 CRS.<br>**Worsening:** treat as Grade 3 to 4
**Grade 3 neurologic toxicities, with the exception of headaches, that last continuously for 24 hours or longer**	Supportive care and neurological work-up as indicated for Grade 1 if not done already.<br>ICU transfer is recommended<br>Anti-IL-6 therapy if associated with concurrent CRS, as described for Grade 2 neurotoxicity and if not administered previously.<br>Dexamethasone 10 mg IV every 6 hours or methylprednisolone 1 mg/kg IV every 12 hours if refractory to anti-IL-6 therapy, or for neurotoxicity without concurrent CRS; continue corticosteroids until improvement to Grade 1 neurotoxicity and then taper.<br>Stage 1 or 2 papilloedema with CSF opening pressure <20 mmHg should be treated as per algorithm presented in Table 15C.<br>Consider repeat neuroimaging (CT or MRI) every 2 to 3 days if patient has persistent Grade ≥3 neurotoxicity.<br>Consider using rituximab to activate safety-switch if unresponsive to standard immunosuppressive therapies.
**Grade 4 neurologic toxicity of any duration**<br><br>**Any generalized seizures**	Supportive care and neurological work-up as outlined for Grade 1 neurotoxicity.<br>ICU monitoring; consider mechanical ventilation for airway protection.<br>Anti-IL-6 therapy and repeat neuroimaging as described for Grade 3 neurotoxicity.<br>High-dose corticosteroids continued until improvement to Grade 1 neurotoxicity and then taper; for example, methylprednisolone IV 1 g/day for 3 days, followed by rapid taper at 250 mg every 12 hours for 2 days, 125 mg every 12 hours for 2 days, and 60 mg every 12 hours for 2 days.<br>Consider using rituximab to activate safety-switch if unresponsive to immunosuppressive therapies.<br>For convulsive status epilepticus, treat as per algorithm in Table 15B.<br>Stage ≥3 papilloedema, with a CSF opening pressure ≥20 mmHg or cerebral oedema, should be treated as per algorithm in Table 15C.<br>**Worsening:** May consider use of lymphodepleting drugs such as cyclophosphamide (Garfall et al. 2015) or other drugs (Klinger et al. 2016) if unresponsive to standard immunosuppressive therapies.

Management and Follow-up of Neurological AEs.	
**General**	Patients on IV steroids may be switched to an equivalent dose of oral corticosteroids (e.g. prednisone) at start of tapering or earlier, once sustained clinical improvement is observed.<br>Prophylactic antibiotics or other antimicrobials as clinically appropriate.<br>Rigorous control of blood pressure and electrolytes (particularly calcium and magnesium).

AE=adverse event; CRS=cytokine release syndrome; CSF=cerebrospinal fluid; CT=computerised tomography; EEG=electroencephalogram; ICU=intensive-care unit; IV=intravenous; MRI=magnetic resonance imaging.

Table 15B: Recommendations for the Management of Status Epilepticus after CAR T Cell Therapy

Event	Management
Non-convulsive status epilepticus	Assess airway, breathing, and circulation; check blood glucose.<br>Lorazepam 0.5 mg IV, with additional 0.5 mg IV every 5 min, as needed, up to a total of 2 mg to control electrographical seizures.<br>Levetiracetam 500 mg IV bolus, as well as maintenance doses.<br>If seizures persist, transfer to ICU and treat with phenobarbital loading dose of 60 mg IV.<br>Maintenance doses after resolution of non-convulsive status epilepticus are as follows: lorazepam 0.5 mg IV every 8 hours for three doses; levetiracetam 1000 mg IV every 12 hours; phenobarbital 30 mg IV every 12 hours.
Convulsive status epilepticus	Assess airway, breathing, and circulation; check blood glucose.<br>Transfer to ICU.<br>Lorazepam* 2 mg IV, with additional 2 mg IV to a total of 4 mg to control seizures.<br>Levetiracetam 500 mg IV bolus, as well as maintenance doses.<br>If seizures persist, add phenobarbital treatment at a loading dose of 15 mg/kg IV.<br>Maintenance doses after resolution of convulsive status epilepticus are: lorazepam 0.5 mg IV every 8 hours for three doses; levetiracetam 1000 mg IV every 12 hours; phenobarbital 1–3 mg/kg IV every 12 hours.<br>Continuous electroencephalogram monitoring should be performed, if seizures are refractory to treatment.

ICU=intensive-care unit; IV=intravenous.

Table 15C: Recommendation for Management of Raised Intracranial Pressure (ICP) after CAR T Cell Therapy

Condition	Management
Stage 1 or 2 papilloedema with CSF opening pressure of <20 mmHg without cerebral oedema	Acetazolamide 1000 mg IV, followed by 250 to 1000 mg IV every 12 hours (adjust dose based on renal function and acid–base balance, monitored 1 to 2 times daily).
Stage 3, 4, or 5 papilloedema, with any sign of cerebral oedema on imaging studies, or a CSF opening pressure of ≥20 mmHg	Use high-dose corticosteroids with methylprednisolone IV 1 g/day, as recommended for Grade 4 CAR-T-cell-related encephalopathy syndrome (Table A).<br>Elevate head end of the patient’s bed to an angle of 30 degrees.<br>Hyperventilation to achieve target partial pressure of arterial carbon dioxide ($PaCO_2$) of 28 to 30 mmHg, but maintained for no longer than 24 hours.<br>Hyperosmolar therapy with either mannitol (20 g/dl solution) or hypertonic saline (3% or 23.4%, as detailed below) Mannitol: initial dose 0.5 to 1 g/kg; maintenance at 0.25 to 1 g/kg every 6 hours while monitoring metabolic profile and serum osmolality every 6 hours, and withhold mannitol if serum

Condition	Management
	osmolality is ≥320 mOsm/kg, or the osmolality gap is ≥40.<br>Hypertonic saline: initial 250 mL of 3% hypertonic saline; maintenance at 50 to 75 mL/h while monitoring electrolytes every 4 hours, and withhold infusion if serum Na levels reach ≥155 mEq/l.<br>For patients with imminent herniation: initial 30 mL of 23.4% hypertonic saline; repeat after 15 minutes, if needed.<br>If patient has ommaya reservoir, drain CSF to target opening pressure of <20 mmHg.<br>Consider neurosurgery consultation and IV anaesthetics for burst-suppression pattern on electroencephalography.<br>Metabolic profiling every 6 hours and daily CT scan of head, with adjustments in usage of the aforementioned medications to prevent rebound cerebral oedema, renal failure, electrolyte abnormalities, hypovolemia, and hypotension.

CSF=cerebrospinal fluid; CT=computerised tomography; IV=intravenous.

8.7 MANAGEMENT OF THROMBOCYTOPENIA

Thrombocytopenia is often seen in CAR-treated patients, and in the case of the NCI BCMA CAR study was seen in 2 patients that responded to treatment; in 1 case, thrombocytopenia was severe, requiring platelet transfusions for 9 weeks after infusion of the CAR T cells. Thrombocytopenia is believed to be efficacy-related, due to release of cytokines in the bone marrow, and presumably is seen in myeloma because the tumour mass is largely located in the bone marrow. Efficacy-related symptoms, including CRS, neurotoxicity and thrombocytopenia all have a fast onset and are highly likely to be observed in the first week following CAR T cell infusion. Blood counts should be monitored closely and thrombocytopenia managed as per standard institutional guideline which may include platelet transfusion as necessary.

8.8 MANAGEMENT OF IMMUNE RELATED ADVERSE EVENTS DUE TO ON-TARGET BUT OFF-TUMOUR TOXICITY

Though it is unlikely that AUTO2 will cause immune-related AEs (irAEs) due to on-target but off-tumour toxicity, the patients will be closely monitored for signs and symptoms indicative of irAEs, which may allow for an early recognition of these events. Special attention will be paid to vital organ and irAEs of any grade involving vital organs (e.g. lung, brain, and eyes), more aggressive monitoring and rapid institution of appropriate supportive care should be conducted. In case of severe irAE, not successfully managed by general supportive care, rituximab rescue therapy may be considered.

8.9 RITUXIMAB RESCUE THERAPY

Rituximab will be used in an emergency situation, as rescue medication for any severe grade adverse effects related to AUTO2 to eliminate AUTO2. Rituximab should be used in accordance with this protocol and under the supervision of the Principal Investigator or designee.

Rituximab solution will be prepared for infusion as per local standard of practice and good aseptic practice. The prepared infusion solution of rituximab is physically and chemically stable for up to 24 hours at 2 to 8°C and up to 12 hours at room temperature.

If required, patients will receive a single dose of 375 mg/m^2 of rituximab administered by slow infusion according to manufacturer's guidance for rituximab and the local pharmacy guidelines; ideally, it should be infused at a rate of 50 mg/hour. In the absence of infusion toxicity, the infusion rate should be increased by 50 mg/hour increments every 30 minutes, to a maximum of 400 mg/hour.

At the Principal Investigator's or designee's discretion, this dose may be repeated if there is insufficient depletion of the AUTO2 and the patient's symptoms fail to resolve adequately. It should be noted that rituximab infusions are associated with reactions that are thought to be due to cytokine release. Moreover, rituximab causes B cell depletion and could therefore exacerbate or prolong the immune suppression caused by fludarabine and cyclophosphamide lymphodepletion. Premedication with acetaminophen, antihistamine and methylprednisolone 100 mg IV or its equivalent is recommended 30 minutes prior to each infusion.

Each vial of rituximab is for single use only. Any unused contents and/or vials dispensed for a patient must be destroyed as per local guidelines and recorded in the drug accountability log. Drug accountability logs must be kept for all vials of rituximab dispensed to clinical trial patients. The date of dispensing, the number of vials, expiry date and batch number used/destroyed or returned unused should be recorded. The accountability logs must be verified by the study-assigned Clinical Research Associate during pre-arranged routine monitoring visits.

Depletion of AUTO2 by rituximab is likely to lead to loss of the anti-tumour benefit. The decision to use rituximab should therefore not be made lightly. The following toxicities are considered reasonable indications for RQR8 activation:

- Patients with Grade 3 or 4 CRS that are refractory to tocilizumab, steroids, and anti-TNF antibodies as clinically appropriate.
- Patients with severe on-target, off-tumour toxicity.
- Progressive pathological clonal T cell lymphoproliferative disorders.
- Any other severe, unexpected toxicities attributable to AUTO2 that cannot be managed with conventional measures.

Patients requiring use of rituximab will be considered to have had a DLT or SAE, depending on its occurrence in the Phase I or II part of the study respectively. Following rituximab administration, the patient will continue to be monitored in the study for resolution of the toxicity and for efficacy like any other patient experiencing a DLT. Patients will only be withdrawn early if they meet the early discontinuation criteria as described in Section 3. The depletion of CAR T cells will also be monitored.

9 STUDY VISITS AND ASSESSMENTS

9.1 STUDY OVERVIEW

9.1.1 Screening Phase

All patients must sign an informed consent form prior to the conduct of any study-related procedures. The Screening phase begins when the first screening assessment is conducted.

During the Screening phase, eligibility criteria will be reviewed, demographic data obtained, and a complete clinical evaluation will be performed. Screening procedures will be performed up to 12 weeks before AUTO2 is administered (Schedule of Assessments). If an assessment was performed as part of the patient's routine clinical evaluation and not specifically for this study, it does not need to be repeated after signed informed consent has been obtained provided that the assessments fulfil the study requirements and are performed within the specified timeframe prior to the ATIMP administration. Retesting of abnormal screening values that lead to exclusion is allowed during the screening phase (to reassess eligibility). The patients' last results obtained prior to administering the pre-conditioning chemotherapy will be used to determine eligibility. The measurements collected at the time closest to, but prior to, the administration of AUTO2 will be defined as the baseline values for safety assessment and treatment decisions. Adverse events associated with screening procedures will be collected. Baseline data will be collected during screening as described in the Schedule of Assessments.

Intervening/bridging anti-cancer therapy administered during the screening period is permitted. Where clinically feasible, an anti-CD38 antibody and IMiD combination regimen is to be preferred otherwise bridging therapy will be at the discretion of the investigator as it is compliant with the eligibility criteria and the defined washout periods.

9.1.2 Leukapheresis Phase

The Leukapheresis Phase will begin with venepuncture for the collection of PBMCs. Patients will undergo an unstimulated leukapheresis for the generation of the AUTO2, following provision of informed consent and confirmation of registration into the study. Details of the leukapheresis procedure are provided in Section 7.1. During this period, the assessments described in the Schedule of Assessments will be performed.

If a patient is unable to receive treatment as per original schedule after successful leukapheresis and manufacture of AUTO2 but subsequently re-screens and is eligible for treatment, repeat leukapheresis and manufacturing of AUTO2 may not be necessary and should be discussed with the Sponsor.

9.1.3 Pre-conditioning Phase

Patients that still meet eligibility requirements for the study will proceed to receive a lymphodepleting pre-conditioning treatment with cyclophosphamide and fludarabine for 3 days (starting Day -6), timed to end 4 days (±1 day) before AUTO2 infusion. The pre-conditioning phase will end with the beginning of treatment with AUTO2 infusion. During this phase, AEs associated with pre-conditioning chemotherapy as well as use of concomitant medications will be collected.

9.1.4 Treatment Phase

The Treatment Phase will involve infusion of AUTO2 on Day 0 and will extend until the completion of DLT period (28 days afterAUTO2 infusion). The patient will be admitted to hospital prior to, or on the day of, infusion for up to 10 days for monitoring and management of CRS (longer if clinically necessary). Following infusion, the patient will be closely monitored as described in Section 7.3.3. The patient may be discharged from hospital when clinically appropriate or following successful management of CRS, even if it is prior to 10 days if deemed appropriate by the treating Investigator.

Following discharge from the hospital, the patient will be closely monitored for AEs and laboratory abnormalities, at least on a weekly basis. The required study procedures and assessments to be conducted during the Treatment Stage are outlined in the Schedule of Assessments. Clinical evaluations and laboratory studies may be repeated more frequently, if clinically indicated. Provided that all criteria are satisfied (see Section 7.3.6), patients may receive re-treatment of AUTO2. The patients will transition to the follow-up stage after completion of the DLT evaluation period. Safety and efficacy assessments described in the Schedule of Assessments will be performed and AEs and concomitant medications will be documented. The AUTO2 administration does not require the presence of a representative of the Sponsor.

9.1.5 Follow-up Phase

Upon completion of the Treatment Phase, patients will enter the Follow-up Phase where they will continue to be followed for assessment of AEs and anti-tumour response until the End of Study. End of study will occur at the 2-year anniversary (±14 days) of the start of treatment, or at the time of disease progression, or withdrawal of consent. In the event that the patient is having a treatment related AE or SAE at the planned End of Study, then they can be further followed for up to a maximum period of 6 months or until it stabilises, or death, or withdrawal of consent or start of new treatment, whichever happens first. Laboratory assessments and visits for monitoring will be undertaken as clinically appropriate. Grade 3 or higher AEs that are considered related to the study medication will be recorded/reported as detailed in Section 10.3.

9.1.6 Long-term Follow-up

Provided that patients signed an informed consent form, they will be enrolled on to a long-term follow-up protocol after completion of the study. They will be monitored for SAEs considered related to the study for a period of up to 15 years from the first AUTO2 infusion. The long-term follow-up protocol will be available prior to dosing the first patient.

Note: Patients who have received AUTO2 and have discontinued or completed the study may continue to be monitored for AUTO2 treatment-related SAEs and AEs until they enrol onto the long-term follow-up study (AUTO-LT1) and the End of Study visit will be delayed.

9.2 DETAILS OF STUDY ASSESSMENTS

9.2.1 Demographic and Baseline Assessments

The following will be collected at screening to determine eligibility and baseline status of the patient. Baseline safety assessments will also be performed as detailed in Section 9.2.2.

9.2.1.1 Demographic Data and Baseline Variables

Demographic data will include self-reported race/ethnicity, age, gender, and height at screening visit prior to leukapheresis.

9.2.1.2 Medical/Multiple Myeloma History

Medical history includes clinically significant diseases, surgeries, MM history (including all prior cancer therapies and procedures), and all medications (e.g. prescription drugs, over-the-counter drugs, herbal/homeopathic remedies, and nutritional supplements) used by the patient. Histological confirmation of disease diagnosis will be obtained (pathology report) and results of cytogenetic markers analysis will be collected.

9.2.1.3 Pregnancy Test

Serum (β-human chorionic gonadotropin) or urine pregnancy testing will be performed for women of childbearing potential at the screening visit and will be repeated as described in the Schedule of Assessments.

9.2.2 Safety Evaluations

All patients who receive AUTO2 will be considered evaluable for toxicity assessment. Any clinically relevant changes occurring during the study must be recorded on the AE section of the eCRF. Safety assessments will be based on medical review of AE reports and the results of vital sign measurements, ECGs, physical examinations, clinical laboratory tests and ECOG performance status at specified time points as described in the Schedule of Events. Any clinically significant abnormalities persisting at the end of the study/early withdrawal will be followed by the Investigator until resolution, or until a clinically stable endpoint is reached. The study will be monitored by the SEC; details regarding the SEC are provided in Section 10.5. The study will include the following evaluations of safety and tolerability according to the time points provided in the Schedule of Assessments.

9.2.2.1 Adverse Events

Adverse events will be noted by clinic staff or reported by the patient (or, when appropriate, by a caregiver, surrogate, or the patient's legally acceptable representative) for the duration of the study. Adverse event recording and reporting is described in detail in Section 10.3. Toxicity will be graded using the NCI CTCAE Version 4.03 criteria. The incidence of AEs will be tabulated and reviewed for potential significance and clinical importance.

9.2.2.2 Clinical Laboratory Tests

Blood samples for haematology, coagulation and biochemistry will be collected at each visit as specified in the Schedule of Assessments. Where appropriate, tests must be performed prior to receiving pre-conditioning treatment or AUTO2 infusion. More frequent clinical laboratory tests may be performed if indicated by the overall clinical condition of the patient or by abnormalities that warrant more frequent monitoring. Screening laboratory results must be available to the Investigator for evaluation before AUTO2 infusion and subsequent laboratory results should be available at the time of the patient's evaluation by the treating physician. The Investigator must review the laboratory reports, document this review, and ensure that any clinically relevant changes occurring during the study are recorded in the AE section of the eCRF. The laboratory reports must be filed with the source documents. A summary of the tests that will be performed by the local laboratory is presented in Table 16.

Lee L, et al. J Immunother Cancer 2023; 11:e006699. doi: 10.1136/jitc-2023-006699

Table 16: Clinical Laboratory Tests

Assessment	Description
Haematology	Haemoglobin, red blood cell count, platelet count, white blood cell count with differential (neutrophils, eosinophils, lymphocytes, monocytes, and basophils).
Coagulation	Prothrombin time, international normalised ratio, activated PTT, fibrinogen.
Biochemistry	Sodium, phosphate, potassium, ALT, AST, creatinine, CPK, lactate dehydrogenase, total bilirubin, calcium, total protein, albumin, glomerular filtration rate and creatinine clearance performed only at screening.
CRP and Ferritin	Ferritin and C-reactive protein.
Pregnancy test	Serum (β-human chorionic gonadotropin) or urine pregnancy testing for women of childbearing potential.
Serology	• Human immunodeficiency virus antibody.<br>• Hepatitis B core antibody: if positive, further testing (deoxyribonucleic acid by PCR) to rule out active disease or chronic carrier. Must be confirmed negative prior to screening.<br>• Hepatitis C virus antibody: if positive for hepatitis C virus, further testing (by RNA PCR) should be performed to rule out active infection.<br>• Anti-human T-lymphotropic virus-1.<br>• Anti-human T-lymphotropic virus-2.<br>• Syphilis Serology.

ALT=alanine aminotransferase; AST=aspartate aminotransferase; CPK=creatine phosphokinase; PCR=polymerase chain reaction; PTT=partial thromboplastin time; RNA=ribonucleic acid.

9.2.2.3 12-lead Electrocardiogram

A 12-Lead ECG will be obtained using an ECG machine that automatically calculates the heart rate and measures PR, RR, QRS, QT, and corrected QT intervals. Refer to the Schedule of Assessments for details regarding the frequency of ECG assessments. At each time point, a single 12-lead ECG will be performed by qualified site personnel. The clinical Investigator will review the printout, including ECG morphology. The ECG should be repeated in triplicate if motion artefacts or clinically relevant abnormalities are noted. Additional cardiovascular assessments should be performed as clinically appropriate to ensure patient safety. If blood sampling or vital sign measurement is scheduled for the same time point as ECG recording, the procedures should be performed in the following order: ECG(s), vital signs, blood draw.

9.2.2.4 Echocardiogram (ECHO) or Multiple Gated Acquisition (MUGA)

Echocardiogram is the preferred method to assess cardiac ejection fraction and cardiac valve abnormalities; MUGA is an acceptable alternative. Assessments should be performed at screening and should include an evaluation for left ventricular ejection fraction and both right- and left-sided valvular lesions. Additional ECHO/MUGA assessments may be performed as clinically indicated. Generally, for a patient, the same procedure should be performed at screening and at any follow-up assessment to allow direct comparison.

9.2.2.5 Vital Signs

Vital signs will include temperature, pulse/heart rate, respiratory rate, blood pressure [systolic and diastolic] and weight. Blood pressure and pulse/heart rate measurements should be recorded with the patient in a seated position. Multiple time points (minimum of 3) will be

Lee L, *et al. J Immunother Cancer* 2023; 11:e006699. doi: 10.1136/jitc-2023-006699

collected prior to treatment to establish a good baseline blood pressure for the patient. Blood pressure and pulse/heart rate measurements will be assessed with a completely automated device. Manual techniques will be used only if an automated device is not available.

9.2.2.6 Physical Examination

A complete physical examination will be conducted at screening as per the institutional standard practice. Thereafter, a symptom-directed physical examination will be conducted at subsequent visits if indicated. From Day 0 onwards, only clinically significant observations will be recorded and reported as AEs as appropriate. The schedule for physical examinations is provided in the Schedule of Assessments.

9.2.2.7 ECOG Performance Status

The ECOG scale provided in Appendix 2 will be used to grade changes in the patient's daily living activities. The frequency of ECOG assessments is provided in the Schedule of Assessments.

9.2.3 Pharmacodynamics and Biomarker Evaluation

Blood-based pharmacodynamics biomarkers will be evaluated in all patients as described in the Schedule of Assessments. Peripheral and bone-marrow biomarkers may be assessed pre- and post-treatment with AUTO2. Assessment at additional or fewer parameters and/or time points may be performed based on emerging data.

9.2.3.1 Evaluation of AUTO2 Persistence in Peripheral Blood

Two validated assays will be used to measure the expansion/persistence of RQR8/APRIL CAR T cells in the peripheral blood following agreed Standard Operating Procedures at time points indicated in the Schedule of Assessments. Flow cytometry will be used to measure the frequency of RQR8/APRIL CAR T cells per microliter of whole blood and a qPCR assay will be used to quantify the number of copies of the RQR8/APRIL CAR transgene per 100 cells in peripheral whole blood. Sample analysis may cease when the results of 2 consecutive tests are negative. Thereafter, if the patient has not experienced disease progression, samples will be collected and stored. Please refer to the AUTO2-MM1 laboratory manual for the handling and storage of samples.

9.2.3.2 Evaluation of RCR in Peripheral Blood

As per health authorities' guidelines, tests will be performed to evaluate and monitor the presence of replication competent retrovirus by qPCR in whole blood. Please refer to the AUTO2-MM1 laboratory manual for the handling and storages of samples.

9.2.3.3 Insertional Mutagenesis

Insertional mutagenesis leading to oncogenesis is a recognised safety concern of vector-based therapy. Samples will be stored and archived with the intent to be analysed for insertional mutagenesis, should a patient develop a new malignancy. The result will allow a potential relationship between AUTO2 treatment and the development of any new malignancy to be established.

9.2.3.4 Exploratory Biomarker Assessments

Serum cytokine profile: (minimum dataset of TNF-α, IFN-γ, and IL-6) will be measured using a highly sensitive, reproducible and validated cytokine assay at time points indicated in the Schedule of Assessments. Additional samples may be taken where clinically indicated, for example during CRS, samples should be collected daily. Serum samples for cytokine measurements may be frozen and batched for analysis or assayed using fresh serum. Serum may be used to for measurement of other biomarkers such as soluble BCMA and TACI or additional biomarkers as appropriate.

Immunological phenotyping/genotyping: Peripheral blood mononuclear cells may be isolated from whole blood following standard procedures and cryopreserved in liquid nitrogen for later immunological assessment or assessed immediately. Samples will not be stored for more than 15 years. Peripheral blood mononuclear cells may be used for various immunological assessments such as phenotyping by flow cytometry, genomic analysis and other assays as developed. Immunophenotyping or genotyping will be evaluated at selected time points dependent upon a minimum frequency of AUTO2 cells evaluated in the secondary assessments.

BCMA and TACI expression on bone marrow plasma cells: The expression of BCMA and TACI in bone marrow plasma cells will be evaluated by IHC and/or flow cytometry according to agreed Standard Operating Procedures and methods.

If the patient is declared to have disease progression outside of scheduled BM biopsy time points, optional blood and bone marrow samples will be collected to elucidate the reasons for primary or acquired resistance using appropriate methods.

AUTO2 persistence in bone marrow: A portion of the bone marrow aspirate may be used to evaluate the extent of AUTO2 cell infiltrate/persistence and to investigate potential markers of drug sensitivity and or resistance and disease progression. These may include immune profiling of bone marrow mononuclear cells. Evaluation of RQR8/APRIL CAR T cells in bone marrow samples may be performed by flow cytometry or qPCR or other appropriate assay. Please refer to the AUTO2-MM1 laboratory manual for the handling and storage of samples.

9.2.3.5 Immunogenicity Analysis

Detection of human anti-CAR antibodies, or related antibodies, may be measured in serum. No additional blood will be collected for the detection of these antibodies as serum frozen for cytokine measurements can be utilised for this assay. Serum samples at selected time points, for example at Day 0, End of DLT evaluation period and M3, may be analysed if clinically indicated or for publication purposes. The assay has not yet been developed but will be based on assays used to measure human anti-human antibodies in patients treated with monoclonal antibodies. The development of the assay may utilise serum collected during the trial and data will not be reported if a suitable assay cannot be developed.

9.2.4 Efficacy Evaluation

Response evaluations for the primary endpoint/final analysis will be based on assessments, using 2016 IMWG Guidelines.

9.2.4.1 Response Categories

Disease evaluations must be performed at screening, on the day of AUTO2 administration and then at visits as outlined in the Schedule of Assessments. Disease evaluation assessments will be performed by a local laboratory until disease progression. This study will use the IMWG consensus recommendations for MM treatment response criteria (Durie 2006, Rajkumar et al. 2011, Kumar et al. 2016). New abnormalities or changes from screening assessments should be recorded in the patient's medical notes and should also be recorded on the corresponding eCRF page.

Disease response: The International Myeloma Working Group uniform response criteria for MM will be employed for documenting disease response, as presented in Appendix 1.

Disease progression must be consistently documented across clinical study sites using the criteria in Appendix 1. It is important that instances and evidence of disease progression (diagnosis and documentation) be reported to the Sponsor as soon as possible. The medical monitor will review the data to confirm that the IMWG criteria for disease progression have been met. If the medical monitor concurs, the Investigator will be notified and the patient will be withdrawn from the study. If the medical monitor considers that the IMWG criteria for disease progression have not been met, then the Sponsor will contact the Investigator to discuss the patient. For continuation in the study, the IMWG response will be determined on an ongoing basis by the Investigator.

Serum free light chain assay test results will be analysed by the local laboratory for the assessment of sCR, according to the most recently published IMWG criteria (Durie 2006). Minimal residual disease (MRD) will be assessed by flow cytometry or other appropriate methods at a central laboratory.

9.2.4.2 Myeloma Protein Measurements in Serum and Urine

Blood and 24-hour urine samples for M-protein measurements will be analysed by the local laboratory. Only one serum and one 24-hour urine sample per time point are required to perform the following tests:

- Serum quantitative immunoglobulins (QIgs): All patients will be evaluated for IgG, IgA, IgM at screening. To be repeated as clinically indicated.
- Serum M-protein quantitation by serum protein electrophoresis (SPEP) at screening visits, on Day 0 and then repeated at every visit from the end of the DLT period onwards.
- Serum immunofixation at Screening and thereafter when a CR is suspected.
- Serum free light chain assay: This test will be performed at screening visits, on Day 0 and then repeated at every visit from the end of the DLT period onwards for patients who are serum free light chain positive i.e. light chain positive MM or when CR is suspected or maintained.
- Twenty-four hour-urine M-protein quantitation by urine protein electrophoresis (UPEP) at screening visits, on Day 0 and then repeated at every visit from the end of the DLT period onwards.
- Urine immunofixation at Screening and thereafter when a CR is suspected.

Blood and 24-hour urine samples will be collected as specified in the Schedule of Assessments until the development of confirmed disease progression. Serum and urine immunofixation test and serum free light chain assay will be performed at Screening and

thereafter when a CR is suspected (when serum or 24-hour urine M-protein electrophoresis [by SPEP or UPEP] are 0 or non-quantifiable). For patients with light chain MM, both serum and urine immunofixation test and serum free light chain assay will be performed routinely.

9.2.4.3 Serum Calcium Corrected for Albumin

Blood samples for calculating serum calcium corrected for albumin will be collected and analysed locally, as specified in the Schedule of Assessments, until the development of confirmed disease progression. Development of hypercalcemia (corrected serum calcium >11.5 mg/dL or 2.8 mmol/L) can indicate disease progression or relapse if it is not attributable to any other cause (Appendix 1). Calcium binds to albumin and only the unbound (free) calcium is biologically active; therefore, the serum calcium level must be adjusted for abnormal albumin levels ("corrected serum calcium"). If blood is analysed at a local laboratory, measurement of free ionised calcium is an acceptable alternative to corrected serum calcium for determining hypercalcemia. Free ionised calcium levels greater than the ULN (local laboratory reference ranges) are considered to be hypercalcaemic for this study.

9.2.4.4 β2-microglobulin and Albumin

Blood samples for β2 microglobulin and albumin are to be collected at screening and will be analysed by the local laboratory.

9.2.4.5 Bone Marrow Examination

Bone marrow will be sampled for MRD evaluation during one of the screening visits to establish baseline MRD (to obtain patient specific phenotype) and for morphological analysis to establish the percentage of plasma cells. An additional bone marrow sample will be collected at the end of the DLT evaluation period visit and at M3. Patients remaining in biochemical CR may undergo further bone marrow sampling at M6, M12, M18 and M24 in order to confirm continued CR. Bone marrow aspirate will be analysed for MRD by flow cytometry or other appropriate methods. Please refer to the AUTO2-MM1 laboratory manual for the handling and storage of samples.

9.2.4.6 Fluorodeoxyglucose PET/CT Imaging

A PET-CT assessment of skull, ribs, upper limbs, femurs, pelvis and spine will be completed at baseline to accurately assess osteolytic lesions and the extent of hypermetabolic activity in intramedullary and extramedullary sites during screening.

Positron emission tomography-computerised tomography scans may be repeated when clinically indicated to document response or progression. Computerised tomography (CT) or MRI is an acceptable method for evaluation of bone disease, and may be included at the discretion of the Investigator.

Disease progression may be manifested by pain related to osteolytic bone disease. Therefore, disease progression may be documented, in these cases, by appropriate radiological investigations. If the diagnosis of disease progression is obvious by radiographic investigations, then no repeat confirmatory radiography is necessary. In instances where changes may be more subtle, repeat radiology at an appropriate time point may be required.

9.2.4.7 Documentation of Extramedullary Plasmacytomas

Sites of known extramedullary plasmacytomas must be documented at screening. Clinical examination or cross-sectional imaging (MRI, CT, PET-CT) may be used to document

extramedullary sites of disease. The modality used for follow-up scans for disease response assessment should be consistent with initial/baseline techniques used. Positron emission tomography-computerised tomography is recommended for functional assessment, however, MRI (spine and/or pelvis) and CT may be used according to local practice.

Extramedullary plasmacytomas should be assessed for all patients with a history of plasmacytomas or if clinically indicated at screening by radiological imaging, and repeated as clinically indicated during treatment to confirm response or disease progression. If assessment can only be performed radiologically, then evaluation of extramedullary plasmacytomas may be done every 12 weeks.

For every patient, the methodology used for evaluation of each disease site should be consistent across all visits. Irradiated or excised lesions will be considered unmeasurable, and will be monitored only for disease progression.

To qualify for PR or MR, the sum of products of the perpendicular diameters of the existing extramedullary plasmacytomas must have decreased by at least 50% or 25%, respectively, and new plasmacytomas must not have developed (see Appendix 1). To qualify for disease progression, either the sum of products of the perpendicular diameters of the existing extramedullary plasmacytomas must have increased by at least 50%, or a new plasmacytoma must have developed. In the cases where not all existing extramedullary plasmacytomas are reported, but the sum of products of the perpendicular diameters of the reported plasmacytomas have increased by at least 50%, this will also qualify as disease progression.

9.2.5 Blood Volume Collections

The total estimated volume of blood collected for safety, biomarkers and immunological assessments (with the exception of the leukapheresis procedure) across the entire study will not normally exceed 665 mL. The maximum volume of blood collected on any 1 day will unlikely exceed 50 mL. No more than 300 mL of blood will be collected in any 28-day period. Additional samples may be collected as required to ensure the safety of the patient, or for additional cytokine measurements, which are not included in these estimates. Refer to the laboratory manual for the handling and storages of samples.

10 SAFETY MANAGEMENT

Timely, accurate, and complete reporting and analysis of safety information from clinical studies are crucial for the protection of patients, investigators, and the Sponsor, and are mandated by regulatory agencies worldwide. The Investigator or site staff will be responsible for detecting, documenting and reporting events that meet the definition of an AE or SAE.

10.1 ADVERSE EVENTS DEFINITIONS

10.1.1 Adverse Event

According to the International Conference on Harmonisation (ICH) guideline for Good Clinical Practice (GCP), an AE is any untoward medical occurrence in a clinical investigation patient administered a pharmaceutical product, regardless of causal attribution. An AE can therefore be any of the following:

- Any untoward medical occurrence in a patient or clinical trial subject administered a medicinal product and which does not necessarily have a causal relationship with this treatment.
- Any new disease or exacerbation of an existing disease (a worsening in the character, frequency, or severity of a known condition).
- Recurrence of an intermittent medical condition (e.g. headache) not present at baseline.
- Any deterioration in a laboratory value or other clinical test that is considered clinically relevant, may lead to a change in concomitant medication or management, or leads to a change in study treatment, concomitant treatment or discontinuation from the study.
- Adverse events that are related to a protocol-mandated intervention, including those that occur prior to assignment of study treatment (e.g. pre-conditioning or screening invasive procedures such as biopsies).

Events that do **NOT** meet the definition of an AE include:

- Medical or surgical procedure (e.g. endoscopy, appendectomy); the condition that leads to the procedure is an AE.
- Situations where an untoward medical occurrence did not occur (social and/or convenience admission to a hospital).
- Anticipated day-to-day fluctuations of pre-existing disease(s) or condition(s) present or detected at the start of the study that do not worsen.
- The disease/disorder being studied (i.e. MM), or expected progression, signs, or symptoms of the disease/disorder being studied, unless more severe than expected for the patient's condition.

10.1.2 Adverse Reactions

An adverse reaction is defined as any untoward and unintended responses to an investigational medicinal product related to any dose administered.

An unexpected adverse reaction is an adverse reaction in which the nature or severity of which is not consistent with the Investigator Brochure for AUTO2.

10.1.3 Serious Adverse Events

An SAE is any AE that meets any of the following criteria:

- Fatal (i.e. the AE actually causes or leads to death). Death due to disease progression will not be considered as an SAE.
- Life threatening (i.e. the AE, in the view of the Investigator, places the patient at immediate risk of death). This does not include any AE that, had it occurred in a more severe form, or was allowed to continue, might have caused death.
- Requires inpatient hospitalisation (other than pre-planned hospitalisation including any hospitalisation for study treatment and hospitalisation for disease progression) or prolongation of existing hospitalisation due to an AE.
- Results in persistent or significant disability/incapacity (i.e. the AE results in substantial disruption of the patient's ability to conduct normal life functions).
- Congenital anomaly/birth defect in a neonate/infant born to a mother exposed to the study drug.
- It is a suspected transmission of any infectious agent via a medicinal product.
- Significant medical event in the Investigator's judgment (e.g. may jeopardise the patient or may require medical/surgical intervention to prevent 1 of the outcomes listed above).

Protocol-specific SAEs are as follows:

- Grade 4 CRS.
- Any new primary cancers.
- Significant cardiac dysfunction such as Grade 3 or higher decrease in left ventricular ejection fraction.

Serious AEs are required to be reported by the Investigator to the Sponsor immediately (i.e. no more than 24 hours after learning of the event; see Section 10.3.2 for reporting instructions).

Events <u>NOT</u> considered as SAEs are hospitalisations for:

- A procedure for protocol/disease-related investigations (e.g. surgery, scans, endoscopy, sampling for laboratory tests, bone marrow sampling). However, hospitalisation or prolonged hospitalisation for a complication of such procedures remains a reportable SAE.
- Extension of routine in-patient hospital stay beyond the recommended 10 days following AUTO2 infusion for general management purposes or until resolution of AEs, or if it is standard process for the management of underlying disease (including the re-admission for close monitoring in a setting such as an increase or expansion of RQR8/APRIL CART cells).
- Hospitalisation for administration of pre-conditioning chemotherapy or conducting study procedures.
- Routine treatment or monitoring of the studied indication not associated with any deterioration in condition.

- The administration of blood or platelet transfusion as routine treatment of studied indication. However, hospitalisation or prolonged hospitalisation for a complication of such transfusion remains a reportable SAE.
- Hospitalisation or prolongation of hospitalisation for technical, practical, or social reasons, in absence of an AE.
- Hospitalisations not intended to treat an acute illness or AE (e.g. social reasons such as pending placement in a long-term care facility).
- A procedure that is planned (i.e. planned prior to starting of treatment on study) must be documented in the source document and the Case Report Form (CRF). Hospitalisation or prolonged hospitalisation for a complication remains a reportable SAE.
- An elective treatment of a pre-existing condition unrelated to the studied indication.
- Emergency outpatient treatment or observation that does not result in admission, unless fulfilling other seriousness criteria above.
- An event which is part of the natural course of the disease under study (i.e. disease progression or hospitalisation due to disease progression, or pain control, stabilisation of fractures) does not need to be reported as an SAE. Death due to the disease under study is to be recorded on the Death CRF form. However, if the underlying disease (i.e. progression) is greater than that which would normally be expected for the patient, or if the Investigator considers that there was a causal relationship between treatment with study medication(s) or protocol design/procedures and the disease progression, then this must be reported as an SAE.

10.1.4 Suspected Unexpected Serious Adverse Reactions

A suspected unexpected serious adverse reaction (SUSAR) is any suspected adverse reaction related to the study treatment that is both unexpected and serious, even if it is a component of the study endpoint (e.g. all-cause mortality).

10.1.5 Adverse Events of Special Interest

The following are AEs of special interest.

- Grade 2 to 5 CRS.
- Grade 2 to 5 neurotoxicity (including depressed level of consciousness, dysphagia, ataxia, seizures, and cerebral oedema).

AEs of special interest that are ≥Grade 3 should be reported to the sponsor within 24 hours of becoming aware of the event. (see Section 10.3.2 for reporting instructions). Additional information may be gathered about these AEs. Based on emerging data, additional AEs may be classified as AEs of special interest.

10.2 ADVERSE EVENTS ASSESSMENT

Adverse event information will be collected at study visits. Patients will be instructed to call study site personnel to report any abnormalities during the intervals between study visits, and to come to the study site if medical evaluation is needed and the urgency of the situation permits. Adverse events will be assessed by the Investigator, or appropriately qualified

Lee L, *et al. J Immunother Cancer* 2023; 11:e006699. doi: 10.1136/jitc-2023-006699

designee, for severity, relationship to study treatment, action taken, outcome and whether the event meets criteria as an SAE according to the guidelines presented in Table 17.

10.2.1 Severity of Adverse Events

The severity of AEs will be graded based upon the patient's symptoms according to the NCI CTCAE (Version 4.03).

Adverse events that are not defined in the NCI CTCAE should be evaluated for severity according to the following scale.

Table 17: Severity Grading of AEs Not Listed on the NCI CTCAE Grading System

Grade	Severity	
1	Mild	Transient or mild discomfort; no limitation in activity; no medical intervention/therapy required.
2	Moderate	Mild to moderate limitation in activity, some assistance may be needed; no or minimal medical intervention/therapy required.
3	Severe	Marked limitation in activity, some assistance usually required; medical intervention/therapy required, hospitalisation is possible.
4	Life-threatening	Extreme limitation in activity, significant assistance required; significant medical intervention/therapy required, hospitalisation or hospice care probable.
5	Fatal	Death as a result of this AE.

AE=adverse event.

Note: It is important to distinguish between serious and severe AEs. Severity is a measure of intensity whereas seriousness is defined by the criteria in Section 10.2.1. Seriousness, not severity, serves as a guide for defining regulatory obligations.

10.2.2 Assessment of Relationship

The Investigator must determine the relationship between the administration of the study drug and the occurrence of an AE/SAE as defined below:

Relationship assessments that are classed as "Not Related" to study treatment:

Not Related: The AE is not related to the investigational product.

The patient either did not receive the investigational product or the event is related to an aetiology other than the investigational product (the alternative aetiology must be documented in the study patient's medical record).

Unlikely Related: The AE is doubtfully related to the investigational product.

The event is not clearly related to an identified aetiology other than the investigational product; but there is no plausible mechanism for the event to be related to the investigational product and/or there is no clear association between the event and the administration of the investigational product.

Relationship assessments that are classed as "Related" to study treatment:

Possibly Related: The AE may reasonably be related to the investigational product.

Probably Related: The AE is likely to be related to the investigational product.

There is an association between the event and the administration of the investigational product, there is a plausible mechanism for the event to be related to the investigational product, the event is less likely to be explained by known characteristics of the patient's clinical status, and an alternative aetiology is not apparent.

Definitely Related: The AE is clearly related to the investigational product.

If an event is assessed as suspected of being related to a comparator, ancillary, or additional study drug that has not been manufactured or provided by the Sponsor, please provide the name of the manufacturer when reporting the event.

10.3 ADVERSE EVENTS REPORTING PROCEDURES

10.3.1 All Adverse Events

All AEs, both those observed by study site personnel and those spontaneously reported by the patient, will be recorded in the eCRF. All AEs, irrespective of seriousness, severity, or relationship to study drug, must be recorded using medical terminology that accurately reflects the event. Whenever possible, diagnoses should be given when signs and symptoms are due to a common aetiology. The relationship of the AE to study therapy must be recorded in the eCRF by the Investigator or designee. All measures required to manage an AE must be recorded in the source document and reported according in the eCRF as per eCRF completion guidelines.

Note: Grade 1 to 2 haematology laboratory abnormalities can be frequent in this patient population and are not subject to reporting unless felt to be clinically significant by the investigator.

The reporting period for all AEs is described in Table 18.

Table 18: Reporting Period for All AEs

**From**: Informed consent form signature<br>**Until**: Day 60 post last AUTO2 infusion[a]	**From**: Day 60 post last AUTO2 infusion<br>**Until**: End of study[a]
• All AEs from Day -7<br>• AEs related to study procedure **only for 48 hours** following the procedure (e.g. leukapheresis, bone marrow biopsy)<br>• All SAEs from Day -7	• Treatment-related Grade 3 to 5 AEs<br>• AEs of special interest (Section 10.3.3)<br>• AEs related to study procedure (e.g. bone marrow biopsy)<br>• Treatment-related SAEs

AE=adverse event; SAE=serious adverse event.
a Or until withdrawal whichever occurs first.

Adverse events related to intervening/bridging non-study related anti-cancer therapy administered prior to pre-conditioning or AEs associated with disease progression during the same period will not be reported as AEs. These events will be recorded as an update to the medical history of the patient (since signing consent). Please refer to eCRF completion guidelines.

The Investigator should follow each AE until the event has resolved to baseline or Grade 1, the event is assessed as stable by the Investigator, the patient is lost to follow-up or the patient withdraws consent.

In the event that a drug-related AE and/or any SAE is ongoing at the time of proposed completion of the study (2 years after dosing or at progression, whichever happens first) then the event will be further followed up until the AEs are resolved or stabilised for a maximum of 6 months, or death, withdrawal of consent, start of new treatment or until patient contact discontinues, whichever happens first (extended end of study). During the extended follow-up period, the following will be collected:

- Resolution information for ongoing AEs from the study treatment period
- New treatment related AEs only

10.3.2 Serious Adverse Events

All SAEs occurring during the study must be reported to the appropriate Sponsor contact person by study-site personnel within 24 hours of their knowledge of the event. Additional or follow-up information on a SAE must also be reported immediately (i.e. within 24 hours of awareness of the SAE). This timeframe also applies to additional new information (follow-up) on previously forwarded SAEs. Should the FDA or other regulatory authority require that the Sponsor submit additional data on the event, the Investigator will be asked to provide those data to the Sponsor in a timely fashion.

Information to be Provided by the Investigator for an SAE:

Information regarding an SAE will be transmitted to the Sponsor using the Serious Adverse Event Form, which must be completed and signed by a physician from the study site, and transmitted to the Sponsor within 24 hours. The Sponsor or designee will require all of the following information about the patient and the event:

- Investigator identification
- Patient identification code
- Information on study treatment (e.g. start/stop date, dose and frequency of study treatment administered)
- Description of event

In addition to the above information, the Sponsor will require the Investigator's assessment of the following:

- Severity of the SAE
- Relationship of the SAE to the study treatment
- Outcome of the SAE

The SAE report form should be completed and faxed or emailed to the relevant toll-free fax number or email address (fax numbers and email address are listed on the form and in the SAE form completion guidelines).

Follow-up Information on an SAE:

Appropriate diagnostic tests should be performed and therapeutic measures, as medically indicated, should be instituted. Serious AEs must be followed up to resolution by the Investigator, even if this extends beyond the study reporting period. Resolution of an SAE is

defined as the return to baseline status, Grade 1, or stabilisation of the condition with the expectation that it will remain chronic. For all SAEs, the Investigator is obligated to pursue and provide information to the Sponsor. In addition, an Investigator may be requested by the Sponsor to obtain specific information in an expedited manner. This information may be more detailed than that captured on the SAE form. In general, this will include a description of the AE in sufficient detail to allow for a complete medical assessment of the case and independent determination of possible causality. Information on other possible causes such as concomitant medication and illnesses must be provided.

After the initial SAE report, the Investigator is required to follow each patient proactively and to report new significant follow-up by submitting an updated SAE report form to the Sponsor (or designee). New significant information includes, but is not limited to, the following:

- New signs or symptoms or a change in the diagnosis
- Significant new diagnostic test results
- Change in causality based on new information
- Change in the event's outcome, including recovery
- Additional narrative information on the clinical course of the event

There should be routine follow-up as clinically appropriate until the event resolves or stabilises or end of study as per criteria described above. The Medical Monitor may specify a longer period of time, if required to assure the safety of the patient.

At the end of study, or following discontinuation, any new toxicities related to the study treatment or any events listed below will be reported and followed up under the long-term follow up protocol:

- All AEs designated as special interest
- Serious AEs related to the study drug and the following:
  - New malignancies
  - Incidence/exacerbation of pre-existing neurologic disorder
  - New incidence or exacerbation of a prior rheumatologic or other autoimmune disorder
  - Others designated special interests
- New incidence of a haematological disorder designated of special interest.

The Investigator should report these events to the Sponsor Drug Safety Provider in writing (if the study database has not yet been closed on the AE eCRF page).

Medical Review and Reporting by the Sponsor:

The Sponsor (or designee) will determine expedited reporting requirements for each reported SAE according to local requirements based upon:

- Causality
- Expectedness

The current IB Section "Reference Safety Information" will be used as the reference for determination of expectedness and risk assessment of AUTO2 related AEs.

Any Serious Adverse Reaction associated with the use of cyclophosphamide and fludarabine used as pre-conditioning will be notified to the appropriate manufacturer.

Sponsor Responsibility for Expedited Safety Reports:

The Sponsor (or designee) will notify Investigators of all reportable SAEs. This notification will be in the form of an expedited safety report. Upon receiving such notices, the Investigator must review and retain the notice with other study-related documentation.

The Sponsor will ensure that SAEs are reported to the IEC/IRB(s) and regulatory authority(ies) according to local requirements.

Suspected unexpected adverse reactions and other significant safety issues reported from the investigational product development program shall be reported to the relevant competent health authorities in all concerned countries according to local regulations (either as expedited safety reports and/or in aggregate reports), by the Sponsor (or designee).

10.3.3 Adverse Events of Special Interest

Adverse events of special interest that are ≥Grade 3, including neurotoxicity and CRS, should be reported to the sponsor within 24 hours of becoming aware of the event.

10.3.4 Management of Pregnancy

Pregnancies

Female patients of childbearing potential will be instructed to immediately inform the Investigator if they become pregnant during the study or within 12 months after the last dose of study treatment. Any pregnancies during this period must be reported to the Sponsor (or designee) within 24 hours of knowledge of the event. The pregnancy report form should be completed and faxed or emailed to the relevant toll-free fax number or email address (fax numbers and email address are listed on the bottom of the form).

While pregnancy itself is not considered to be an AE or SAE, any pregnancy complication or elective termination of a pregnancy for medical reasons will be recorded as an AE or SAE. Abnormal pregnancy outcomes (e.g. spontaneous abortion, foetal death, stillbirth, congenital anomalies, and ectopic pregnancy) are considered SAEs and must be reported using the SAE Form to the Sponsor immediately (i.e. no more than 24 hours after learning of the event).

The Investigator should counsel the patient, discussing the risks of the pregnancy and the possible effects on the foetus. Monitoring of the patient should continue until conclusion of the pregnancy.

Pregnancies in Female Partners of Male Patients

Male patients will be instructed to immediately inform the Investigator if their partner becomes pregnant during the study or within 12 months after the last dose of study treatment. Any pregnancies during this period must be reported to the Sponsor (or designee) within 24 hours of knowledge of the event. The pregnancy report form should be completed and faxed or emailed to the relevant toll-free fax number (fax numbers and email address are listed on the bottom of the form).

Attempts should be made to collect and report details of the course and outcome of any pregnancy in the partner of a male patient exposed to study treatment. The pregnant partner will need to sign an authorisation for use and disclosure of pregnancy health information to allow for follow-up on her pregnancy. The Investigator may provide information on the risks

of the pregnancy and the possible effects on the foetus, to support an informed decision in cooperation with the treating physician and/or obstetrician.

Outcome

Additional information on the course and outcome of the pregnancy should be provided to the Sponsor (or designee) as soon as becoming available (i.e. no more than 24 hours after learning of the event using the paper pregnancy report form. The following pregnancy outcomes will be considered to be SAEs and should be reported according to the procedure in Section 10.3.2.

- Spontaneous abortion (as the Sponsor considers spontaneous abortions to be medically significant events).
- Any congenital anomaly/birth defect in a child born to a female patient or female partner of a male patient.
- All neonatal deaths occurring within 30 days of birth should be reported, without regard to causality, as SAEs.

Follow-up information regarding the outcome of the pregnancy and any postnatal sequelae in the infant will be required. In addition, any infant death after 30 days that the Investigator suspects is related to the in utero exposure to the study drug should also be reported to the Sponsor's Drug Safety Group within 24 hours of the Investigator's knowledge of the event by faxing or emailing a copy of the Pregnancy Report Form to the Sponsor Pharmacovigilance Provider.

10.3.5 Other Significant Events Reporting

Safety events of interest on a Sponsor study drug that may require expedited reporting and/or safety evaluation include, but are not limited to:

- Overdose of a Sponsor study drug
- Suspected abuse/misuse of a Sponsor study drug
- Inadvertent or accidental exposure to a Sponsor study drug
- Medication error involving a Sponsor product (with or without subject/patient exposure to the Sponsor study drug, e.g. name confusion)

A study drug overdose is the accidental or intentional use of AUTO2 in an amount higher than the dose being studied. An overdose or incorrect administration of study drug is not an AE unless it results in untoward medical effects. Any AUTO2 overdose or incorrect administration of AUTO2 should be noted on the corresponding CRF page. All AEs associated with an overdose or incorrect administration of AUTO2 should be recorded on the AE CRF page. If the associated AE fulfils serious criteria, the event should be reported to the Sponsor immediately (i.e. no more than 24 hours after learning of the event; see Section 10.3.2).

10.3.6 Reporting Exemptions

Relapse or disease progression that does not worsen in terms of time-course or severity of the disease expected in that patient is considered an efficacy outcome measure and will not be reported as an AE. Tumour-related signs and symptoms will only be recorded as AEs if they

Lee L, et al. J Immunother Cancer 2023; 11:e006699. doi: 10.1136/jitc-2023-006699

worsen in severity or increase in frequency beyond that normally expected for disease progression as determined by the Investigator.

Hospitalisation due solely to the progression of underlying malignancy will NOT be considered an SAE. Death as a result of disease progression is not an SAE.

10.4 SAFETY STOPPING CRITERIA FOR THE CLINICAL TRIAL

The study could be stopped by the SEC or IDMC upon occurrence of any of the following events:

- Unexpected and related SAE that exposed patients participating to the study to unacceptable risk of harm.
- Uncontrolled SAEs related to identified risks.
- The occurrence of Grade 4 toxicity in 3 patients, unless, in the opinion of the IDMC, is considered manageable with the institution of appropriate supportive care or dose modification.
- Death of a patient at any time after therapy that is definitely related to T cell therapy.
- The occurrence of a second malignancy at any point after therapy that is definitely related to the T cell therapy.

The study may be restarted after appropriate preventive or management guidelines have been instituted and a substantial protocol amendment has been approved by the relevant regulatory authorities and ethics committees.

10.5 SAFETY EVALUATION COMMITTEE

Patient safety will be monitored throughout all parts of the study by a SEC established by the Sponsor. This internal committee will monitor treatment-emergent data on an ongoing basis throughout study conduct for the purpose of ensuring the continued safety of patients enrolled in this study.

The SEC will be chaired by the Sponsor medical monitor and details of membership is described in the SEC charter. The team will meet at regular frequencies throughout study conduct; during dose escalation, the SEC will meet after the 1st and 3rd patient in each cohort have completed the DLT evaluation period. Additional ad hoc meetings will be instituted if safety stopping criteria is met or as clinically necessary based on merging data. Documentation of meeting outcomes will be maintained by the Sponsor. Decisions with the potential to impact patients' safety (e.g. unfavourable change in risk/benefit assessment) will be promptly communicated to Investigators, ethics committees and regulatory authorities as appropriate. Throughout the trial, information regarding all SAEs and potential DLTs will be sent to the SEC members. Dose limiting toxicities will be monitored centrally and the decision to assign the optimal dose will be taken by the SEC.

Dose escalation decisions in Phase I of the study will be made by the SEC. The schedule of dose escalation meetings will depend on the time to completion of a cohort frequency of DLT and when an RP2D is determined. The SEC may stop further enrolment into 1 or more of the cohorts if treatment-emergent toxicity is determined to result in an unfavourable change in patient risk/benefit. In Phase II, cumulative safety data will be assessed by the SEC after 10 patients are treated and on a periodic basis not exceeding 3 to 6 months. Decisions and/or

recommendations made by the SEC will be communicated to the Principal Investigators at all active study centres and to the Sponsor.

Mandatory SEC processes will be included in a SEC Charter.

10.6 INDEPENDENT DATA MONITORING COMMITTEE

An IDMC consisting of two independent physicians and one statistician will be established by the sponsor and they will review serious safety events. The IDMC will meet up on occurrence at the following, the decision of the IDMC will supersede that of the SEC:

- When any safety stopping criteria (Section 10.4) is met
- Prior to opening Phase II
- At the interim analysis of Phase II (after 10 patients have been treated)
- Annually (6 monthly during active enrolment) during Phase II to review cumulative safety data

Throughout the trial, information regarding all SAEs and DLTs as well as dose escalation meeting minutes will be sent to the IDMC members. Upon occurrence of any events as defined above, detailed event summaries and cumulative safety data will be sent to the IDMC. A decision to continue or to hold or modify the study will be made by the IDMC.

When a RP2D decision is made by the SEC, the decision will need to be reviewed and endorsed by the IDMC prior to opening Phase II.

Decisions and/or recommendations made by the IDMC will be communicated to the Principal Investigators at all active study centres and to the Sponsor.

Mandatory IDMC processes will be included in the IDMC Charter.

11 STATISTICS

Further details of the statistical analysis of all the endpoints will be included in a separate Statistical Analysis Plan. Any analysis that deviates from the Statistical Analysis Plan will be documented and justified in the Clinical Study Report.

11.1 SAMPLE SIZE ESTIMATION

Approximately 80 patients will be enrolled into the study and up to 54 patients will be treated.

- Phase I: Dose escalation: up to 24 patients in total (up to 6 per dose cohort).
- Phase II: Dose expansion: up to 30 patients in total.

In the dose escalation phase, an accelerated titration design will be used for the first cohort, with 1 patient being recruited into the lowest dose level. However, if Grade 2 or higher toxicity is observed during the DLT observation period, the design will transition into a standard 3+3 design. All subsequent cohorts will follow a 3+3 design.

Phase II of the study will follow Simon's 2-stage optimal design. The null hypothesis that the true response rate is 10% will be tested against a 1-sided alternative. In the first stage, 10 evaluable patients will be accrued (6 weeks post treatment of 10th evaluable patient). If there are 1 or fewer responses in these 10 evaluable patients, the study will be stopped. Otherwise, 19 additional evaluable patients will be accrued for a total of 29. The null hypothesis will be rejected if 6 or more responses are observed in 29 evaluable patients. This design yields a type I error rate of 5% and power of 80% when the true response rate is 30%.

11.2 DESCRIPTION OF ANALYSIS DATASETS

11.2.1 Full Analysis Set

The full analysis set will consist of all patients enrolled into the study.

11.2.2 Safety Analysis Set for the Study and for Study Treatment

All patients who have started pre-conditioning therapy of cyclophosphamide and fludarabine will be included in the safety analysis set for the study. A patient who has pre-conditioning therapy but not AUTO2 therapy will not be excluded from the safety analysis set for the study.

All patients who receive at least 1 dose (complete or partial dose) of AUTO2 therapy will be included in the safety analysis set for the Study treatment.

11.2.3 Efficacy Analysis Set

All patients who receive at least 1 dose (complete or partial dose) of AUTO2 therapy will be included in the efficacy analysis set.

11.3 STATISTICAL ANALYSES AND METHODS

Continuous data will be summarised using the mean, median, standard deviation, minimum and maximum, while frequency counts and percentages will be presented for discrete

variables. Time to event endpoints will be summarised using Kaplan-Meier method. Summary statistics will be presented for baseline characteristics.

11.3.1 Primary Endpoints

The primary endpoints of the study are as follows:

- **Phase 1: Safety (to determine the MTD and RP2D)**
  - Incidence of Grade 3 to 5 toxicity occurring within the DLT period (28 days post AUTO2 infusion).
  - Frequency of DLT and the persistence of AUTO2.
- **Phase II: Safety and Anti-tumour Effect**
  - Best overall response post-AUTO2 infusion.

11.3.1.1 Phase I: Safety

Safety associated with AUTO2 administration (only those who received AUTO2).

Summary statistics and analyses will be provided by dose level and overall. The safety analysis set for the study and study treatment will be used for the analysis of safety data.

Safety evaluations will be based on the incidence, severity and type of AEs, and changes in the patient's vital signs and clinical laboratory results.

Adverse events will be coded using the Medical Dictionary for Regulatory Activities AE coding system for purposes of summarisation. All AEs occurring on study will be listed in by-patient data listings. Treatment-emergent events will be tabulated, where treatment-emergent is defined as any AE that occurs during or after administration of AUTO2 up to 60 days after the last infusion, any event that is considered drug-related regardless of the start date of the event, or any event that is present at baseline and continues after the first dose of study treatment but worsens in intensity. Events that are considered related to treatment (possibly, probably, or definitely related) will also be tabulated. Adverse events by the NCI CTCAE toxicity grade will also be summarised. Deaths and SAEs will be tabulated in data listings including additional relevant information on the patient.

Laboratory toxicity grades will be calculated for the appropriate laboratory parameters according to NCI CTCAE Version 4.03, and the baseline and worst post-baseline values within 3 months from the AUTO2 administration observed for each patient will be summarised.

Adverse events of special interest will be analysed in greater depth, including the time to onset and time to resolution where appropriate.

11.3.1.2 Phase I: Recommended Part 2 Dose and Maximum Tolerated Dose

At the end of the Phase I dose escalation phase, the RP2D will be identified based on the safety data and evaluation of the activity data collected in the dose escalation phase.

11.3.1.3 Phase II: Anti-tumour Effect

The primary endpoints for Phase II (efficacy) will be assessed as follows:

- Best Overall response (sCR+CR+VGPR+PR) rate, following treatment with AUTO2 cells.

11.3.2 Secondary Endpoints

Feasibility of AUTO2 manufacture in this patient population (all patients)

Feasibility of product generation will be examined by assessing the number of AUTO2 successfully manufactured as a fraction of the number of patients undergoing leukapheresis (all patients registered).

Clinical efficacy of AUTO2

- Determine the clinical benefit (sCR+CR+VGPR+PR+MR) rate following treatment with AUTO2.
- Evaluate clinical outcomes including duration of response, time to disease progression, PFS, and OS.
  - Duration of response will be calculated from the date of first observation of sCR, CR, VGPR or PR to the date of disease progression, relapse or death, for patients who are considered as responders (achieved at least PR). Patients who have not progressed, relapsed or died will be censored at the last adequate disease assessment.
  - Clinical benefit (sCR+CR+VGPR+PR+MR) rate following treatment with AUTO2 cells.
  - Time to Progression will be calculated from the date of AUTO2 treatment to the date of progression. Patients who have not progressed, relapsed or died without progression/relapse will be censored at the last adequate disease assessment.
  - Progression-Free Survival will be calculated from the date of AUTO2 treatment to the date of progression or death. Patients who have not progressed or relapsed will be censored at the last adequate disease assessment.
  - Overall Survival will be calculated from the date of AUTO2 treatment to the date of death. Patients who have not died will be censored at the date of last contact (clinic visit or telephone contact).
- Time to response will be calculated from the start date of treatment to the date of first observation of sCR, CR, VGPR or PR, for patients who are considered as responders.

Biomarker and pharmacodynamic effects of AUTO2

The following analyses on the pharmacodynamic effects of AUTO2 will be performed for patients who received AUTO2:

- Expansion and persistence of RQR8/APRIL CAR positive T cells in the peripheral blood as determined by qPCR and flow cytometry will be summarised using the appropriate statistical methods:
  - Expansion is defined as the maximum level of RQR8 expression in both qPCR (copies/µg genomic deoxyribonucleic acid) and flow cytometry (cells/µL) assays during follow-up. Engraftment is defined as detection of any level of RQR8 expression in circulating T cells (i.e. PBMC) by qPCR (viral insertions per genome) and/or flow cytometry following infusion (cells/µL).
  - Persistence is defined as the duration of detectability, from infusion to the first negative result.

11.3.3 Exploratory

- Timing and magnitude of cytokine release, evaluated using cytokine bead arrays:
  - Data on timing (kinetic of change) will be summarised as the mean (or median) number of days post-infusion.
  - Magnitude – kinetic and peak of cytokine levels, e.g. TNFα, IL-6 and IFNγ (pg/mL), will be plotted for each patient and summarised.
- Depletion of BCMA+/TACI+ cell compartment, as determined by flow cytometry/or and IHC on bone marrow:
  - Percentage reduction from baseline, to nadir in absolute BCMA+ cell numbers (as measured by flow cytometry of PBMC (cells/µL) will be summarised.
- Persistence and phenotype of RQR8/APRIL CAR positive T cells in bone marrow and immune phenotype of bone marrow cells.
- Determine Minimal Residual Disease rate following AUTO2 treatment.

11.3.4 Immunogenicity Data

Analyses of immunogenicity data will be documented in the Statistical Analysis Plan.

11.3.5 Pharmacokinetics

Not applicable.

11.3.6 Other Analyses

Not applicable.

11.3.7 Interim Analysis

In the Phase II part of the study, an interim analysis will be performed after 10 patients are enrolled and considered evaluable in Phase II (6 weeks post treatment of 10th evaluable patient). The study will be stopped in this first stage if no more than 1 response (as per IMWG) has been observed. If the response rate has exceeded interim analysis stopping criteria prior to 10 evaluable patients having been enrolled, then the study will continue to full enrolment. A formal interim analysis will still be performed after the 10th evaluable patient.

12 DATA QUALITY CONTROL AND ASSURANCE

12.1 COMPLIANCE WITH THE PROTOCOL AND PROTOCOL REVISIONS

The study shall be conducted as described in the approved protocol. All revisions to the protocol must be discussed with and be prepared by the Sponsor. The Investigator should not implement any deviation or change to the protocol without prior review and documented approval/favourable opinion from the IEC/IRB of an amendment, except where necessary to eliminate an immediate hazard(s) to the study patients.

If a deviation or change to a protocol is implemented to eliminate an immediate hazard(s) prior to obtaining IEC/IRB approval/favourable opinion, as soon as possible. The deviation or change will be submitted to:

- IEC/IRB for review and approval/favourable opinion
- The Sponsor
- Regulatory authority(ies) if required by local regulations

Documentation of approval signed by the chairperson or designee of the IEC(s)/IRB(s) must be sent to the Sponsor.

If an amendment substantially alters the study design or increases the potential risk to the patient (1) the informed consent form must be revised and submitted to the IEC(s)/IRB(s) for review and approval/favourable opinion; (2) the revised form must be used to obtain consent from patients currently enrolled in the study if they are affected by the amendment; and (3) the new form must be used to obtain contact from new patients prior to enrolment.

If the revision is an administrative letter, investigators must inform their IEC(s)/IRB(s).

12.2 PROTOCOL VIOLATIONS AND DEVIATIONS

All patients who are enrolled into the study, irrespective of whether or not they receive any treatment, will be followed according to the protocol regardless of the number of treatments received, unless consent for follow-up is withdrawn. Minor protocol deviations that do not result in harm to the study patients or significantly affect the scientific value of the reported results of the study will be recorded. In case of a major protocol deviation occurring (i.e. a deviation that could have a significant effect on the patient's safety, rights, or welfare and/or on the integrity of the study data), the Investigator must notify the Sponsor and the appropriate IEC/IRB as soon as possible or as per local requirements. Major protocol deviations that meet the criteria for a serious breach of GCP (e.g. a protocol violation, or non-reporting of critical safety information potentially jeopardising patients' safety) should be reported within 24 hours to the Sponsor. The Sponsor is required to report a serious GCP breach within 7 days to the applicable Health Authorities. Protocol deviations will be recorded on the source documents with an explanation for the deviation. No deviation from the inclusion/exclusion criteria will be permitted.

12.3 MONITORING

Before the trial can be initiated at a site, the prerequisites for conducting the trial must be clarified and approved by the Sponsor.

Representatives of the Sponsor (or designee) must be allowed to visit all study site locations periodically to assess the data quality and study integrity according to FDA, EU directives and ICH GCP. On-site they will review study records and directly compare them with source documents, discuss the conduct of the study with the Investigator and verify that the facilities remain acceptable.

Electronic CRF completion and accuracy will be checked by performing source data verification that is a direct comparison of the entries made against the appropriate source documentation. Any resulting discrepancies will be reviewed with the Investigator (s) and/or his/her site staff and resolved. Monitoring procedures require that patients' informed consents, adherence to the inclusion/exclusion criteria and SAE documentation be verified. Additional monitoring activities maybe outlined in the study specific monitoring plan. The Sponsor may request adequately redacted source documents and hospital records for review by the medical monitor (e.g. pathology reports, imaging reports, cancer treatment history), this may help to better understand a patient's general condition, emerging safety issues, effectiveness of their management and the efficacy of treatment.

The Sponsor must be informed immediately of any change in the personnel involved in the conduct of the trial.

12.4 AUDITS AND SITE INSPECTIONS

Authorised personnel from domestic and foreign regulatory authorities and the Sponsor Quality Assurance (or designee) may carry out inspections and audits respectively. The purpose of an audit/inspection is to ensure that ethical, regulatory and quality requirements are fulfilled in the Sponsor studies.

The Investigator will permit international, national and local health authorities, the Sponsor monitors, representatives, and collaborators, and the IECs/IRBs to inspect facilities and records relevant to this study. The Investigator should promptly notify the Sponsor or their authorised representative of any inspections scheduled by any regulatory authorities and promptly forward to the Sponsor or their authorised representative copies of any audit reports received.

13 DATA HANDLING AND RECORD KEEPING

13.1 DATA MANAGEMENT

Data will be entered in a timely manner, for each patient into the (Sponsor approved) clinical database via electronic data capture (EDC). The EDC application uses system controls to ensure that unauthorised users cannot access or modify data. All users must have successfully undergone EDC application training prior to entering data into the EDC system. Electronic CRFs should be reviewed and electronically signed and dated by the Investigator or a designee. The eCRF system will be FDA Code of Federal Regulations 21 Part 11 compliant.

It is the responsibility of the Investigator to ensure that the data included in the eCRF is accurate, complete and electronically signed where appropriate.

The data will be electronically verified through use of on-line checks during data entry and through programmed edit checks specified by the clinical team. Discrepancies in the data will be brought to the attention of the investigational site personnel. Data entered into the eCRF will be validated as defined in the data validation specifications. All updates to queried data will be made by authorised study site personnel only and all modifications to the database will be recorded in an audit trail. Once all the queries have been resolved, eCRFs will be locked by password protection. Any changes to locked eCRFs will be approved by the Investigator. Once the full set of eCRFs have been completed and locked, the Sponsor will authorise database lock and all electronic data will be sent to the designated statistician for analysis. Subsequent changes to the database will then only be made by written agreement of the Sponsor.

Adverse events and medical/cancer history terms will be coded from the verbatim description (Investigator term) using the Medical Dictionary for Regulatory Activities. Prior and concomitant medications and therapies will be coded according to the World Health Organization drug dictionary. Coding review will be performed by the Sponsor (or designee) prior to database lock.

At the end of the study, the clinical data will be transferred to the Sponsor and the investigative site will receive patient data for their site in a readable format that must be kept with the study records.

13.2 STUDY DOCUMENTATION AND RETENTION OF RECORDS

The Investigator must maintain records of all study documents and supporting information relating to the conduct of the study. This documentation should include but is not limited to, protocols, eCRF data, SAE reports, patient source data, correspondence with health authorities and IEC/IRBs, informed consent forms, Investigator(s) and study team members' curricula vitae, monitor visit logs, laboratory reference ranges, laboratory certification or quality control procedures and laboratory director curriculum vitae (Essential documentation listed in ICH GCP (CPMP/ICH/135/95)). Patient files and other source data must be kept for the maximum period of time required by applicable regulations and guidelines or institution procedures or for the period specified by the Sponsor, whichever is longer. The Sponsor must be consulted if the Investigator(s) wishes to assign the study files to someone else, remove them to another location or is unable to retain them for the specified period.

14 CLINICAL TRIAL AGREEMENT, FINANCE AND INSURANCE

This study will be conducted under a Clinical Trial Agreement between Autolus ("Sponsor") and the institution(s) representing the investigational study site(s) ("Investigator"). Financial support to the investigational site(s) will be detailed in the Clinical Trial Agreement. The Clinical Trial Agreement must be signed before the commencement of the study and will clearly delineate the responsibilities and obligations of the Investigator and the Sponsor, and will form the contractual basis under which the clinical study will be conducted.

A Certificate of Clinical Trials Insurance will be provided to the study centres by the Sponsor, where required. Details of the Sponsor' s arrangement for clinical study insurance to provide for compensation to patients for any claim for bodily injury or death arising from participation in the clinical study are provided in the Patient Information Sheet.

15 ETHICAL AND REGULATORY CONSIDERATIONS

15.1 ETHICS COMMITTEE REVIEW AND APRROVAL

The final study protocol, including the final version of the written patient information sheet, informed consent form and any other relevant patient facing material, must be approved or given a favourable opinion in writing by an IEC/IRB as appropriate.

15.2 REGULATORY AUTHORITY REVIEW AND APPROVAL

The study will not commence before approval from the regulatory authority been granted according to local requirements. The Sponsor (or designee) will be responsible for the preparation, submission and confirmation of receipt of any regulatory authority approvals required prior to release of study treatment for shipment to the study site.

During the study, the Sponsor (or designee) is also responsible for submitting subsequent amendments and notifications to the regulatory authority according to local requirements.

15.3 INVESTIGATOR RESPONSIBILITIES

15.3.1 Overall Responsibilities

The Investigator is responsible for conducting the study in full accordance with the clinical study protocol, the latest revision of the Declaration of Helsinki, the Good Clinical Practice: Consolidated Guideline, and all applicable national and local laws and regulations for clinical research. Information regarding any investigational sites participating in this study that cannot comply with these standards will be documented and appropriate actions taken. For studies conducted in the EU/European Economic Area countries, the Investigator will ensure compliance with the EU Clinical Trial Directive [2001/20/EC]. For studies conducted in the United States or under a United States investigational new drug (IND), the Investigator will additionally ensure adherence to the basic principles of "Good Clinical Practice" as outlined in the current version of 21 Code of Federal Regulations, subchapter D, part 312, "Responsibilities of Sponsor and Investigators", part 50, "Protection of Human Subjects", and part 56, "Institutional Review Boards".

15.3.2 Site Review

Prior to the study start, the Investigator is required to sign a Protocol signature page confirming his/her agreement to conduct the study in accordance with these documents and all of the instructions and procedures found in this Protocol, and to give access to all relevant data and records to Clinical Research Associates, auditors and regulatory authorities as required. Investigators ascertain they will apply due diligence to avoid protocol deviations.

The Investigator will make appropriate reports on the progress of this study to the Sponsor or its designee in accordance with applicable government regulations and their agreement with the Sponsor/Contract Research Organisation.

15.3.3 Informed Consent

It is the responsibility of the Investigator to obtain written informed consent from patients prior to conducting any study-related procedures. All consent documentation must be in accordance with applicable regulations and ICH GCP. Each patient is requested to sign and

date the informed consent form after she/he has received and read the patient information sheet and received an explanation of what the study involves, including but not limited to: the objectives, potential benefits and risk, inconveniences and the patient's rights and responsibilities. Patients will be given adequate time to evaluate the information given to them before signing and dating the informed consent form. It will also be explained to the patients that they are free to refuse entry into the study at any time and without prejudice to future treatment. The informed consent form must also be signed and dated by the person obtaining consent. The original signed informed consent form for each patient will be retained on file by the Investigator and the second signed original given to the patient.

Informed consent forms must be retained for all screened patients and must be available for verification by the Study Monitor at any time.

In the event of changes to the informed consent form during the study, the Investigator must always use the most current IEC/IRB approved form for documenting written informed consent.

15.3.4 Delegation of Investigator Duties

The Investigator should ensure that all persons involved in the clinical study are adequately qualified, informed about the protocol, any amendment to the protocol, the study treatments, and their study related duties and functions before any involvement takes place. Delegation of any study related duties and documentation of training performed will be recorded in the signature and delegation log.

15.3.5 Communication with Independent Ethics Committee (IEC)/Institutional Review Board (IRB)

A list of IEC/IRB members should be obtained by the Investigator or qualified designee and provided to the Sponsor/ representative.

The Sponsor or its designee is responsible to assist the Investigator with applicable documentation for communication with IECs/IRBs. Before initiating a trial, the Investigator/institution should have written and dated approval of the study protocol, the patient informed consent form, any written information to the patients, patients' recruitments procedures (e.g. advertisement if applicable), Investigator's brochure, Investigational Medicinal Product labelling (if applicable) and the coordinating investigator's curriculum vitae to the relevant IEC/IRB for evaluation before the study start. The IEC/IRB's unconditional approval statement will be transmitted by the Investigator to the Sponsor or a designee prior to shipment of the ATIMP to the site. This approval must refer to the study by exact protocol title and number, and should identify the documents reviewed and the date of review.

During the trial, the Investigator or designee is responsible for forwarding the applicable documents for approval and/or review including protocol deviations, protocol amendments, informed consent form changes or revisions of other documents originally submitted for review; serious and/or unexpected adverse experiences occurring during the study; new information that may affect adversely the safety of the patients or the conduct of the study; an annual update and/or request for re-approval; and when the study has been completed. The Investigator or designee will follow national and local requirements.

The Investigator will submit the informed consent to the Sponsor for approval prior to submitting to the IEC/IRB.

The Investigator must supply the Sponsor with a copy of the list of members of the IEC/IRB and the letter(s) of approval(s) defining the version of each document approved.

15.3.6 Confidentiality of Trial Documents and Patient Records

The Investigator must ensure that patients' anonymity will be maintained and that their identities are protected from unauthorised parties. The Sponsor will maintain confidentiality standards by assigning a unique coded identification number to each patient included in the study. Patient names will never be included in data sets that are transmitted to the Sponsor or their representatives, or to third parties as permitted by the Informed Consent Form.

On eCRFs or other documents submitted to the Sponsor, patients will not be identified by their names, but by an identification code. The Investigator will keep a patient enrolment log relating codes to the names of patients. The Investigator will maintain documents not for submission to the Sponsor, e.g. patients' written consent forms, in strict confidence. Records will not be destroyed without giving the Sponsor prior written notice and the opportunity to further store such records, at the Sponsor's cost and expense.

After obtaining a patient's consent, the Investigator will permit the study monitor, independent auditor or regulatory agency personnel to review the portion of the patient's medical record directly related to the study. This shall include all study relevant documentation (e.g. patient medical history to verify eligibility, laboratory tests result, admission/discharge summaries for hospital admissions occurring while the patient is on study, and autopsy reports for deaths occurring during the study).

Patient medical information obtained by this study is confidential and may only be disclosed to third parties as permitted by the Informed Consent Form signed by the patient, unless permitted or required by law.

15.4 LONG-TERM RETENTION OF SAMPLES FOR ADDITIONAL FUTURE RESEARCH

Samples collected in this study may be stored for up to 15 years (or according to local regulations) for additional research. Samples will only be used to further understand AUTO2, to understand advanced cancers, to understand differential drug responders, and to develop tests/assays related to AUTO2 and advanced cancer. The research may begin at any time during the study or the post-study storage period.

Stored samples will be coded throughout the sample storage and analysis process and will not be labelled with personal identifiers. Patients may withdraw their consent for their samples to be stored for research.

16 STUDY COMPLETION

All materials or supplies provided by the Sponsor will be returned to the Sponsor upon study completion. The study will be considered complete when the last patient completes their final follow-up visit and all the data clarification forms have been resolved. A final Clinical Study Report will be provided to the relevant authorities within 12 months after the completion of the study.

The Sponsor reserves the right to terminate this study at any time for reasonable medical or administrative reasons. Any premature discontinuation will be appropriately documented according to local requirements (e.g. IEC/IRB, regulatory authorities). In addition, the Investigator or the Sponsor has the right to discontinue a single site at any time during the study for medical or administrative reasons such as:

- Significant non-compliance with contractual enrolment timelines and targets
- Serious or continued GCP non-compliance
- Inaccurate, incomplete or delayed data collection
- Failure to adhere to the study protocol
- Failure to provide requested follow-up information for data queries.

17 PUBLICATION OF DATA AND PROTECTION OF TRADE SECRETS

Section 801 of the FDA Amendments Act mandates the registration with ClinicalTrials.gov of certain clinical trials of drugs (including biological products) and medical devices subject to FDA regulations for any disease or condition. The International Committee of Medical Journal Editors requires study registration as a condition for publication of research results generated by a clinical study (http://www.icmje.org).

All information supplied by the Sponsor in connection with this study and not previously published to the public, is considered confidential information ("Confidential Information"). This confidential information includes, but is not limited to, the Investigators' Brochure, clinical protocol, CRFs and other scientific data. Any data collected during the study are also considered confidential information. This confidential information shall remain the sole and exclusive property of the Sponsor, shall not be disclosed to others without prior written consent of the Sponsor, and shall not be used except in the performance of this study.

The information developed during the conduct of this study is also considered confidential information, and will be used by the Sponsor in connection with the development of the ATIMP. The confidential information may be disclosed as deemed necessary by the Sponsor. To allow the use of the confidential information derived from this study, the Investigator is obliged to provide the Sponsor with complete test results and all data developed in this study.

The Sponsor has full ownership of the original CRFs completed as part of the study.

By signing the clinical study protocol, the Investigator agrees that the results of the study may be used for the purposes of national and international registration, publication, and information for medical and pharmaceutical professionals. The authorities will be notified of the Investigator's name, address, qualifications, and extent of involvement.

It is agreed that, consistent with scientific standards, publication of the results of the study shall be made only as part of a publication of the results obtained by all sites performing the protocol.

Should the Investigator wish to publish the results of this study, the Investigator agrees to provide the Sponsor with a manuscript for review 60 days prior to submission for publication.

The Sponsor retains the right to delete from the manuscript, information that is confidential or proprietary and to object suggested publication and/or its timing (at the Sponsor's discretion).

In addition, if requested by the Sponsor, the Investigator shall withhold publication an additional 6 months to allow for filing a patent application or taking such other measures as the Sponsor deems appropriate to establish and preserve its proprietary rights.

In the event that the Sponsor chooses to publish the data from this study, a copy will be provided to the Investigator at least 30 days prior to the expected date of submission to the intended publisher.

18 REFERENCES

Ali, SA, Shi V, Maric I, Wang M, Stroncek DF, Rose JJ, et al. (2016). T cells expressing an anti-B-cell maturation antigen chimeric antigen receptor cause remissions of multiple myeloma. Blood **128**(13): 1688-1700.

Attal, M, Harousseau JL, Stoppa AM, Sotto JJ, Fuzibet JG, Rossi JF, et al. (1996). A prospective, randomized trial of autologous bone marrow transplantation and chemotherapy in multiple myeloma. Intergroupe Francais du Myelome. N Engl J Med **335**(2): 91-97.

Berdeja, JG, Bauer T, Arrowsmith E, Essell J, Murphy P, Reeves JA, Jr., et al. (2017). Phase II study of bendamustine, bortezomib and dexamethasone (BBD) in the first-line treatment of patients with multiple myeloma who are not candidates for high dose chemotherapy. Br J Haematol **177**(2): 254-262.

Brentjens, RJ, Latouche JB, Santos E, Marti F, Gong MC, Lyddane C, et al. (2003). Eradication of systemic B-cell tumors by genetically targeted human T lymphocytes co-stimulated by CD80 and interleukin-15. Nat Med **9**(3): 279-286.

Brentjens, RJ, Rivière I, Park JH, Davila ML, Wang X, Stefanski J, et al. (2011). Safety and persistence of adoptively transferred autologous CD19-targeted T cells in patients with relapsed or chemotherapy refractory B-cell leukemias. Blood **118**(18): 4817-4828.

Brudno, JN and Kochenderfer JN (2016). Toxicities of chimeric antigen receptor T cells: recognition and management. Blood **127**(26): 3321-3330.

Carpenter, RO, Evbuomwan MO, Pittaluga S, Rose JJ, Raffeld M, Yang S, et al. (2013). B-cell maturation antigen is a promising target for adoptive T-cell therapy of multiple myeloma. Clinical Cancer Research: An Official Journal of the American Association for Cancer Research **19**(8): 2048-2060.

Child, JA, Morgan GJ, Davies FE, Owen RG, Bell SE, Hawkins K, et al. (2003). High-dose chemotherapy with hematopoietic stem-cell rescue for multiple myeloma. N Engl J Med **348**(19): 1875-1883.

Chu, CS, Boyer J, Schullery DS, Gimotty PA, Gamerman V, Bender J, et al. (2012). Phase I/II randomized trial of dendritic cell vaccination with or without cyclophosphamide for consolidation therapy of advanced ovarian cancer in first or second remission. Cancer Immunol Immunother **61**(5): 629-641.

Claudio, JO, Masih-Khan E, Tang H, Goncalves J, Voralia M, Li ZH, et al. (2002). A molecular compendium of genes expressed in multiple myeloma. Blood **100**(6): 2175-2186.

Cooper, LJN, Topp MS, Serrano LM, Gonzalez S, Chang W-C, Naranjo A, et al. (2003). T-cell clones can be rendered specific for CD19: toward the selective augmentation of the graft-versus-B-lineage leukemia effect. Blood **101**(4): 1637-1644.

CRUK (2014). Myeloma Statistics. Retrieved June 2016 from http://www.cancerresearchuk.org/health-professional/cancer-statistics/statistics-by-cancer-type/myeloma.

Curti, BD, Ochoa AC, Powers GC, Kopp WC, Alvord WG, Janik JE, et al. (1998). Phase I trial of anti-CD3-stimulated CD4+ T cells, infusional interleukin-2, and cyclophosphamide in patients with advanced cancer. J Clin Oncol **16**(8): 2752-2760.

Dudley, ME, Yang JC, Sherry R, Hughes MS, Royal R, Kammula U, et al. (2008). Adoptive cell therapy for patients with metastatic melanoma: evaluation of intensive myeloablative chemoradiation preparative regimens. Journal of Clinical Oncology: Official Journal of the American Society of Clinical Oncology **26**(32): 5233-5239.

Durie, BGM (2006). International uniform response criteria for multiple myeloma. Leukemia: official journal of the Leukemia Society of America, Leukemia Research Fund, U.K **20**(10): 1-7.

Enblad, G, Karlsson H, Wikstrom K, Essand M, Savoldo B, Brenner MK, et al. (2015). Third Generation CD19-CAR T Cells for Relapsed and Refractory Lymphoma and Leukemia Report from the Swedish Phase I/IIa Trial. Blood **126**(23): 1534.

Fan, F, Zhao W, Liu J, He A, Chen Y, Cao X, et al. (2017). Durable remissions with BCMA-specific chimeric antigen receptor (CAR)-modified T cells in patients with refractory/relapsed multiple myeloma. Journal of Clinical Oncology **35**(18_suppl): LBA3001-LBA3001.

FDA (2017). YESCARTA Prescribing Information. Retrieved 19 December 2017 from https://www.fda.gov/downloads/BiologicsBloodVaccines/CellularGeneTherapyProducts/ApprovedProducts/UCM581226.pdf.

FDA (Undated). KYMRIAH Prescribing Information. Retrieved 19 December 2017 from https://www.fda.gov/downloads/BiologicsBloodVaccines/CellularGeneTherapyProducts/ApprovedProducts/UCM573941.pdf.

Frisen, L (1982). Swelling of the optic nerve head: a staging scheme. J Neurol Neurosurg Psychiatry **45**(1): 13-18.

Fry, EE (2016). CD22 CAR Update and Novel Mechanisms of Leukemic Resistance. American Association for Cancer Research, New Orleans, Louisiana.

Fry, TJ, Stetler-Stevenson M, Shah NN, Yuan CM, Yates B, Delbrook C, et al. (2015). Clinical Activity and Persistence of Anti-CD22 Chimeric Antigen Receptor in Children and Young Adults with Relapsed/Refractory Acute Lymphoblastic Leukemia (ALL). Blood **126**(23): 1324-1324.

Garcia-Carmona, Y, Cols M, Ting AT, Radigan L, Yuk FJ, Zhang L, et al. (2015). Differential induction of plasma cells by isoforms of human TACI. Blood **125**(11): 1749-1758.

Gardner, R, Leger KJ, Annesley CE, Summers C, Rivers J, Gust J, et al. (2016). Decreased Rates of Severe CRS Seen with Early Intervention Strategies for CD19 CAR-T Cell Toxicity Management. Blood **128**(22): 586-586.

Garfall, AL, Maus MV, Hwang WT, Lacey SF, Mahnke YD, Melenhorst JJ, et al. (2015). Chimeric Antigen Receptor T Cells against CD19 for Multiple Myeloma. The New England Journal of Medicine **373**(11): 1040-1047.

Gargett, T, Fraser CK, Dotti G, Yvon ES and Brown MP (2015). BRAF and MEK inhibition variably affect GD2-specific chimeric antigen receptor (CAR) T-cell function in vitro. Journal of Immunotherapy (Hagerstown, Md.: 1997) **38**(1): 12-23.

Geller, MA, Cooley S, Judson PL, Ghebre R, Carson LF, Argenta PA, et al. (2011). A phase II study of allogeneic natural killer cell therapy to treat patients with recurrent ovarian and breast cancer. Cytotherapy **13**(1): 98-107.

Goebeler, M-E, Knop S, Viardot A, Kufer P, Topp MS, Einsele H, et al. (2016). Bispecific T-Cell Engager (BiTE) Antibody Construct Blinatumomab for the Treatment of Patients With Relapsed/Refractory Non-Hodgkin Lymphoma: Final Results From a Phase I Study. Journal of Clinical Oncology **34**(10): 1104-1111.

Hallek, M (2013). Chronic lymphocytic leukemia: 2013 update on diagnosis, risk stratification and treatment. Am J Hematol **88**(9): 803-816.

Harousseau, JL and Moreau P (2009). Autologous hematopoietic stem-cell transplantation for multiple myeloma. N Engl J Med **360**(25): 2645-2654.

Helton, KJ, Patay Z and Triplett BM (2013). Fludarabine-induced severe necrotizing leukoencephalopathy in pediatric hematopoietic cell transplantation. Bone Marrow Transplantation **48**(5): 729-731.

Hoyos, V, Savoldo B, Quintarelli C, Mahendravada A, Zhang M, Vera J, et al. (2010). Engineering CD19-specific T lymphocytes with interleukin-15 and a suicide gene to enhance their anti-lymphoma/leukemia effects and safety. Leukemia **24**(6): 1160-1170.

Jakubowiak, A, Offidani M, Pegourie B, De La Rubia J, Garderet L, Laribi K, et al. (2016). Randomized phase 2 study: elotuzumab plus bortezomib/dexamethasone vs bortezomib/dexamethasone for relapsed/refractory MM. Blood **127**(23): 2833-2840.

Jensen, MC, Popplewell L, Cooper LJ, DiGiusto D, Kalos M, Ostberg JR, et al. (2010). Antitransgene rejection responses contribute to attenuated persistence of adoptively transferred CD20/CD19-specific chimeric antigen receptor redirected T cells in humans. Biol Blood Marrow Transplant **16**(9): 1245-1256.

Kenderian, SS, Porter DL and Gill S (2017). Chimeric Antigen Receptor T Cells and Hematopoietic Cell Transplantation: How Not to Put the CART Before the Horse. Biol Blood Marrow Transplant **23**(2): 235-246.

Kershaw, MH, Teng MWL, Smyth MJ and Darcy PK (2005). Supernatural T cells: genetic modification of T cells for cancer therapy. Nature Reviews. Immunology **5**(12): 928-940.

Kimberley, FC, van der Sloot AM, Guadagnoli M, Cameron K, Schneider P, Marquart JA, et al. (2012). The design and characterization of receptor-selective APRIL variants. The Journal of Biological Chemistry **287**(44): 37434-37446.

Klebanoff, CA, Khong HT, Antony PA, Palmer DC and Restifo NP (2005). Sinks, suppressors and antigen presenters: how lymphodepletion enhances T cell-mediated tumor immunotherapy. Trends in Immunology **26**(2): 111-117.

Klinger, M, Benjamin J, Kischel R, Stienen S and Zugmaier G (2016). Harnessing T cells to fight cancer with BiTE® antibody constructs – past developments and future directions. Immunological Reviews **270**(1): 193-208.

Kochenderfer, J, Somerville R, Lu T, Shi V, Yang JC, Sherry R, et al. (2016). Anti-CD19 chimeric antigen receptor T cells preceded by low-dose chemotherapy to induce remissions of advanced lymphoma. Journal of Clinical Oncology **34**(15_suppl): LBA3010-LBA3010.

Kochenderfer, JN, Dudley ME, Feldman SA, Wilson WH, Spaner DE, Maric I, et al. (2012). B-cell depletion and remissions of malignancy along with cytokine-associated toxicity in a clinical trial of anti-CD19 chimeric-antigen-receptor-transduced T cells. Blood **119**(12): 2709-2720.

Kochenderfer, JN, Dudley ME, Kassim SH, Somerville RP, Carpenter RO, Stetler-Stevenson M, et al. (2015). Chemotherapy-refractory diffuse large B-cell lymphoma and indolent B-cell malignancies can be effectively treated with autologous T cells expressing an anti-CD19 chimeric antigen receptor. J Clin Oncol **33**(6): 540-549.

Kochenderfer, JN, Yu Z, Frasheri D, Restifo NP and Rosenberg SA (2010). Adoptive transfer of syngeneic T cells transduced with a chimeric antigen receptor that recognizes murine CD19 can eradicate lymphoma and normal B cells. Blood **116**(19): 3875-3886.

Kocoglu, M and Badros A (2016). The Role of Immunotherapy in Multiple Myeloma. Pharmaceuticals **9**(1).

Kumar, S, Lee JH, Lahuerta JJ, Morgan G, Richardson PG, Crowley J, et al. (2012). Risk of Progression and Survival in Multiple Myeloma Relapsing After Therapy with IMiDs and Bortezomib: A Multicenter International Myeloma Working Group Study. Leukemia **26**(1): 149-157.

Kumar, S, Paiva B, Anderson KC, Durie B, Landgren O, Moreau P, et al. (2016). International Myeloma Working Group consensus criteria for response and minimal residual disease assessment in multiple myeloma. Lancet Oncol **17**(8): e328-e346.

Kyle, RA, Gertz MA, Witzig TE, Lust JA, Lacy MQ, Dispenzieri A, et al. (2003). Review of 1027 patients with newly diagnosed multiple myeloma. Mayo Clin Proc **78**(1): 21-33.

Kyle, RA and Rajkumar SV (2004). Multiple myeloma. N Engl J Med **351**(18): 1860-1873.

Kyle, RA and Rajkumar SV (2009). Criteria for diagnosis, staging, risk stratification and response assessment of multiple myeloma. Leukemia **23**(1): 3-9.

Kyle, RA, Yee GC, Somerfield MR, Flynn PJ, Halabi S, Jagannath S, et al. (2007). American Society of Clinical Oncology 2007 clinical practice guideline update on the role of bisphosphonates in multiple myeloma. J Clin Oncol **25**(17): 2464-2472.

Laubach, JP, Mitsiades CS, Mahindra A, Schlossman RL, Hideshima T, Chauhan D, et al. (2009). Novel therapies in the treatment of multiple myeloma. J Natl Compr Canc Netw **7**(9): 947-960.

Laubach, JP, Voorhees PM, Hassoun H, Jakubowiak A, Lonial S and Richardson PG (2014). Current strategies for treatment of relapsed/refractory multiple myeloma. Expert review of hematology **7**(1): 97-111.

Laurent, J, Speiser DE, Appay V, Touvrey C, Vicari M, Papaioannou A, et al. (2010). Impact of 3 different short-term chemotherapy regimens on lymphocyte-depletion and reconstitution in melanoma patients. Journal of Immunotherapy (Hagerstown, Md.: 1997) **33**(7): 723-734.

Lee, DW, Gardner R, Porter DL, Louis CU, Ahmed N, Jensen M, et al. (2014). Current concepts in the diagnosis and management of cytokine release syndrome. Blood **124**(2): 188-195.

Lee, DW, Kochenderfer JN, Stetler-Stevenson M, Cui YK, Delbrook C, Feldman SA, et al. (2015). T cells expressing CD19 chimeric antigen receptors for acute lymphoblastic leukaemia in children and young adults: a phase 1 dose-escalation trial. Lancet **385**(9967): 517-528.

Lee, L, Bounds D, Paterson J, Herledan G, Sully K, Seestaller-Wehr LM, et al. (2016). Evaluation of B cell maturation antigen as a target for antibody drug conjugate mediated cytotoxicity in multiple myeloma. British Journal of Haematology: n/a-n/a.

Lee L, *et al. J Immunother Cancer* 2023; 11:e006699. doi: 10.1136/jitc-2023-006699

Lonial, S, Weiss BM, Usmani SZ, Singhal S, Chari A, Bahlis NJ, et al. (2016). Daratumumab monotherapy in patients with treatment-refractory multiple myeloma (SIRIUS): an open-label, randomised, phase 2 trial. Lancet (London, England) **387**(10027): 1551-1560.

Louis, CU, Savoldo B, Dotti G, Pule M, Yvon E, Myers GD, et al. (2011). Antitumor activity and long-term fate of chimeric antigen receptor-positive T cells in patients with neuroblastoma. Blood **118**(23): 6050-6056.

Moreaux, J, Cremer FW, Reme T, Raab M, Mahtouk K, Kaukel P, et al. (2005). The level of TACI gene expression in myeloma cells is associated with a signature of microenvironment dependence versus a plasmablastic signature. Blood **106**(3): 1021-1030.

Muranski, P, Boni A, Wrzesinski C, Citrin DE, Rosenberg SA, Childs R, et al. (2006). Increased intensity lymphodepletion and adoptive immunotherapy--how far can we go? Nature Clinical Practice. Oncology **3**(12): 668-681.

NCT02064387 (2014). Dose Escalation Study to Investigate the Safety, Pharmacokinetics, Pharmacodynamics, Immunogenicity and Clinical Activity of GSK2857916. Retrieved 15 August 2017 from https://clinicaltrials.gov/ct2/show/NCT02064387?term=NCT02064387&rank=1.

NCT02215967 (2014). Study of T Cells Targeting B-Cell Maturation Antigen for Previously Treated Multiple Myeloma. from https://clinicaltrials.gov/ct2/show/NCT02215967?term=NCT02215967&rank=1.

NCT02348216 (2016). A Phase 1-2 Multi-Center Study Evaluating KTE-C19 in Subjects With Refractory Aggressive Non-Hodgkin Lymphoma (ZUMA-1) - Full Text View - ClinicalTrials.gov. Retrieved 11 April 2017 from https://clinicaltrials.gov/ct2/show/NCT02348216.

NCT02535364 (2016). Study Evaluating the Efficacy and Safety of JCAR015 in Adult B-cell Acute Lymphoblastic Leukemia (B-ALL) - Full Text View - ClinicalTrials.gov. Retrieved 11 April 2017 from https://clinicaltrials.gov/ct2/show/NCT02535364.

NCT02546167 (2015). CART-BCMA Cells for Multiple Myeloma. Retrieved 15 August 2017 from https://clinicaltrials.gov/ct2/show/NCT02546167?term=NCT02546167&rank=1.

NCT02658929 (2016). Study of bb2121 in Multiple Myeloma. Retrieved 15 August 2017 from https://clinicaltrials.gov/ct2/show/NCT02658929?term=NCT02658929&rank=1.

Neelapu, SS, Locke FL, Bartlett NL, Siddiqi T, Chavez JC, Hosing C, et al. (2016). Ongoing complete remissions (CR) in the phase 1 of ZUMA-1: a phase 1-2 multicenter study evaluating the safety and efficacy of KTE-C19 (anti-CD19 CAR T cells) in subjects with refractory aggressive B-cell Non-Hodgkin Lymphoma (NHL). Journal of Clinical Oncology **34**(15_suppl): 7559-7559.

Neelapu, SS, Tummala S, Kebriaei P, Wierda W, Gutierrez C, Locke FL, et al. (2017). Chimeric antigen receptor T-cell therapy - assessment and management of toxicities. Nat Rev Clin Oncol.

Oken, MM, Creech RH, Tormey DC, Horton J, Davis TE, McFadden ET, et al. (1982). Toxicity and response criteria of the Eastern Cooperative Oncology Group. Am J Clin Oncol **5**(6): 649-655.

Palumbo, A and Anderson K (2011). Multiple myeloma. The New England Journal of Medicine **364**(11): 1046-1060.

Philip, B, Kokalaki E, Mekkaoui L, Thomas S, Straathof K, Flutter B, et al. (2014). A highly compact epitope-based marker/suicide gene for easier and safer T-cell therapy. Blood **124**(8): 1277-1287.

Porter, DL, Levine BL, Kalos M, Bagg A and June CH (2011). Chimeric antigen receptor-modified T cells in chronic lymphoid leukemia. The New England Journal of Medicine **365**(8): 725-733.

Rajkumar, SV, Harousseau JL, Durie B, Anderson KC, Dimopoulos M, Kyle R, et al. (2011). Consensus recommendations for the uniform reporting of clinical trials: report of the International Myeloma Workshop Consensus Panel 1. Blood **117**(18): 4691-4695.

Rossi, JM, Neelapu SS, Go WY, Shen Y-w, Sherman M, Locke FL, et al. (2015). Phase 1 Biomarker Analysis of the ZUMA-1 (KTE-C19-101) Study: A Phase 1-2 Multi-Center Study Evaluating the Safety and Efficacy of Anti-CD19 CAR T Cells (KTE-C19) in Subjects with Refractory Aggressive Non-Hodgkin Lymphoma (NHL). Blood **126**(23): 2730-2730.

Ruella, M, Kenderian SS, Shestova O, Klichinsky M, Melenhorst JJ, Wasik MA, et al. (2017). Kinase inhibitor ibrutinib to prevent cytokine-release syndrome after anti-CD19 chimeric antigen receptor T cells for B-cell neoplasms. Leukemia **31**(1): 246-248.

San-Miguel, JF, Hungria VT, Yoon SS, Beksac M, Dimopoulos MA, Elghandour A, et al. (2014). Panobinostat plus bortezomib and dexamethasone versus placebo plus bortezomib and dexamethasone in patients with relapsed or relapsed and refractory multiple myeloma: a multicentre, randomised, double-blind phase 3 trial. Lancet Oncol **15**(11): 1195-1206.

Savoldo, B, Ramos CA, Liu E, Mims MP, Keating MJ, Carrum G, et al. (2011). CD28 costimulation improves expansion and persistence of chimeric antigen receptor-modified T cells in lymphoma patients. J Clin Invest **121**(5): 1822-1826.

Simon, R, Freidlin B, Rubinstein L, Arbuck SG, Collins J and Christian MC (1997). Accelerated titration designs for phase I clinical trials in oncology. J Natl Cancer Inst **89**(15): 1138-1147.

Sotillo, E, Barrett DM, Black KL, Bagashev A, Oldridge D, Wu G, et al. (2015). Convergence of Acquired Mutations and Alternative Splicing of CD19 Enables Resistance to CART-19 Immunotherapy. Cancer Discov **5**(12): 1282-1295.

Spear, P, Barber A and Sentman CL (2013). Collaboration of chimeric antigen receptor (CAR)-expressing T cells and host T cells for optimal elimination of established ovarian tumors. Oncoimmunology **2**(4): e23564.

Sporn, JR, Ergin MT, Robbins GR, Cable RG, Silver H and Mukherji B (1993). Adoptive immunotherapy with peripheral blood lymphocytes cocultured in vitro with autologous tumor cells and interleukin-2. Cancer Immunol Immunother **37**(3): 175-180.

Tai, Y-T and Anderson KC (2015). Targeting B-cell maturation antigen in multiple myeloma. Immunotherapy.

Tarte, K, De Vos J, Thykjaer T, Zhan F, Fiol G, Costes V, et al. (2002). Generation of polyclonal plasmablasts from peripheral blood B cells: a normal counterpart of malignant plasmablasts. Blood **100**(4): 1113-1122.

Tarte, K, Zhan F, De Vos J, Klein B and Shaughnessy J, Jr. (2003). Gene expression profiling of plasma cells and plasmablasts: toward a better understanding of the late stages of B-cell differentiation. Blood **102**(2): 592-600.

Turtle, CJ, Hanafi LA, Berger C, Gooley TA, Cherian S, Hudecek M, et al. (2016). CD19 CAR-T cells of defined CD4+:CD8+ composition in adult B cell ALL patients. J Clin Invest **126**(6): 2123-2138.

Wrzesinski, C and Restifo NP (2005). Less is more: lymphodepletion followed by hematopoietic stem cell transplant augments adoptive T-cell-based anti-tumor immunotherapy. Current Opinion in Immunology **17**(2): 195-201.

Xu, Y, Zhang M, Ramos CA, Durett A, Liu E, Dakhova O, et al. (2014). Closely related T-memory stem cells correlate with in vivo expansion of CAR.CD19-T cells and are preserved by IL-7 and IL-15. Blood **123**(24): 3750-3759.

19 APPENDICES

Appendix 1: International Myeloma Working Group (IMWG) response criteria for multiple myeloma for documenting disease response

Response Category*	IMWG Criteria
Stringent clinical response	Complete response as defined below plus normal free light chain (FLC) ratio and absence of clonal cells in bone marrow by immunohistochemistry or immunofluorescence [a].
Complete response (CR)	Negative immunofixation on the serum and urine and disappearance of any soft tissue plasmacytomas[b] and <5% plasma cells in bone marrow [c].
Very good partial response (VGPR)	Serum and urine monoclonal protein (M-protein) detectable by immunofixation but not on electrophoresis or ≥90% reduction in serum M-protein plus urine M-protein level <100 mg/24 hours.
Partial response	≥50% reduction of serum M-protein and reduction in 24 hours urinary M-protein by ≥90% or to <200 mg/24 hours.<br>If the serum and urine M-protein are unmeasurable a ≥50% decrease in the difference between involved and uninvolved FLC levels is required in place of the M-protein criteria.<br>If serum and urine M-protein are not measurable, and serum free light assay is also not measurable, ≥50% reduction in plasma cells is required in place of M-protein, provided baseline bone marrow plasma cell percentage was ≥30%.<br>In addition to the above listed criteria, if present at baseline, a ≥50% reduction in the size of soft tissue plasmacytomas (SPD) is also required.[b]
Minimal Response	≥25% but ≤49% reduction of serum M-protein and reduction in 24-hour urine M-protein by 50 to 89%.<br>If present, a 25% to 49% reduction in the size of soft tissue plasmacytomas is also required.[b]
No change/Stable disease	Not meeting criteria for CR, VGPR, partial response, minimal response or progressive disease.
Progressive disease[d,e]	Increase of ≥25% from lowest response value in any 1 or more of the following:<br>Serum M-component and/or (the absolute increase must be ≥0.5 g/dL)<br>Serum M-component increases of ≥1 g/dL are sufficient to define relapse if starting M-component is ≥5 g/dL<br>Urine M-component and/or (the absolute increase must be ≥200 mg/24 hours)<br>Only in patients without measurable serum and urine M-protein levels; the difference between involved and uninvolved FLC levels. The absolute increase must be >10 mg/dL.<br>Bone marrow plasma cell percentage; the absolute percentage must be ≥10%[f]<br>Appearance of a new lesion(s), ≥50% increase from nadir in SPD of >1 lesion, or ≥50% increase in the longest diameter of a previous lesion >1 cm in short axis[b]<br>≥50% increase in circulating plasma cells (minimum of 200 cells per µL) if this is the only measure of disease.
Clinical Relapse	Clinical relapse requires 1 or more of:<br>Direct indicators of increasing disease and/or end organ dysfunction (Calcium elevation, Renal insufficiency, Anaemia and Bone abnormalities). It is not used in calculation of time to progression or progression-free survival] but is listed here as something that can be reported optionally or for use in clinical practice.<br>Development of new soft tissue plasmacytomas or bone lesions (osteoporotic fractures do not constitute progression).

**Response Category***	**IMWG Criteria**
	Definite increase in the size of existing plasmacytomas or bone lesions. A definite increase is defined as a 50% (and at least 1 cm) increase as measured serially by the SPD of the measurable lesion.<br>Hypercalcaemia (>11 mg/dL);<br>Decrease in haemoglobin of ≥2 g/dL not related to therapy or other non-myeloma-related conditions;<br>Rise in serum creatinine by 2 mg/dL or more from the start of the therapy and attributable to myeloma;<br>Hyperviscosity related to serum paraprotein
Relapse from CR [d] (To be used only if the end point studied is disease free survival) [g]	Any 1 or more of the following:<br>Reappearance of serum or urine M-protein by immunofixation or electrophoresis.<br>Development of ≥5% plasma cells in the bone marrow [f]<br>Appearance of any other sign of progression (i.e. new plasmacytoma, lytic bone lesion, or hypercalcaemia).

CR=complete response; FLC=free light chain; M-protein=monoclonal protein; PR=partial response; SPD=sum of the products of the maximal perpendicular diameters; VGPR=very good partial response.

*All response categories require two consecutive assessments made any time before starting any new therapy

Note: A clarification to IMWG criteria for coding CR and VGPR in patients in whom the only measurable disease is by serum FLC levels: CR in such patients is defined as a normal FLC ratio of 0.26 to 1.65 in addition to CR criteria listed above. Very good partial response in such patients is defined as a >90% decrease in the difference between involved and uninvolved FLC levels.

[a] Presence/absence of clonal cells is based upon the kappa/lambda ratio. An abnormal kappa/lambda ratio by immunohistochemistry and/or immunofluorescence requires a minimum of 100 plasma cells for analysis. An abnormal ratio reflecting presence of an abnormal clone is kappa/lambda of >4:1 or <1:2.

[b] Plasmacytoma measurements should be taken from the CT portion of the PET/CT, or MRI scans, or dedicated CT scans where applicable. For patients with only skin involvement, skin lesions should be measured with a ruler

[c] Confirmation with repeat bone marrow biopsy not needed.

[d] All relapse categories require 2 consecutive assessments made at any time before classification as relapse or disease progression and/or the institution of any new therapy. In the IMWG criteria, CR patients must also meet the criteria for progressive disease shown here to be classified as progressive disease for the purposes of calculating time to progression and progression-free survival. The definitions of clinical relapse, relapse from CR and relapse from MRD are not to be used in calculation of time to progression or progression-free survival.

[e] In the case where a value is felt to be a spurious result per physician discretion (e.g., a possible laboratory error), that value will not be considered when determining the lowest value.

[f] Relapse from CR has the 5% cut-off versus 10% for other categories of relapse.

[g] For purposes of calculating time to progression and progression-free survival, CR patients should also be evaluated using criteria listed above for progressive disease.

Appendix 2: Eastern Cooperative Oncology Group Performance Status Score

**Grade**	**Eastern Cooperative Oncology Group Performance Status**
0	Fully active, able to carry on all pre-disease performance without restriction
1	Restricted in physically strenuous activity but ambulatory and able to carry out work of a light or sedentary nature, e.g. light house work, office work
2	Ambulatory and capable of all self-care but unable to carry out any work activities. Up and about more than 50% of waking hours
3	Capable of only limited self-care, confined to bed or chair more than 50% of waking hours
4	Completely disabled. Cannot carry on any self-care. Totally confined to bed or chair
5	Dead

Eastern Cooperative Oncology Group, Robert Comis M.D., Group Chair (Oken et al. 1982).

Appendix 3: Highly Effective Methods of Birth Control

For females of childbearing potential (defined as <2 years after last menstruation or not surgically sterile), a negative serum or urine pregnancy test must be documented at screening, prior to pre-conditioning and confirmed before receiving the first dose of study treatment.

For females who are not postmenopausal (<24 months of amenorrhea) or who are not surgically sterile (absence of ovaries and/or uterus), two methods of contraception comprising of one highly effective methods of contraception together with a barrier method must be used during the treatment period and for at least 12 months after the last dose of study treatment. They must agree not to donate eggs (ova, oocytes) for the purposes of assisted reproduction during the study and for 12 months after receiving the last dose of study drug.

For males, it must be agreed that two acceptable methods of contraception are used (one by the patient – usually a barrier method, and one highly effective method by the patient's partner) during the treatment period and for at least 12 months after the last dose of study treatment and that sperm will not be donated during the treatment period and for at least 12 months after the last dose of study treatment.

Highly effective methods of birth control

For the purpose of this guidance, methods that can achieve a failure rate of less than 1% per year when used consistently and correctly are considered as highly effective birth control methods. Such methods include:

- Combined (oestrogen and progestogen containing) hormonal contraception associated with inhibition of ovulation[1]:
  - oral
  - intravaginal
  - transdermal
- Progestogen-only hormonal contraception associated with inhibition of ovulation [1]:
  - oral
  - injectable
  - implantable[2]
- Intrauterine device[2]
- Intrauterine hormone-releasing system[2]
- Bilateral tubal occlusion[2]
- Vasectomised partner[2, 3]
- Sexual abstinence[4]

1 Hormonal contraception may be susceptible to interaction with the advanced therapy investigational medicinal product, which may reduce the efficacy of the contraception method.

2 Contraception methods that in the context of this guidance are considered to have low user dependency.

3 Vasectomised partner is a highly effective birth control method provided that partner is the sole sexual partner of the women of child bearing potential trial participant and that the vasectomised partner has received medical assessment of the surgical success.

4 In the context of this guidance sexual abstinence is considered a highly effective method only if defined as refraining from heterosexual intercourse during the entire period of risk associated with the study treatments.

The reliability of sexual abstinence needs to be evaluated in relation to the duration of the clinical trial and the preferred and usual lifestyle of the subject.

Birth control methods which may NOT be considered as highly effective

Acceptable birth control methods that result in a failure rate of more than 1% per year include:

- Progestogen-only oral hormonal contraception, where inhibition of ovulation is not the primary mode of action
- Male or female condom with or without spermicide[5]
- Cap, diaphragm or sponge with spermicide[5]

[5] A combination of male condom with either cap, diaphragm or sponge with spermicide (double barrier methods) are also considered acceptable, but NOT highly effective, birth control methods.

Birth control methods which are considered UNACCEPTABLE in clinical trials

- Periodic abstinence (calendar, symptothermal, post-ovulation methods)
- Withdrawal (coitus interruptus)
- Spermicides only
- Lactational amenorrhoea methods are not acceptable methods of contraception.
- Female condom and male condom should not be used together.

Acceptable methods of birth control in clinical trials:

- Total abstinence (when this is in line with the preferred and usual lifestyle of the patient). Periodic abstinence (e.g., calendar, ovulation, symptothermal, post-ovulation methods) and withdrawal are NOT acceptable methods of contraception.
- Female sterilisation (have had surgical bilateral oophorectomy with or without hysterectomy) or tubal ligation at least 6 weeks before taking study treatment. In case of oophorectomy alone, only when the reproductive status of the woman has been confirmed by follow-up hormone level assessment.
- Male sterilisation (at least 6 months prior to screening). For female patients on the study the vasectomised male partner should be the sole partner for that patient.
- BOTH of the following forms of contraception must be utilised:
    a. Use of oral, injected or implanted hormonal methods of contraception or other forms of hormonal contraception that have comparable efficacy (failure rate <1%), for example hormone vaginal ring or transdermal hormone contraception.
    b. Barrier methods of contraception: condom or occlusive cap (diaphragm or cervical/vault caps) with spermicidal foam/gel/film/cream/vaginal suppository.
- Use of IUDs are excluded due to increased risks of infection and bleeding in this population.
- In case of use of oral contraception, women must be stable on the same pill for a minimum of 3 months before taking study treatment.

Women who are not of reproductive potential (defined as either <11 years of age, Tanner Stage 1, post-menopausal for at least 24 consecutive months or have undergone hysterectomy, salpingectomy, and/or bilateral oophorectomy) are eligible without requiring

the use of contraception. Acceptable documentation includes written or oral documentation communicated by clinician or clinician's staff of one of the following:

- Demographics show age <11 years.
- Physical examination indicates Tanner Stage 1.
- Physician report/letter.
- Operative report or other source documentation in the patient record.
- Discharge summary.
- Follicle stimulating hormone measurement elevated into the menopausal range.
